# Sex differences in multilayer functional network topology over the course of aging in 37543 UK Biobank participants

**DOI:** 10.1162/netn_a_00286

**Published:** 2023-01-01

**Authors:** Mite Mijalkov, Dániel Veréb, Oveis Jamialahmadi, Anna Canal-Garcia, Emiliano Gómez-Ruiz, Didac Vidal-Piñeiro, Stefano Romeo, Giovanni Volpe, Joana B. Pereira

**Affiliations:** Department of Neurobiology, Care Sciences and Society, Karolinska Institutet, Stockholm, Sweden; Department of Molecular and Clinical Medicine, Goteborg University, Goteborg, Sweden; Department of Physics, Goteborg University, Goteborg, Sweden; Department of Psychology, University of Oslo, Oslo, Norway; Cardiology Department, Sahlgrenska University Hospital, Gothenburg, Sweden; Clinical Nutrition Unit, University Magna Graecia, Catanzaro, Italy; Memory Research Unit, Department of Clinical Sciences Malmö, Lund University, Lund, Sweden

**Keywords:** Aging, Sex differences, Multilayer networks, Functional connectivity, Anti-correlations

## Abstract

Aging is a major risk factor for cardiovascular and neurodegenerative disorders, with considerable societal and economic implications. Healthy aging is accompanied by changes in functional connectivity between and within resting-state functional networks, which have been associated with cognitive decline. However, there is no consensus on the impact of sex on these age-related functional trajectories. Here, we show that multilayer measures provide crucial information on the interaction between sex and age on network topology, allowing for better assessment of cognitive, structural, and cardiovascular risk factors that have been shown to differ between men and women, as well as providing additional insights into the genetic influences on changes in functional connectivity that occur during aging. In a large cross-sectional sample of 37,543 individuals from the UK Biobank cohort, we demonstrate that such multilayer measures that capture the relationship between positive and negative connections are more sensitive to sex-related changes in the whole-brain connectivity patterns and their topological architecture throughout aging, when compared to standard connectivity and topological measures. Our findings indicate that multilayer measures contain previously unknown information on the relationship between sex and age, which opens up new avenues for research into functional brain connectivity in aging.

## Author Summary

Alterations in the resting-state functional connectivity patterns are a hallmark of the aging process. However, there is little agreement on how sex affects these age-related functional trajectories. Here, we propose novel multilayer measures that capture the relationship between positive and negative connections. We show that they are more sensitive to sex-related changes in the whole-brain connectivity patterns and their topology throughout aging, when compared to standard connectivity and topological measures. Our results suggest that multilayer measures provide information on the interaction between sex and age that has not been previously explored, which leads to a more accurate evaluation of cognitive, structural, and cardiovascular risk factors, and provides new insights into the genetic influences on changes in functional connectivity that occur throughout aging.

## INTRODUCTION

Although the human life span has increased from 50 to 80 years of age in the past two centuries, this has not been matched by an improvement in health span (Crimmins, 2015). In fact, age is one of the major risk factors for debilitating conditions such as cardiovascular and neurodegenerative diseases, having a large societal and economic impact (Dhingra & Vasan, 2012; Hou et al., 2019). However, not all individuals age in the same way. In particular, sex seems to be responsible for a substantial interindividual variability during aging, with women displaying a higher probability of developing certain age-related disorders such as Alzheimer’s disease (Mazure & Swendsen, 2016) and multiple sclerosis (Golden & Voskuhl, 2017), whereas men are more likely to develop Parkinson’s disease (K. M. Smith & Dahodwala, 2014). These differences in vulnerability to distinct diseases suggest that men and women have a distinct underlying brain network organization that might predispose them to develop specific pathological processes.

The functional network organization of the brain can be assessed using the correlations of spontaneous fluctuations in brain activity across brain regions by measuring the blood oxygen level–dependent signals on resting-state functional magnetic resonance imaging (rs-fMRI) (B. Biswal, Zerrin Yetkin, Haughton, & Hyde, 1995). Using this technique, several studies have identified highly reproducible resting-state networks in the brain such as the sensorimotor, dorsal attention, and default-mode networks, which play an important role in motor and cognitive functions (van den Heuvel & Pol, 2010). The communication between these networks is particularly important for brain function and has been shown to change during the course of aging (Betzel et al., 2014; Sala-Llonch, Bartrés-Faz, & Junqué, 2015), with older individuals showing a loss of anticorrelations (negative connections) (Keller et al., 2015; Spreng, Stevens, Viviano, & Schacter, 2016) and increases in positive correlations (positive connections) (Damoiseaux, 2017; Ferreira et al., 2016) between resting-state networks. These changes reflect the tendency of older individuals to over-recruit functional networks, needing to activate more brain networks than younger individuals, thus decreasing functional specialization and spending more neural resources (Goldstone et al., 2016).

However, the impact of sex on the communication between functional brain networks during aging is still not well understood (Ritchie et al., 2018; Scheinost et al., 2015), in part due to the small number of participants included in previous studies and their limited statistical power (Ritchie et al., 2018; Ruigrok et al., 2014). Assessing sex differences in functional connectivity is important for several reasons. For example, because functional networks are closely associated with cognitive and sensorimotor functions (van den Heuvel & Pol, 2010), understanding how their communication deteriorates with aging might provide important clues on why men and women are vulnerable to different diseases (Golden & Voskuhl, 2017; Mazure & Swendsen, 2016; K. M. Smith & Dahodwala, 2014) and why they show differences in other important health aspects such as brain structure, cardiovascular risk factors, and cognitive function (McCarrey, An, Kitner-Triolo, Ferrucci, & Resnick, 2016; Ramirez & Sullivan, 2018; Ritchie et al., 2018; Sachdev, Parslow, Wen, Anstey, & Easteal, 2009; Weiss, Kemmler, Deisenhammer, Fleischhacker, & Delazer, 2003).

From a methodological point of view, studies analyzing functional connectivity have mainly focused on positive connections (Chan, Park, Savalia, Petersen, & Wig, 2014; Tomasi & Volkow, 2012a). While this approach is more straightforward to assess the organization or topology of brain networks (Fornito, Zalesky, & Breakspear, 2013), negative connections are commonly found between brain networks or areas and seem to play an important role in brain communication (Fox et al., 2005; Hampson, Driesen, Roth, Gore, & Constable, 2010) and cognition (Barber, Caffo, Pekar, & Mostofsky, 2013). Since the negative connections or anticorrelations carry behaviorally relevant information (Barber et al., 2013), an integrative approach that incorporates information from negative correlations as well as positive correlations may reveal unique insights on sex differences throughout aging.

In this study, we developed this approach by combining the positive and negative functional connections between networks as separate layers in a complex multilayer network. We demonstrated that the multilayer measures provide novel insights about the impact of age and sex on the different functional connectivity trajectories in men and women between 47 and 79 years old in a large cross-sectional cohort of 37,543 individuals. Moreover, together with other measures of network organization, they were associated with structural brain imaging markers, cardiovascular risk factors, and cognitive functions, which typically differ between men and women (McCarrey et al., 2016; Ramirez & Sullivan, 2018; Ritchie et al., 2018; Sachdev et al., 2009; Weiss et al., 2003), as well as genes involved in physiological processes associated with aging. These findings open new avenues for the study of functional brain connectivity in aging by using multilayer network measures.

## RESULTS

### Sample

We included 19,975 women and 17,568 men with resting-state functional MRI from the UK Brain Biobank cohort (Miller et al., 2016) (*Methods*, section *Participants* and Supporting Information Figure S1). A subset of these individuals had available structural brain imaging data such as T1-weighted imaging and underwent a comprehensive assessment of cognitive functions and cardiovascular risk factors (*Methods*, sections *Structural Imaging Preprocessing*, *Cardiovascular Risk Factors*, and *Cognitive Tests*). Using permutation testing to compare the demographic characteristics between women and men, we found that men showed higher scores than women in the executive cognitive domain during middle ages and in the visuospatial cognitive domain across all ages (Supporting Information Figure S2C and D). In line with previous research showing that men are more susceptible to cardiovascular problems during middle adulthood (Anand et al., 2008; Gillis & Sullivan, 2016), we observed a significantly greater prevalence of high blood pressure, heart attack and white matter hyperintensities in men between the ages of 51 and 76 compared to women (Supporting Information Figure S2E, F, K). Finally, men had larger subcortical volumes across all ages (Supporting Information Figure S2I–K), consistent with previous studies (Ritchie et al., 2018; Wang, Xu, Luo, Hu, & Zuo, 2019). There were no significant differences between sexes in professional qualifications and years of education.

### Brain Connectivity Analysis

**Figure 1.**
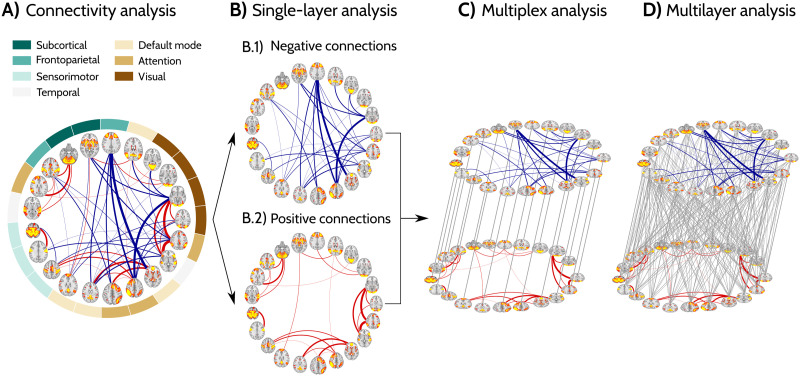
Analysis workflow. (A) Example of the 21 resting-state networks used as nodes and their positive (red) and negative connections (blue) for one of the subjects included in the analyses. Each network was identified based on previous descriptions (Miller et al., 2016). (B) The positive and negative connections were split into two networks: one negative (b.1) and the other one positive (b.2). The topology of these two networks was evaluated using the clustering coefficient and global efficiency. (C and D) The positive and negative networks were then integrated as two separate layers in a multiplex network (C) (where each node in one layer is connected to the same node in the other layer) and a multilayer network (D) (where each node in one layer is connected to all other nodes in the other layer). We evaluated the topology of the multiplex network using the clustering and participation coefficients, whereas the topology of the multilayer network was assessed using the novel multilayer global efficiency and multilayer clustering coefficient. In all graphs, thicker connections represent stronger positive or negative functional connections.

Functional brain connectivity was assessed for each participant using the negative and positive correlations between 21 nodes that correspond to the resting-state functional MRI networks shown in Figure 1A and Supporting Information Figure S3 (*Methods*, section *Functional Image Preprocessing*). First, we computed classical single-layer connectivity measures, namely, the average connectivity for the whole correlation network, followed by the average negative connectivity, the average positive connectivity, and the number of negative correlations (*Methods*, section *Connectivity Measures*). Then, the negative and positive correlations of each functional network were separated (Figure 1B.1 and 1B.2) and analyzed as two independent layers. To evaluate the topological organization of each single layer, we used two measures: the clustering coefficient and the global efficiency. The clustering coefficient is a measure of segregation that increases with the number of local connections and represents the average clustered connectivity around all nodes in the network. The global efficiency is a measure of integration that increases as the paths connecting any two nodes in the network become shorter and estimates the average capability with which different nodes communicate with each other (Mijalkov et al., 2017) (*Methods*, section *Single-Layer Network Measures*). To assess the relationship between these two layers, we integrated them into a multiplex network (Figure 1C) and a multilayer network (Figure 1D). In the multiplex network approach (Figure 1C), each node in the positive layer was connected with the same node in the negative layer. We computed two multiplex measures: the multiplex clustering coefficient, a measure that increases with the local connections in neighboring nodes between the two layers, and the multiplex participation, which is a measure of integration that assesses how evenly a node is connected in the two layers (Battiston, Nicosia, & Latora, 2014) (*Methods*, section *Multiplex Network Measures*). A disadvantage of the multiplex approach is that the relation between the two layers is local being only allowed between the same nodes. To address this limitation, in the multilayer network approach we connected each node in one layer to every node in the other layer (Figure 1D). The strength of the relationship between the two layers can be changed by adjusting the weight of the interlayer connections, *σ*. For each multilayer network, we define *σ* as a fraction of the strongest functional connections in the corresponding network, and evaluate the measures’ ability to characterize sex differences across the wide range of *σ* values. We developed two new measures to assess the integration and segregation properties of these multilayer networks. Specifically, we calculated the multilayer global efficiency, which compares the global efficiency differences due to the intra- and interlayer connections. Similarly, we also calculated the multilayer clustering coefficient, which compares the clustering coefficients or triangles in all nodes between the two layers (*Methods*, section *Multilayer Network Measures*).

### Women Have Less Negative Connections Than Men

**Figure 2.**
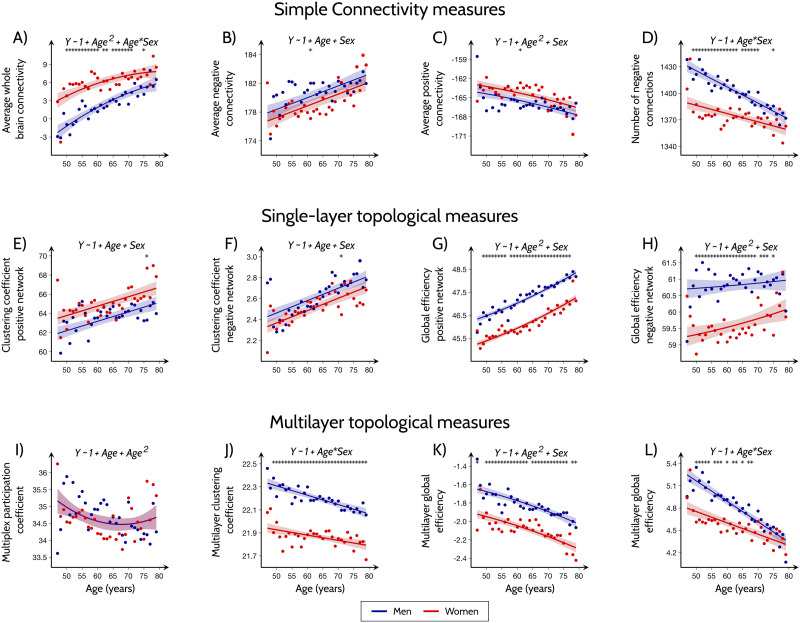
Functional connectivity dependence on age and sex. Results of the linear models with simple connectivity measures (A–D), single-layer topological measures (E–H) and multi-layer topological measures (I–J, multiplex; K–L, multilayer) as the outcomes and age, sex, age^2^, age × sex and age^2^ × sex interactions as predictors. The areas show the 95% confidence intervals (CI) for the predictions and the solid lines show the best line fit. The stars indicate points that showed significant differences between men and women after correction for multiple comparisons across the different age groups (FDR at *q* < 0.05). The dots show the average values for men and women at the corresponding age. For each measure, only the best fitting model containing a subset of the predictors is shown. Details about the best fitting, as well as the full model which includes all predictors, are shown in *Supporting Information: Linear Models*. Detailed report of statistics are shown in *Supporting Information: Statistical Comparisons* and *Supporting Information: Power Analysis*, see also *Methods*, section *Statistical Analysis* and Supporting Information Figure S4.

In a first step, to identify which simple connectivity measures showed the greatest differences between sexes over the course of aging, we compared men and women at all ages using a permutation test and used separate linear models that included whole-brain average connectivity, average negative connectivity, average positive connectivity, and the number of negative correlations as the outcome and age, sex, age^2^, age × sex, and age^2^ × sex interactions as predictors (the models were fit on the average data by sex and within each age; *Methods*, section *Statistical Analysis*). These models showed that women had significantly higher average functional connectivity than men (*R*^2^ = 0.769; AIC = 244.651; *MSE* = 2.184; Figure 2A and Supporting Information Figure S4A), whereas men had a significantly higher number of negative connections compared to women (*R*^2^ = 0.778; AIC = 501.106; *MSE* = 108.420; Figure 2D and Supporting Information Figure S4D) across a broad age range (50 to 71 years). These differences in connectivity between sexes diminished with increasing age, to the point where there were almost no differences in mean connectivity strength or number of negative connections between men and women after 75 years (age × sex interaction: average connectivity *p* = 0.01; number of negative connections *p* < 0.001). On the other hand, there were no significant differences between sexes in the average positive and negative connectivity strength (*R*^2^ = 0.403; AIC = 281.229; *MSE* = 3.947 and *R*^2^ = 0.408; AIC = 281.229; *MSE* = 2.013, respectively; Figure 2B and C and Supporting Information Figure S4B and C). These results suggest that women have higher connectivity strengths than men due to a lower number of negative connections, but these differences dissipate with increasing age.

### Men Have Shorter Network Paths Than Women

To identify which single-layer topological measures showed the greatest differences between sexes over the course of aging, we repeated the above analysis by including them as dependent variables in separate linear models with age, sex, age^2^, age × sex, and age^2^ × sex interactions as predictors. These models showed that men had higher global efficiency than women in the positive and negative layers (*R*^2^ = 0.924; AIC = −1.051; *MSE* = 0.055 and *R*^2^ = 0.649; AIC = 88.603; *MSE* = 0.209, respectively; Figure 2G–H and Supporting Information Figure S4G–H), indicating that their functional connectomes were characterized by shorter paths in the networks with negative and positive connections. Interestingly, these sex differences remained constant across different ages, suggesting that they were independent of age (age × sex or age^2^ × sex interaction not significant in global efficiency measures; Supporting Information: Linear Models). In contrast, no significant differences in the clustering coefficients in the positive and negative networks were observed between women and men (*R*^2^ = 0.459; AIC = 226.729; *MSE* = 1.728 and *R*^2^ = 0.533; AIC = −96.294; *MSE* = 0.013, respectively; Figure 2E and F and Supporting Information Figure S4E and F).

### Men Have a Greater Balance Between Positive and Negative Connections

Our linear regression models showed that the multiplex participation coefficient was significantly higher in men than in women across all ages (*R*^2^ = 0.891; AIC = −172.848; *MSE* = 0.004; Figure 2J, Supporting Information Figure S4J and Supporting Information: Linear Models), indicating that the men’s functional connectomes were characterized by nodes with a similar number of connections in the negative and positive connectivity layers. However, these differences decreased with age, similarly to the average connectivity and number of negative connections (age × sex interaction: *p* = 0.036). The multiplex clustering coefficient did not reveal any significant differences between men and women (*R*^2^ = 0.096; AIC = −106.989; *MSE* = 0.282; Figure 2I and Supporting Information Figure S4I). We note that caution needs to be applied when considering multiplex clustering coefficients as their interpretation can be cumbersome for multiplex correlation networks (Masuda, Sakaki, Ezaki, & Watanabe, 2018; Zalesky, Fornito, & Bullmore, 2012).

### Multilayer Topological Measures Are Lower in Women Than in Men

The differences between men and women were robust for the complete range of interlayer weights *σ* for the multilayer clustering and for smaller values of *σ* in the case of multilayer global efficiency. However, the strongest differences were observed for the *σ* = 0.7 and *σ* = 0.2 in the case of multilayer clustering and multilayer global efficiency, respectively (Supporting Information Figures S5 and S6). Whereas the multilayer clustering coefficient differences between men and women remained stable with aging (*R*^2^ = 0.799; AIC = −130.497; *MSE* = 0.008; age × sex interaction not significant; Figure 2K and Supporting Information: Linear Models), the differences in multilayer global efficiency decreased with aging (*R*^2^ = 0.795; AIC = −82.061; *MSE* = 0.016; age × sex interaction: *p* < 0.001; Figure 2L and Supporting Information: Linear Models). Up to 80% of the variance in both multilayer measures was associated with sex differences over time, outperforming all previous models for simple connectivity, single-layer measures, and multiplex topological measures. For a more consistent comparison of the different measures, we have repeated our analysis when the functional connectivity metrics were fitted to a full model with age, sex, age^2^, age × sex and age^2^ × sex) as predictors (Supporting Information: Linear Models). These results further confirm that multilayer measures and multiplex participation are the best performing measures and suggest that complex network measures that account for the relationship between positive and negative functional connections are more sensitive to sex differences across aging.

Finally, we have conducted several analyses to assess the reproducibility of our findings. Specifically, we assessed the reproducibility of the multilayer measures as a function of sample size (Marek et al., 2022) (Supporting Information: Figures, Section VII), whether they were replicable at individual network densities (Supporting Information: Figures, Section VIII), as well as their test-retest reliability using the intraclass correlation coefficient (ICC) (McGraw & Wong, 1996) (Supporting Information: Test-Retest). Our findings showed that multilayer measures can uncover similar patterns of between-sex differences for different analysis parameters. Specifically, at most ages, they can detect the differences observed in the original sample in subsamples with sizes as low as 55%–60% of the original sample, as well as at individual network densities higher than 15%. Finally, their test-retest reliability was greatest in younger individuals and higher than that of test-retest reliability of single-layer and connection measures; their ICC values ranged from 0.4 to 0.65, suggesting a fair to good degree of clinical significance (Cicchetti & Sparrow, 1981).

### Multilayer and Multiplex Measures Are Significant Explainers of Structural, Cognitive and Cardiovascular Differences Between Men and Women

**Figure 3.**
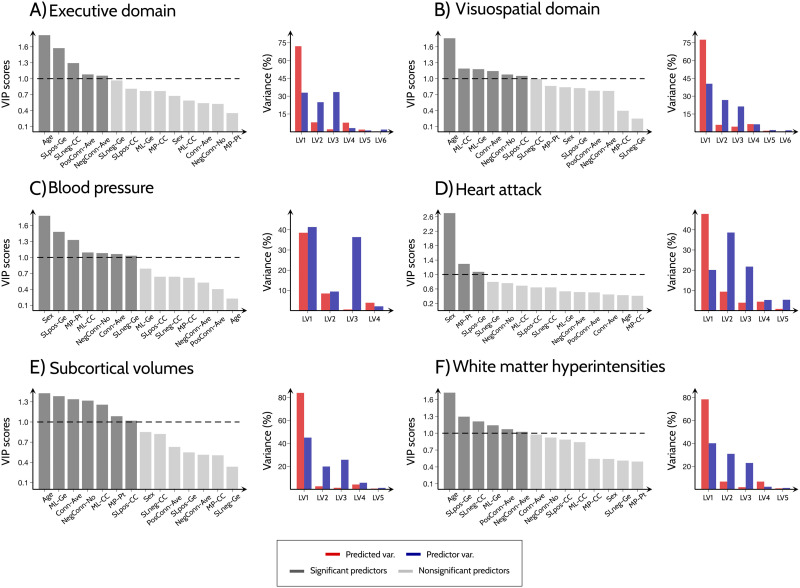
Relationship between functional connectivity measures and brain structure, cognitive measures and cardiovascular disease factors. Plots showing the VIP scores for all functional connectivity measures and the amount of variance (for predicted and predictor variables) explained by the corresponding latent variables. The PLS analysis was performed for measures showing significant differences between men and women in (A) executive and (B) visuospatial domains; (C) prevalence of high blood pressure and (D) heart attack; (E) subcortical volumes and (F) white matter hyperintensities. Abbreviations: LV: latent variables; Conn-Ave: Average connectivity; PosConn-Ave: Average positive connectivity; NegConn-Ave: Average negative connectivity; NegConn-No: Number of negative connections; SLpos-CC and SLneg-CC: Single-layer clustering coefficient for networks of positive and negative connections; SLpos-Ge and SLneg-Ge: Single-layer global efficiency for networks of positive and negative connections; MP-CC: Multiplex clustering coefficient; MP-Pt: Multiplex participation coefficient; ML-CC and ML-Ge: Multilayer clustering coefficient and global efficiency.

Next, we assessed whether the observed sex differences in functional connectivity throughout aging were associated with the cognitive functions, structural brain measures and vascular risk factors that differed between men and women in our cohort (executive functions, visuospatial functions, blood pressure and heart attack prevalence, subcortical volumes, white matter hyperintensities; see *Results*, section *Sample*, and Supporting Information Figure S2). Due to the high collinearity between the functional connectivity measures (Supporting Information Figure S7), we examined these associations using partial least squares (PLS) regressions. The executive cognitive scores were best explained by the single-layer positive global efficiency, followed by the single-layer negative clustering, average positive connectivity and negative connectivity. The visuospatial cognitive scores were best explained by the multilayer functional connectivity measures, followed by the average connectivity, number of negative connections, and the positive single-layer global efficiency. Heart attack and high blood pressure prevalence were best predicted by multiplex participation and single-layer global efficiency of the positive connections, with blood pressure additionally being predicted by multilayer clustering coefficient, number of negative connections, average connectivity, and single-layer global efficiency of the positive network. Multilayer global efficiency was a significant predictor of subcortical brain volumes and white matter hyperintensities, in addition to the average connectivity, number of negative connections, multilayer clustering coefficient, multiplex participation, and single-layer clustering coefficient in the case of subcortical volumes and single-layer clustering coefficient in the case of white matter hyperintensities. The detailed VIP scores for each predictor and the PLS model cumulative explained variance for the predicted variables are shown in Figure 3 and Supporting Information: Linear Models. Altogether, these results indicate that, compared to the other measures, the multilayer and multiplex measures were the best predictors of cognitive functions, structural brain measures, and vascular risk factors that differed between men and women during aging.

### Multilayer and Multiplex Measures Are Associated With Genes Involved in Aging-Related Physiological Processes

**Figure 4.**
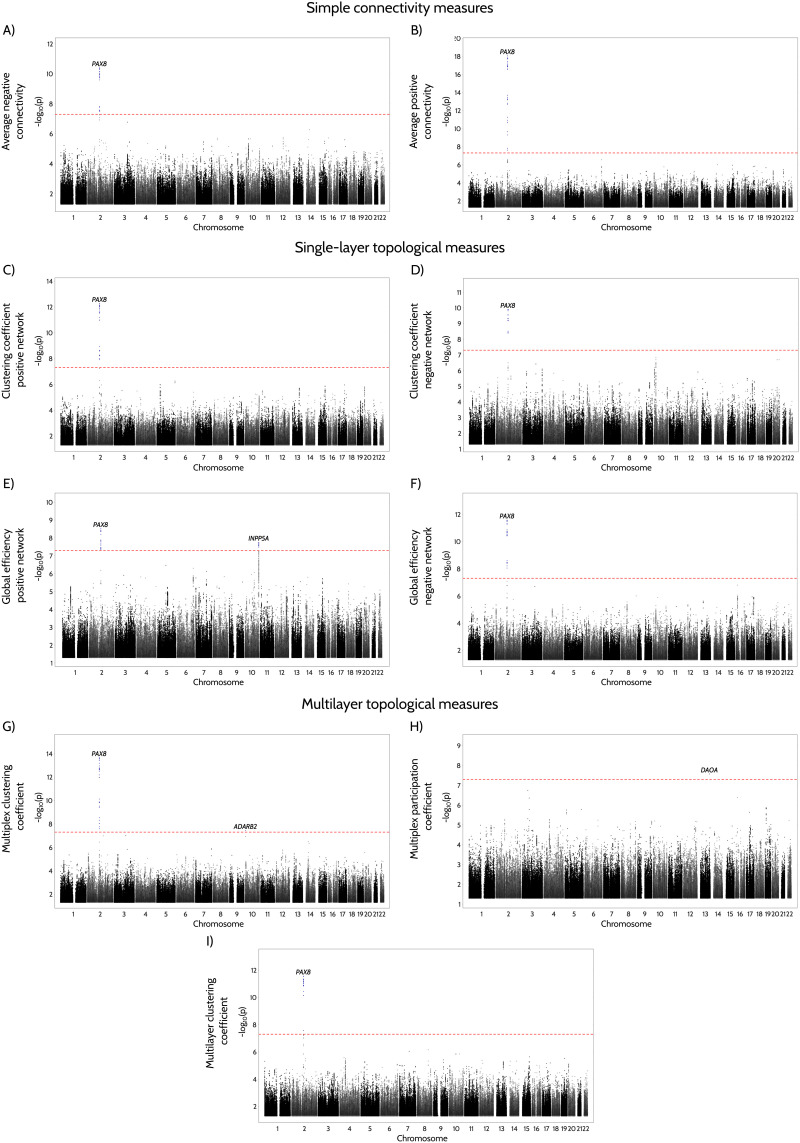
Manhattan plots for GWAS analysis of functional connectivity measures. The results from the GWAS analysis of simple connectivity measures (A–B), single-layer topological measures (C–F) and multi-layer topological measures (G–H, multiplex; I, multilayer) in a total sample of 33,773 individuals. The *y*-axis shows the *p* values for the association tests on a −log10 scale, while the different chromosomes (differentiated by the black and gray colors) are ordered on the *x*-axis. The lead variants that surpass the genome-wide significance threshold (indicated by the red line) are highlighted as blue circles.

To identify genetic variation associated with the 12 multilayer, single-layer and connectivity functional measures we examined 9,356,431 genetic variants with minor allele frequency > 1% in a total of 33,773 European participants (*Methods*, section *Genetic Association Analyses*). A total of 4 loci exceeded a genome-wide significance level among one or more of these traits (Figure 4 and Supporting Information Figure S8). A locus near Paired Box 8 (*PAX8*), a gene involved in sleep efficiency, diastolic blood pressure, development, and vulnerability to neurodegenerative diseases (Elliott et al., 2018; Foo et al., 2021b; Jones et al., 2019), showed an association with the majority of functional connectivity measures, including multilayer and multiplex clustering, as well as all single-layer measures. Colocalization analysis with eQTL summary statistics of 49 tissues in GTEx project and brain tissues in BrainSeq, ROSMAP, Braineac2, and CommonMind datasets (*Methods*, section *Colocalization*) (GTEx Consortium, 2020; Guelfi et al., 2020; Jaffe et al., 2018; Kerimov et al., 2021; Ng et al., 2017; Sieberts et al., 2020), suggests a consistent strong colocalization between this locus and gene expression patterns of Immunoglobulin Kappa Variable 1/OR2-108 (*IGKV1OR2-108*), COBW domain-containing protein 2 (*CBWD2*), and Forkhead Box D4 Like 1 (*FOXD4L1*) over the multilayer and multiplex clustering coefficients, average positive and negative connectivity, and all single-layer functional measures (Supporting Information: Genetic Results). Furthermore, a locus near Inositol Polyphosphate-5-Phosphatase A (*INPP5A*) gene showed significant associations with the single-layer positive global efficiency. Interestingly, the genome-wide association study (GWAS) lead variant at this locus colocalized with eQTL for *INPP5A* in brain dorsolateral prefrontal cortex (DLPFC) from CommonMind dataset. Finally, loci near Adenosine Deaminase RNA specific B2 (*ADARB2*) and D-Amino Acid Oxidase Activator (*DAOA*) genes, which have previously been linked with processes involved in normal memory functioning and ATP metabolism (Prata et al., 2012), were associated with multiplex clustering and multiplex participation coefficients respectively.

## DISCUSSION

Complex network measures are becoming increasingly popular in the field of network science (van den Heuvel & Pol, 2010). In combination with large samples, these measures can improve our understanding of brain connectivity (Fornito et al., 2013), and, in particular, our ability to predict sex differences over the course of aging (Stumme, Jockwitz, Hoffstaedter, Amunts, & Caspers, 2020). Previous studies of brain connectivity and network topology have mainly focused on measures that exclude the negative connections or average the effects of positive and negative connections (Chan et al., 2014; Ferreira et al., 2016; Foo et al., 2021a; Tomasi & Volkow, 2012b; Zhang et al., 2016). However, changes in the balance between positive and negative connectivity could be a more sensitive marker of abnormalities that occur in men and women in middle and late adulthood. Here we show that measures that assess this balance are indeed better predictors of sex differences during aging. Furthermore, we also show that these measures are associated with genes implicated in aging-related physiological processes as well as cognition, brain structure, and vascular disease, which have been previously shown to differ between men and women (McCarrey et al., 2016; Ramirez & Sullivan, 2018; Ritchie et al., 2018; Sachdev et al., 2009; Weiss et al., 2003). Altogether these findings highlight the importance of integrating the information from positive and negative connections in a multiplex or multilayer network approach to provide a more holistic view of functional brain connectivity changes.

Increasing age is associated with lower physical fitness and worse cognitive abilities, being one of the greatest risk factors for the development of neurodegenerative diseases (Deary et al., 2009; Wyss-Coray, 2016). However, there are several factors that determine how an individual ages and his/her predisposition to develop certain diseases. Sex is one of these factors and it has recently received a lot of attention due to increasing recognition that precision medicine approaches should take into account biological sex to treat neurodegenerative diseases (Ferretti et al., 2018). Sex was found to affect the functional connectivity (Allen et al., 2011; B. B. Biswal et al., 2010; Ritchie et al., 2018); however, the location and nature of the functional connectivity differences between men and women vary across studies (Scheinost et al., 2015). Between-sex differences were most consistently observed in the default mode network (Allen et al., 2011), but also reported in other resting-state networks (Allen et al., 2011; B. B. Biswal et al., 2010; Filippi et al., 2013; Scheinost et al., 2015), as well as in the functional connectivity patterns between them (Goldstone et al., 2016; Satterthwaite et al., 2015; Stumme et al., 2020). These differences between men and women change with aging (Cosgrove, Mazure, & Staley, 2007), with studies showing different age-related trajectories in functional connectivity that can be either diverging or converging in different resting-state networks (Goldstone et al., 2016; Scheinost et al., 2015). However, other studies have reported that between-sex differences persist also in older individuals (Stumme et al., 2020). Such differing results suggest that the understanding of sex differences in the adult brain is still in its infancy and utilizing larger samples or more sensitive methods are needed to address these discrepancies.

Functional brain connectivity plays a crucial role in how brain networks communicate with each other, being closely associated with behavior and cognition (van den Heuvel & Pol, 2010). Studies have shown that aging is accompanied with an increase in the connections between these networks (Betzel et al., 2014; Chan et al., 2014; Damoiseaux, 2017; Ferreira et al., 2016). These increases in connectivity are thought to be due to a less efficient use of neural resources in older individuals, who tend to over-recruit brain networks to compensate for the detrimental effects of aging (Geerligs, Renken, Saliasi, Maurits, & Lorist, 2015; Goldstone et al., 2016; Park & Reuter-Lorenz, 2009). Here, we confirm these findings in a larger sample of middle-age and old adults by showing that aging is associated with increases in the average whole-brain connectivity and decreases in the number of negative connections. Moreover, our findings revealed that men displayed lower average connectivity and a greater number of negative connections than women at young ages, but these differences dissipated with increasing age, to the point that no differences between sexes were observed any longer at old ages. These findings agree with results from previous studies showing different functional trajectories between men and women (Satterthwaite et al., 2015; Scheinost et al., 2015). However, in contrast to other studies (Stumme et al., 2020), here we demonstrate that, although the functional connectomes of younger men and women are different, they become increasingly similar with older age, possibly due to a faster rate of functional changes observed in the brains of men (Foo et al., 2021a), which is in line with studies showing that men have lower resilience to age-related cognitive decline compared to women (McCarrey et al., 2016).

Regarding measures of network organization, we found that the positive global efficiency was higher in men compared to women, but these differences remained stable across different ages. Similar differences were found in the negative global efficiency; however, in this case, the differences were not stable and seemed to decrease with aging. The global efficiency is used to assess integration or the ability for an efficient processing to occur between distant brain networks (Fornito et al., 2013); an excessive integration is thought to impede the ability of the brain to process information in a meaningful way (Lord, Stevner, Deco, & Kringelbach, 2017). Furthermore, global efficiency is associated with the presence of long range connections between networks. The healthy brain connectome is characterized by a low number of such connections because long range connections are associated with higher metabolic costs and minimizing these costs is essential for evolution (Bullmore & Sporns, 2012). Therefore, these differences between sexes might provide clues on why men are more prone to develop specific diseases with age, for example, Parkinson’s disease and epileptic seizures, which are associated with abnormal organization in the functional connectivity networks as a result of increased integration (Lord et al., 2017; Mijalkov, Volpe, & Pereira, 2022).

To evaluate whether the relationship between the positive and negative connections can reveal additional insights into sex differences over the course of aging, we integrated these connections as two separate layers in a multiplex and a multilayer networks. This relationship estimates the brain’s ability to inhibit certain functional connections in order to modulate the coactivation between different resting-state networks. Because the positive and negative connections can arise from different neurovascular mechanisms (Goelman, Gordon, & Bonne, 2014), the presence of both connection types is necessary to reach a more balanced communication between resting-state networks and consequently a better cognitive performance (Saberi, Khosrowabadi, Khatibi, Misic, & Jafari, 2021); in fact, the lack of anticorrelation or negative connections has been associated with lower cognitive control and working memory performance during neurodevelopment (Chai, Ofen, Gabrieli, & Whitfield-Gabrieli, 2014). This balance is disrupted in older adults, which over-recruit the functional networks as a compensatory mechanism to maintain or improve function (Goldstone et al., 2016; Park & Reuter-Lorenz, 2009), for example, by engaging both the left and right hemispheres (HAROLD model) (Cabeza, Anderson, Locantore, & McIntosh, 2002) or showing more distributed activation when compared to younger adults in performing verbal working and long-term memory as well as category-learning tasks (Park & Reuter-Lorenz, 2009).

In the multiplex network, where only connections between the same nodes in the two layers are allowed, we measured the balance between the positive and negative connections by the multiplex participation coefficient, which quantifies the heterogeneity of the connectivity patterns of a given node in both layers. We found that men had higher multiplex participation coefficients compared to women, indicating that they had a higher balance between positive and negative connections in the two layers that decreased with aging at a faster rate compared to women. Consistent with earlier findings, our results suggest that the less efficient connectomes reported in older persons may be related to an increase in the number of positive connections at the expense of negative connections (Ferreira et al., 2016), thereby disrupting the balance between coactivation and inhibition connectivity between functional networks.

The multilayer network approach, where connections are allowed between all nodes in the two layers, extends the multiplex approach by providing an estimation of the degree to which the between-layer balance affects the topological organization of the network. We found that men had higher multilayer clustering and multilayer global efficiency compared to women, which both decreased with aging. These findings indicate that the relation between positive and negative layers is stronger in men than in women. This might come at the expense of greater neural resources and metabolic costs, which could predispose the male connectome to the effects of increased oxidative stress and poor antioxidant defense mechanisms, which have been suggested to accompany higher brain connectivity (Griffa, Baumann, Thiran, & Hagmann, 2013) and could potentially lead to steeper rates of cognitive decline (McCarrey et al., 2016).

When the different connectivity and topological network measures were compared to each other, we found that the multiplex and multilayer network measures were the variables that were best predicted by sex differences over aging. In particular, age and sex were able to predict multiplex participation coefficient, multilayer clustering and multilayer global efficiency by explaining up to 89.1%, 79.9%, and 79.5% of the variance. These findings indicate that the integration of positive and negative connections as separate layers in a complex network approach is sensitive to important age- and sex-related variability not captured by conventional measures. This approach could thus be used to understand why men and women age differently. For instance, we found that cardiovascular risk factors such as hypertension and heart attack prevalence, which were higher in men between 51 and 79 ages, were best predicted by these measures. In addition, differences between sexes that remained stable over aging such as lower visuospatial cognitive scores and lower subcortical volumes in women compared to men were also best explained by multilayer measures.

Regarding the results of the GWAS analysis, a locus near the *PAX8* gene showed an association with the majority of functional connectivity measures. Interestingly, this locus has been associated with sleep efficiency, diastolic blood pressure, insomnia, and sleep length (Jones et al., 2019). These data are consistent with the aging process resulting in changes in sleep habits and high blood pressure (Ancoli-Israel, 2009; Ramirez & Sullivan, 2018), both of which have been linked to functional connectivity changes (Foo et al., 2021b; Neitzel et al., 2021; Tagliazucchi et al., 2012). The *PAX8* protein has also been associated to the regulation of multiple genes involved in thyroid hormone synthesis, which is necessary for brain development and function, for example, through processes such as neuronal differentiation, synaptogenesis, and dendritic proliferation (Foo et al., 2021b). All functional measures associated with the *PAX8* gene demonstrated substantial colocalization with the *FoxD4L1* gene, which is similarly implicated in processes that promote the onset of neural differentiation (Klein et al., 2013). They also demonstrated colocalization with *CBWD2*, which has been linked to sleep duration (Foo et al., 2021b), and *IGKV1OR2-108*, which has been found to be elevated in the livers of type 2 diabetes patients (Li, Pan, & Yang, 2019) and can lead to abnormal functional connectivity (Chen et al., 2014).

The *rs4309079* locus, associated with single-layer positive global efficiency, has been linked to functional connectivity measures in previous research (Elliott et al., 2018). It is located adjacent to the *INPP5A* gene, which is involved in calcium signaling. As a fundamental cellular mechanism, inositol calcium signaling is expected to play a role in a range of neurological pathways that underlie functional connectivity (Neitzel et al., 2021). The *INPP5A* gene has been further associated with brain age explained by changes in functional connectivity and decreased system segregation (Neitzel et al., 2021; S. M. Smith et al., 2020), which is consistent with our current findings.

Multiplex participation showed a strong association in a locus near the *DAOA* gene, which is known to play an important role in the control of glutamatergic transmission (Prata et al., 2012). Glutamate is the most common excitatory neurotransmitter in mammals, and glutamate’s activation of NMDA receptors is critical for normal memory function. Glutamatergic antagonists (e.g., ketamine) have been demonstrated to lower performance on tests of declarative memory, verbal fluency, and problem solving, all of which have been linked to aging (Prata et al., 2012). We nevertheless could not find any striking colocalization evidence in the examined gene expression QTL datasets. Finally, for multiplex clustering, we found a locus near the *ADARB2* (index variant: *rs2152237*) gene, which is involved in the ATP/ITP metabolic pathway. As a result, our findings add to our understanding of the genetic influences on functional connectivity and provide a link between the functional and genetic architecture of the brain, which might be relevant in explaining the changes in a variety of biological processes throughout healthy aging.

This study has some limitations. First, a longitudinal study design would have been more appropriate to assess age-related differences between sexes in functional brain connectivity. In particular, recent studies have shown that results regarding aging-related changes obtained from cross-sectional and longitudinal designs can be different from each other (Vidal-Piñeiro et al., 2021; Xing, 2021); therefore, our findings should be interpreted with this limitation in mind. However, multiple efforts to collect large longitudinal samples are currently underway (Liu et al., 2021; Miller et al., 2016), which opens the opportunity for future studies to overcome this limitation. Second, there was a considerable overlap between the values for individual men and women in functional connectivity measures. This overlap has also been observed in previous studies (Joel & Fausto-Sterling, 2016; Ritchie et al., 2018; Stumme et al., 2020; Zhang et al., 2016; Zhang, Dougherty, Baum, White, & Michael, 2018), indicating that the prediction of an individual’s sex from a small number of functional connectivity measures is challenging (Zhang et al., 2018). Nonetheless, our findings revealed consistent group differences between men and women across a wide age range (47–79 ages), suggesting that, although there is considerable variability, some changes seem to be quite robust (Chekroud, Ward, Rosenberg, & Holmes, 2016). Finally, since in this study the nodes of the networks corresponded to 21 resting-state networks derived from independent component analyses (Miller et al., 2016), the negative connections are related to anticorrelations between resting-state networks, which have been consistently observed and demonstrated to have a neurophysiological basis (Chai, Castañón, Öngür, & Whitfield-Gabrieli, 2012; Fox et al., 2005; Fox, Zhang, Snyder, & Raichle, 2009). Thus, these connections should be interpreted differently than the negative correlations between brain regions analyzed in studies using brain areas as nodes, which are still not clearly interpretable (Chai et al., 2012; Fornito et al., 2013).

To summarize, in this study we developed novel multilayer connectivity measures in order to assess the connectivity patterns and topological architecture between resting-state networks in a large cohort of middle age and old adults. We showed that these multilayer measures are superior at capturing sex-related effects during aging when compared to simpler connectivity measures that do not account for the relationship between positive and negative connections. The multilayer measures were also significant predictors of sex differences in cognitive, structural, and cardiovascular measures, and they were associated with genes that have previously been implicated in aging-related processes. Thus, our findings highlight the importance of studying the balance between positive and negative functional connections to understand the effects of sex over aging, which should be included in future studies.

## METHODS

### Participants

The UK Biobank cohort is a large population-based study with more than 500,000 participants from the United Kingdom (https://www.ukbiobank.ac.uk/). Following an initial visit for collection of medical and other clinical information, 37,704 individuals underwent MRI. To achieve robust group comparisons, we limited our analyses to age groups with at least 50 participants, resulting in a sample size of 37,543 people in the age range 47–79 years (17,568 men: mean age = 64.75; *SD* = 7.58 and 19,975 women: mean age = 63.49; *SD* = 7.33).

### Image Acquisition

The functional MRI scans were performed on a standard Siemens Skyra 3T scanner using an echo-planar imaging (EPI) sequence with the following parameters: duration ∼ 6 min; 490 time points; repetition time = 735 ms; echo time = 39 ms; field of view = 88 × 88 × 64; voxel size = 2.4 mm^3^; flip angle = 52°. Participants were instructed to relax and think of nothing in particular while focusing their eyes on a crosshair during the scan. Regarding structural MRI, the T1-weighted images were obtained using a 3D magnetization-prepared rapid gradient-echo imaging sequence with the following parameters: 208 slices, echo time = 880 ms; repetition time = 2,000 ms; field of view = 208 × 256 × 256; voxel size = 1 mm^3^ (Alfaro-Almagro et al., 2018).

### Image Preprocessing

All images obtained by the different imaging modalities were analyzed using an image-preprocessing pipeline run by the UK Biobank imaging core, who also performed quality assessment of the images (Alfaro-Almagro et al., 2018). In the following, we summarize the procedure for the different modalities; full details of the preprocessing and quality control are available elsewhere (Alfaro-Almagro et al., 2018; Miller et al., 2016).

### Functional Image Preprocessing

After data acquisition, a number of preprocessing steps were carried out using FSL, including motion correction using MCFLIRT, grand-mean intensity normalization by a single multiplicative factor, high-pass temporal filtering with a Gaussian-weighted least-squares straight line fitting (*σ* = 50.0 s), EPI unwarping by using a field map obtained before data collection, gradient distortion correction (GDC) unwarping, and removal of all artifacts by an ICA-based X-noiseifier. Finally, all datasets underwent temporal demeaning and variance normalization.

The preprocessed data of 4,100 participants was used for a Group-ICA analysis. Using FSL’s MELODIC tool and FSLNets toolbox (https://fsl.fmrib.ox.ac.uk/fsl/fslwiki), a spatial-ICA with a dimensionality of 25 components was applied and the resulting ICA maps were mapped onto each subject’s resting-state fMRI time series data to generate one representative timeseries per ICA component. During this procedure, four networks were identified as artifacts and were discarded from further analysis. This resulted in a 21 × 21 connectivity matrix for each participant, where the functional connectivity between each pair of ICA spatial maps is characterized by full normalized temporal correlation. Analyzed networks included the default mode, fronto-parietal, sensorimotor, visual, attention, subcortical, and temporal networks (Figure 1A); the group-ICA spatial maps can be found at https://biobank.ctsu.ox.ac.uk/crystal/refer.cgi?id=9028, and the average functional connectomes for several representative age groups are shown in Supporting Information Figure S2. The complete description of the preprocessing procedures can be found elsewhere (Alfaro-Almagro et al., 2018; Miller et al., 2016) (online documentation: https://biobank.ctsu.ox.ac.uk/crystal/docs/brain_mri.pdf).

### Structural Imaging Preprocessing

T1-weighted scans were preprocessed using the standard procedures of the FreeSurfer pipeline (version 6.0; https://surfer.nmr.mgh.harvard.edu/). We calculated the cortical thickness for each individual by averaging the regional cortical thicknesses from 68 regions from the Desikan–Killiany atlas (Desikan et al., 2006). In addition, we calculated the subcortical volumes for each individual by averaging the volumes of all subcortical gray matter structures (cerebellar cortex, thalamus, caudate, putamen, pallidum, hippocampus, amygdala, accumbens), which were corrected for total intracranial volume using a regression approach (O’Brien et al., 2011). This data was available for a subsample of 17,317 men and 19,842 women.

### White Matter Hyperintensities

The white matter hyperintensities were segmented from a combined T1 and T2-FLAIR images, using a fully automated supervised method based on the *k*-nearest neighbors algorithm (Griffanti et al., 2016).

### Cardiovascular Risk Factors

At their initial visit, individuals were questioned about their medical history, including whether or not they had a high blood pressure (subsample of 17,517 men and 19,911 women), a heart attack (subsample of 15,204 men and 16,918 women), a heart attack–related angina episode (subsample of 14,224 men and 15,520 women), or a stroke (subsample of 14,224 men and 15,520 women). We calculated the percentage of men and women diagnosed with the previous conditions at all age groups, and used these percentages as dependent variables in further analyses. Since the diagnosis of cardiovascular diseases was performed at a different time point than the brain scanning, we included the time difference between diagnosis and scanning as a covariate in the analyses with cardiovascular variables. Furthermore, as there were several age groups that did not have any individuals with heart attack and hypertension, the models for these variables were corrected for age prior to the PLS regression.

### Cognitive Tests

The cognitive assessments were administered on a touch screen and took place at the same visit as the brain scans. We averaged the standardized *z*-scores of 10 cognitive tests (Fawns-Ritchie & Deary, 2020) into four different cognitive domains: attention/psychomotor speed (reaction time, trail making - numeric path, symbol digit substitution tests; subsample of 11,531 men and 13,125 women), memory (numeric memory, paired associative learning, prospective memory, pairs matching tests; subsample of 11,657 men and 13,284 women), executive (fluid intelligence/reasoning, trail making - alphanumeric path tests; subsample of 11,535 men and 13,159 women), and visuospatial/visuoconstructional (matrix pattern completion, tower rearranging tests; subsample of 11,452 men and 13,013 women). More details about the cognitive tests are available at https://biobank.ndph.ox.ac.uk/showcase/label.cgi?id=100026.

### Connectivity Measures

We assessed the functional connectomes using four different connectivity measures. A network’s average connectivity (Conn-Ave) is defined as the average functional strength of all its connections. Similarly, we estimated the average positive connectivity (PosConn-Ave) and average negative connectivity (NegConn-Ave) as the mean strength of the network’s positive and negative connections, respectively. Finally, we calculated the number of negative connections NegConn-No in the network. These measures can be evaluated as:

$$\text{Conn}‐\text{Ave}=\frac{1}{\text{N}}\sum_{\text{i}\in \text{A}}{\text{w}}_{\text{i}},$$

$$\text{PosConn}‐\text{Ave}=\frac{1}{{\text{N}}_{\text{pos}}}\sum_{\text{i}\in {\text{A}}_{\text{pos}}}{\text{w}}_{\text{i}},$$

$$\text{NegConn}‐\text{Ave}=\frac{1}{{\text{N}}_{\text{neg}}}\sum_{\text{i}\in {\text{A}}_{\text{neg}}}{\text{w}}_{\text{i}},$$

where N is the total number of connections in the network A. The network A can be expressed as a sum of A_pos_ and A_neg_, which denote the networks consisting of only positive and negative connections. The total number of connections in A_pos_ and A_neg_ are denoted by N_pos_ and N_neg_, respectively.

The connectivity measures were calculated on the weighted connectivity networks consisting of positive and negative connections (shown in Figure 1A). For each weighted connectivity network, we calculated a corresponding binary network in which the individual connections retained their weight if they exceeded a certain threshold and were set to zero if they did not. In this process, the absolute value of each connection was compared to the threshold; however, their sign was preserved in the resulting binary matrix (i.e., negative connections in the weighted network remained negative in the binary network). As there are multiple thresholding approaches and there is currently no consensus as to which network density should be used (Fornito et al., 2013), we performed the thresholding at a density range of 6% to 33%, in steps of 1%. For densities below 6%, the networks became largely disconnected with fewer edges than nodes, whereas 33% was the maximum density that could be reached by all men’s and women’s networks of positive and negative connections (Supporting Information Figure S12).

After calculating all measures at each density within the complete density range, we evaluated the corresponding area under the curve (AUC) value, which was used to assess the between-sex differences (Supporting Information Figure S13). The AUC value was obtained by numerically integrating the measure values over the density range; this procedure resulted in a single numerical value for each network measure across the range of densities. As this analysis takes into account the entire density range, it is considered to be less susceptible to the thresholding process (Fornito et al., 2013).

### Topological Network Analysis

#### Single-layer network measures.

We split the weighted connectivity networks into networks consisting of positive connections and negative connections (Figure 1B). As network measures are not defined for negative weights, the weights in the network of negative connections were substituted by their absolute values. Therefore, the topological analysis was equivalent for both positive and negative connections networks; in the following we omit the subscript (pos/neg) for clarity. Both networks were independently binarized at the density range 6%–33%, and the AUC value for all measures was obtained across this range, as outlined above. The topology of these networks was evaluated by calculating the global efficiency (SL-Ge) and clustering coefficient (SL-CC).

#### Single-layer global efficiency.

In a weighted network, the distance d_ij_ between nodes i and i is the total sum of individual connection lengths along the shortest path that connects the two nodes. Because large weights often imply strong relationships and close proximity, connection lengths are inversely proportional to connection weights. While the shortest paths have the smallest weighted distance, this does not necessarily equate to having the fewest number of edges (Mijalkov et al., 2017). The regional global efficiency of a node i, denoted by SL-Ge_i_, is defined as the average inverse distance from to the other nodes in the network. The global efficiency of a network, SL-Ge, is determined as the average of the global efficiency of all nodes:

$$\text{SL}‐\text{Ge}=\frac{1}{\text{N}}\sum_{\text{i}\in \text{N}}{\text{SL}‐\text{Ge}}_{\text{i}}=\frac{1}{\text{N}}\sum_{\text{i}\in \text{N}}\frac{\sum_{\text{j}\in \text{N},\text{j}\neq \text{i}}{\text{d}}_{\text{i}\text{j}}^{-1}}{\text{n}-1}.$$

#### Single-layer clustering coefficient.

The clustering coefficient of a given node represents the fraction of the total number of triangles that are present around it. The network clustering coefficient is derived by averaging the clustering coefficients of all nodes. Using the definitions of the number of triangles and nodal degree, the network clustering coefficient can be represented as (Mijalkov et al., 2017):

$$\text{SL}‐\text{CC}=\frac{1}{\text{N}}\sum_{\text{i}\in \text{N}}{\text{SL}‐\text{CC}}_{\text{i}}=\frac{1}{\text{N}}\sum_{\text{i}\in \text{N}}\frac{2{\text{t}}_{\text{i}}}{{\text{k}}_{\text{i}}\left({\text{k}}_{\text{i}}-1\right)}.$$

k_i_, the degree of a node i, is defined as the total number of connections i has with other nodes in the network, regardless of their weight. If the neighbors of node i are connected with each other, a triangle can be constructed around i. In weighted networks, the total number of triangles around node i, t_i_, is calculated by summing the contributions of each individual triangle, defined as the geometric mean of the triangle’s edge weights:

$${\text{t}}_{\text{i}}=\frac{1}{2}\sum_{\text{j},\text{h}\in \text{N}}{\text{w}}_{\text{i}\text{j}}^{1/3}{\text{w}}_{\text{j}\text{k}}^{1/3}{\text{w}}_{\text{k}\text{i}}^{1/3}.$$

#### Multiplex network measures.

For each individual, we built a multiplex network with two layers, one with positive connections and the other with negative connections (Figure 1C). The multiplex networks were calculated at densities ranging from 6% to 33% by combining the two single-layer networks at the respective densities. At each density the multiplex networks can be represented by a supra-adjacency matrix, W, which consists of the intralayer adjacency matrices on the main diagonal and the interlayer connections in the off-diagonal entries. The interlayer connections are formed only among the node’s replicas, that is, ${\text{W}}_{i\in \alpha }^{j\in \beta }$ = 1 ⇔ *i* = *j*, where *α* and *β* denote layer 1 and layer 2, respectively. We evaluated the topology of these networks by calculating the participation and clustering coefficients and evaluating the AUC over the complete density range.

#### Multiplex participation coefficient.

This measure, MP-Pt, is used to quantify the heterogeneity in the connectivity patterns of a node across the different layers. It is calculated as:

$${\text{MP}‐\text{Pt}}_{\text{i}}=\frac{{\text{N}}_{\text{l}}}{{\text{N}}_{\text{l}}-1}\left[1-\sum_{\alpha =1}^{\text{M}}{\left(\frac{{\text{s}}_{\text{i}}^{\left[\alpha \right]}}{{\text{o}}_{\text{i}}}\right)}^{2}\right],$$

where N_l_ is the number of layers and ${\text{s}}_{\text{i}}^{\left[\alpha \right]}$ is the strength of node i at the *α*-th layer, defined as the sum of the weights of all edges connected to i. Finally, o_i_ = ∑_*α*_ ${\text{s}}_{\text{i}}^{\left[\alpha \right]}$ is the overlapping strength of the node i. MP-Pt_i_ determines whether i’s connections are evenly spread throughout the layers or are largely concentrated in one or a few layers. MP-Pt_i_ has values in the range 0 to 1 and, in general, bigger MP-Pt_i_ values suggest a more equal distribution of node i’s connections in all layers of the multiplex network. The global participation coefficient MP-Pt of the multiplex network is defined as the average of nodal MP-Pt_i_ coefficients over all nodes.

#### Multiplex clustering coefficient.

The clustering coefficient can be used to assess whether the clustering features of the aggregated multiplex network differed from those of the individual layers. In contrast to single-layer clustering, a triangle in a multiplex network is formed by one edge from one layer and the remaining two edges from the other layer. Therefore, the multiplex clustering coefficient of a node i (MP-CCi) is defined as:

$${\text{MP}‐\text{CC}}_{\text{i}}=\frac{\sum_{\alpha }\sum_{\beta \neq \alpha }\sum_{\text{i}\neq \text{m},\text{j}}{\left({\text{w}}_{\text{ij}}^{\left[\alpha \right]}{\text{w}}_{\text{jm}}^{\left[\beta \right]}{\text{w}}_{\text{mi}}^{\left[\alpha \right]}\right)}^{\frac{1}{3}}}{\left(\text{M}-1\right)\sum_{\alpha }{\text{k}}_{\text{i}}^{\left[\alpha \right]}\left({\text{k}}_{\text{i}}^{\left[\alpha \right]}-1\right)},$$

where ${\text{w}}_{\text{ij}}^{\left[\alpha \right]}$ denote the connection between the nodes i and j in the layer *α* and k_i_ denotes the degree of a node i. The global multiplex clustering coefficient is calculated as the average of nodal clustering coefficients across all nodes.

#### Multilayer network measures.

We constructed two-layer multilayer networks for each individual following an analogous procedure as the one of multiplex networks. However, in contrast to the multiplex approach, we established weighted interlayer connections between all node pairs to quantify the degree of relationship between the two layers. In this framework, the strength of the between-layer relationship is an independent variable that can be controlled by the weight of the interlayer connections. Due to the variability of the intralayer connection weights in all subjects, we define the interlayer weights, w_ij_, as a fraction of each participant’s biggest absolute functional connection:

$${\text{w}}_{\text{ij}}=\sigma *\max\left({\text{w}}_{\text{max}}^{\alpha }{\text{w}}_{\text{max}}^{\beta }\right),\;\forall \text{i}\in \alpha \;\text{and}\;\forall \text{j}\in \beta ,$$

where ${\text{w}}_{\text{max}}^{\alpha }$ and ${\text{w}}_{\text{max}}^{\beta }$ denote the maximum weight within layers 1 and 2 respectively, and *σ* is the fraction of the maximum weight. In our analyses, we have evaluated the multilayer in the *σ* range of 0.05 to 1, in steps of 0.05. Previous studies have explored the possibility of including interlayer connections with variable strengths in the case of magnetoencephalographic recordings (Buldú & Porter, 2018), which could open possibilities for future studies to extend these measures. In our study we fixed these strengths to have the same weight for an easier interpretation of our method and the corresponding results.

#### Multilayer global efficiency.

Consider a node i, which is part of layer 1. The multilayer global efficiency of this node, denoted by ML-Ge_i_, reflects the difference between the average inverse distances from i to all nodes in layer 2 and layer 1. Specifically, we first calculate the “same-layer” global efficiency, SameL-Ge_i_, using the methods defined in the section *Single-Layer Global Efficiency*, which considers only the shortest paths between i and all nodes belonging to layer 1. Note that the SameL-Ge_i_ does not depend on the interlayer weights as it considers only the shortest distances between nodes within the same layer:

$${\text{SameL}‐\text{Ge}}_{\text{i}}=\frac{\sum_{\text{j}\in \alpha }{\text{d}}_{\text{ij}}^{-1}}{\text{n}-1},$$

where n denotes the number of nodes and d_ij_ denotes the distance between the nodes i and j as before. In the second step, we calculate the “opposite-layer” global efficiency, OppL-Ge_i_, which assesses the average inverse distances from i to all nodes in layer 2. To this end, we build a new network of size n + 1, which includes i and all nodes and connections of layer 2. Then, OppL-Ge_i_ can be calculated as:

$${\text{OppL}‐\text{Ge}}_{\text{i}}\left(\sigma \right)=\frac{\sum_{\text{j}\in \beta }{\text{d}}_{\text{ij}}^{-1}}{\left(\text{n}+1\right)-1}.$$

We emphasize that OppL-Ge_i_(*σ*) has an explicit dependence on *σ* since node i is connected with the nodes in layer 2 via the interlayer connections of weight w_ij_, which depend explicitly on *σ*. Then, the multilayer global efficiency of node i, ML-Ge_i_, can be defined as:

$${\text{ML}‐\text{Ge}}_{\text{i}}\left(\sigma \right)={\text{OL}‐\text{Ge}}_{\text{i}}\left(\sigma \right)-{\text{SL}‐\text{Ge}}_{\text{i}}.$$

Therefore, ML-Ge_i_ measures the difference in global efficiency between the layer of negative and positive connections at different strengths of interlayer relation that depend on the value of *σ*. Higher values of ML-Ge_i_ suggest that the two layers have more divergent topology in terms of information integration. In this case, the interlayer weights have larger impact of the topology of the multilayer network, which leads to a stronger relationship between the layers.

#### Multilayer clustering coefficient.

We defined this measure, ML-CC_i_(*σ*), analogously to the multilayer global efficiency, in order to quantify the difference in the clustering properties between the two layers at different strengths of between-layer relationship. We first calculated SameL-CC_i_ and OppL-CC_i_(*σ*) by following the procedures outlined in the section *Single-Layer Clustering Coefficient*. Then, the ML-CC_i_(*σ*) was calculated as:

$${\text{ML}‐\text{CC}}_{\text{i}}\left(\sigma \right)={\text{OppL}‐\text{CC}}_{\text{i}}\left(\sigma \right)-{\text{SameL}‐\text{CC}}_{\text{i}},$$

and the network multilayer clustering coefficient was calculated as the average of ML-CC_i_(*σ*) over all nodes in the network.

#### Partial least squares regression analysis (PLS).

PLS performs a linear decomposition of the predictor and predicted variable matrix into latent variables (LVs) optimized so that the covariance between the resulting predictor and predicted matrix components (called factors and loadings, respectively) is maximal (Abdi & Williams, 2013). We fit a PLS regression model for each predicted variable (cognitive tests, structural measures, and prevalence of cardiovascular diseases) independently. The optimal component number for the decomposition was determined in each case by the Bayesian information criterion (BIC) based on the estimated degree of freedom of the PLS model (Krämer & Sugiyama, 2011). In particular, predictor and predicted variable matrices were decomposed into 2–12 LVs, with the optimal number of LVs based on BIC scores being defined as follows: attention = 5, executive = 6, memory = 2, thickness = 12, visuospatial = 6, volumes = 5, WMH = 5, heart attack = 3, hypertension = 4. The contribution of each variable to the prediction was quantified via variable importance in the projection (VIP) scores, calculated as the sum of PLS weights over LVs, weighted by the variance explained by each LV. Variables were defined as significant contributors to the prediction based on a VIP score of > 1 (Chong & Jun, 2005).

### Genetic Association Analyses

We examined the association between 9 million high-quality imputed common variants (MAF > 1%, imputation INFO score > 0.8 and Hardy–Weinberg equilibrium P > 10^−10^) from UK Biobank and each derived phenotype using linear mixed-effects models as implemented in REGENIE (Jamialahmadi et al., 2021; Mbatchou et al., 2021). All traits were rank-based inverse normal transformed before the analysis, and adjusted for age at MRI, sex, age^2^, age * sex, age^2^ * sex, body mass index (BMI), the first 10 principal components of ancestry, and genotyping array. For step 1, we used a subset of high-quality directly genotyped variants as described before (Jamialahmadi et al., 2021). We next performed a stringent physical LD clumping (PLINK parameters: –clump-p1 5e-8 –clump-r2 0.05 –clump-kb 1000, after excluding individuals with third-degree or closer relatives) (Bycroft et al., 2018; Chang et al., 2015), followed by an approximate step-wise model selection in conditional and joint multiple-SNP analysis (COJO-GCTA) (Yang et al., 2012), with a window of 1 Mb and using 50,000 randomly selected unrelated Europeans (in-sample LD structure) as described before (Jamialahmadi et al., 2021).

### Colocalization

Colocalization was performed between independent genetic loci from COJO-GCTA and summary statistics of gene expression quantitative trait loci (eQTL) of 49 tissues in GTEx v8, BrainSeq, ROSMAP, Braineac2 and CommonMind from eQTL cataolgue release 4 (GTEx Consortium, 2020; Guelfi et al., 2020; Jaffe et al., 2018; Kerimov et al., 2021; Ng et al., 2017; Sieberts et al., 2020). All genes with at least one significant association (FDR adjusted *p* value < 0.1) within a window of 1 Mb around each index variant were tested using coloc R package (Giambartolomei et al., 2014) with default priors, and H4 posterior probability (PP) > 0.8 was considered as a strong evidence that both traits share the same causal variant.

### Statistical Analysis

The statistical significance of the differences between men and women was assessed by performing nonparametric permutation tests with 10,000 permutations, which were considered significant for a two-tailed test of the null hypothesis at *p* < 0.05. These results were adjusted for multiple comparisons by applying false discovery rate (FDR) corrections at *q* < 0.05 using the Benjamini–Hochberg procedure (Benjamini & Hochberg, 1995). All results remained statistically significant after adjusting for average cortical thickness and subcortical volumes, suggesting that the observed between-sex differences were independent of brain atrophy (data not shown). To detect sex differences over aging, we used linear regression models where all functional connectivity measures for men and women at each age were summarized by their average value due to the high overlap between brain measures across different ages. Then, they were included as the dependent variables in the linear regression models, whereas age, sex, age^2^, age * sex, and age^2^ * sex were included as independent variables. The best model for each measure (Supporting Information: Linear Models) consisted of a combination of predictors that resulted in the minimum value of the Akaike information criterion (AIC) for that model. The significance of the overall model and the independent coefficients was evaluated by an *F* test, which was considered significant at *p* < 0.05. To compare the performance of the different models, we used the AIC and the mean squared error of all models.

### Data Availability

The authors did not participate in data collection. The primary data source used in the study was the UK Biobank, which requires an application for access (https://www.ukbiobank.ac.uk/). This study was conducted under application number 37142. All data from eQTL Catalogue project are freely available at https://www.ebi.ac.uk/eqtl/.

### Ethics Declarations

“UK Biobank has approval from the North West Multi-centre Research Ethics Committee (MREC) as a Research Tissue Bank (RTB) approval.” (from https://www.ukbiobank.ac.uk/learn-more-about-uk-biobank/about-us/ethics). Informed consent was obtained from all UK Biobank participants. The current study is covered by this approval as we did not use any additional data or re-contacted the participants.

## ACKNOWLEDGMENTS

We thank the following: Swedish Research Council; Swedish Alzheimer Foundation; Swedish Brain Foundation; Strategic Research Area Neuroscience (StratNeuro); Center for Medical Innovation (CIMED); Foundation for Geriatric Diseases at Karolinska Institutet; Gamla Tjänarinnor; Stohnes Foundation; Lars Hierta Memorial Foundation.

## SUPPORTING INFORMATION

Supporting information for this article is available at https://doi.org/10.1162/netn_a_00286.

## AUTHOR CONTRIBUTIONS

Mite Mijalkov: Conceptualization; Formal analysis; Funding acquisition; Methodology; Visualization; Writing – original draft; Writing – review & editing. Dániel Veréb: Formal analysis; Methodology; Writing – original draft; Writing – review & editing. Oveis Jamialahmadi: Formal analysis; Writing – original draft; Writing – review & editing. Anna Canal-Garcia: Software; Visualization; Writing – review & editing. Emiliano Gómez-Ruiz: Software; Writing – review & editing. Didac Vidal-Piñeiro: Writing – review & editing. Stefano Romeo: Formal analysis; Writing – original draft; Writing – review & editing. Giovanni Volpe: Conceptualization; Methodology; Supervision; Writing – original draft; Writing – review & editing. Joana Pereira: Conceptualization; Funding acquisition; Methodology; Supervision; Writing – original draft; Writing – review & editing.

## FUNDING INFORMATION

Joana Pereira, Vetenskapsrådet (https://dx.doi.org/10.13039/501100004359). Joana Pereira, Alzheimerfonden (https://dx.doi.org/10.13039/501100008599). Joana Pereira, Hjärnfonden (https://dx.doi.org/10.13039/501100003792). Joana Pereira, Strategic Research Area Neuroscience (StratNeuro). Joana Pereira, Center for Medical Innovation (CIMED). Mite Mijalkov, Foundation for Geriatric Diseases at Karolinska Institutet. Mite Mijalkov, Stiftelsen för Gamla Tjänarinnor (https://dx.doi.org/10.13039/100010815). Mite Mijalkov, Gun och Bertil Stohnes Stiftelse (https://dx.doi.org/10.13039/100009673). Mite Mijalkov, Stiftelsen Lars Hiertas Minne (https://dx.doi.org/10.13039/501100004722).

## TECHNICAL TERMS

Negative connections: Anticorrelations (negative associations) between the nodes in the whole-brain connectome.
Positive connections: Correlations (positive associations) between the nodes in the whole- brain connectome.
Connectivity measures: Measures summarizing the average functional connectivity strength of the whole-brain connectome.
Multiplex network: A two-layer multilayer network where interlayer connections are allowed only between corresponding nodes in the positive and negative layers.
Multilayer network: A two-layer network where interlayer connections are allowed between all pairs of nodes in the two layers.
*σ*: The fraction of the strongest functional connection in the corresponding multilayer network that is used to calculate the weight of the interlayer connections.
Multilayer global efficiency: Measure that assesses the level of functional integration in a multilayer network.
Multilayer clustering coefficient: Measure that assesses the level of functional segregation in a multilayer network.
Single-layer measures: Classical network measures of clustering coefficient and global efficiency calculated for the networks of positive and negative connections separately.
Partial least squares (PLS) regression: A multivariate regression analysis that projects sets of dependent and explanatory variables to a lower dimensional subspace consisting of latent variables that are maximally correlated.

## References

[bib1] Abdi, H., & Williams, L. J. (2013). Partial least squares methods: Partial least squares correlation and partial least square regression. In Computational toxicology (pp. 549–579). Springer. 10.1007/978-1-62703-059-5_23, 23086857

[bib2] Alfaro-Almagro, F., Jenkinson, M., Bangerter, N. K., Andersson, J. L. R., Griffanti, L., Douaud, G., … Smith, S. M. (2018). Image processing and quality control for the first 10,000 brain imaging datasets from UK Biobank. NeuroImage, 166, 400–424. 10.1016/j.neuroimage.2017.10.034, 29079522 PMC5770339

[bib3] Allen, E. A., Erhardt, E. B., Damaraju, E., Gruner, W., Segall, J. M., Silva, R. F., … Calhoun, V. D. (2011). A baseline for the multivariate comparison of resting-state networks. Frontiers in Systems Neuroscience, 5, 2. 10.3389/fnsys.2011.00002, 21442040 PMC3051178

[bib4] Anand, S. S., Islam, S., Rosengren, A., Franzosi, M. G., Steyn, K., Yusufali, A. H., … Yusuf, S. (2008). Risk factors for myocardial infarction in women and men: Insights from the interheart study. European Heart Journal, 29(7), 932–940. 10.1093/eurheartj/ehn018, 18334475

[bib5] Ancoli-Israel, S. (2009). Sleep and its disorders in aging populations. Sleep Medicine, 10, S7–S11. 10.1016/j.sleep.2009.07.004, 19647483

[bib6] Barber, A. D., Caffo, B. S., Pekar, J. J., & Mostofsky, S. H. (2013). Developmental changes in within-and between-network connectivity between late childhood and adulthood. Neuropsychologia, 51(1), 156–167. 10.1016/j.neuropsychologia.2012.11.011, 23174403 PMC3543510

[bib7] Battiston, F., Nicosia, V., & Latora, V. (2014). Structural measures for multiplex networks. Physical Review E, 89(3), 032804. 10.1103/PhysRevE.89.032804, 24730896

[bib8] Benjamini, Y., & Hochberg, Y. (1995). Controlling the false discovery rate: A practical and powerful approach to multiple testing. Journal of the Royal Statistical Society: Series B (Methodological), 57(1), 289–300. 10.1111/j.2517-6161.1995.tb02031.x

[bib9] Betzel, R. F., Byrge, L., He, Y., Goñi, J., Zuo, X.-N., & Sporns, O. (2014). Changes in structural and functional connectivity among resting-state networks across the human lifespan. NeuroImage, 102, 345–357. 10.1016/j.neuroimage.2014.07.067, 25109530

[bib10] Biswal, B., Zerrin Yetkin, F., Haughton, V. M., & Hyde, J. S. (1995). Functional connectivity in the motor cortex of resting human brain using echo-planar MRI. Magnetic Resonance in Medicine, 34(4), 537–541. 10.1002/mrm.1910340409, 8524021

[bib11] Biswal, B. B., Mennes, M., Zuo, X.-N., Gohel, S., Kelly, C., Smith, S. M., … Milham, M. P. (2010). Toward discovery science of human brain function. Proceedings of the National Academy of Sciences, 107(10), 4734–4739. 10.1073/pnas.0911855107, 20176931 PMC2842060

[bib12] Buldú, J. M., & Porter, M. A. (2018). Frequency-based brain networks: From a multiplex framework to a full multilayer description. Network Neuroscience, 2(4), 418–441. 10.1162/netn_a_00033, 30294706 PMC6147638

[bib13] Bullmore, E., & Sporns, O. (2012). The economy of brain network organization. Nature Reviews Neuroscience, 13(5), 336–349. 10.1038/nrn3214, 22498897

[bib14] Bycroft, C., Freeman, C., Petkova, D., Band, G., Elliott, L. T., Sharp, K., … Marchini, J. (2018). The UK Biobank resource with deep phenotyping and genomic data. Nature, 562(7726), 203–209. 10.1038/s41586-018-0579-z, 30305743 PMC6786975

[bib15] Cabeza, R., Anderson, N. D., Locantore, J. K., & McIntosh, A. R. (2002). Aging gracefully: Compensatory brain activity in high-performing older adults. NeuroImage, 17(3), 1394–1402. 10.1006/nimg.2002.1280, 12414279

[bib16] Chai, X. J., Castañón, A. N., Öngür, D., & Whitfield-Gabrieli, S. (2012). Anticorrelations in resting state networks without global signal regression. NeuroImage, 59(2), 1420–1428. 10.1016/j.neuroimage.2011.08.048, 21889994 PMC3230748

[bib17] Chai, X. J., Ofen, N., Gabrieli, J. D. E., & Whitfield-Gabrieli, S. (2014). Selective development of anticorrelated networks in the intrinsic functional organization of the human brain. Journal of Cognitive Neuroscience, 26(3), 501–513. 10.1162/jocn_a_00517, 24188367 PMC4175987

[bib18] Chan, M. Y., Park, D. C., Savalia, N. K., Petersen, S. E., & Wig, G. S. (2014). Decreased segregation of brain systems across the healthy adult lifespan. Proceedings of the National Academy of Sciences, 111(46), E4997–E5006. 10.1073/pnas.1415122111, 25368199 PMC4246293

[bib19] Chang, C. C., Chow, C. C., Tellier, L. C., Vattikuti, S., Purcell, S. M., & Lee, J. J. (2015). Second-generation PLINK: Rising to the challenge of larger and richer datasets. Gigascience, 4(1), s13742-015-0047-8. 10.1186/s13742-015-0047-8, 25722852 PMC4342193

[bib20] Chekroud, A. M., Ward, E. J., Rosenberg, M. D., & Holmes, A. J. (2016). Patterns in the human brain mosaic discriminate males from females. Proceedings of the National Academy of Sciences, 113(14), E1968. 10.1073/pnas.1523888113, 26984491 PMC4833246

[bib21] Chen, Y.-C., Jiao, Y., Cui, Y., Shang, S.-A., Ding, J., Feng, Y., … Teng, G.-J. (2014). Aberrant brain functional connectivity related to insulin resistance in type 2 diabetes: A resting-state fMRI study. Diabetes Care, 37(6), 1689–1696. 10.2337/dc13-2127, 24658392

[bib22] Chong, I.-G., & Jun, C.-H. (2005). Performance of some variable selection methods when multicollinearity is present. Chemometrics and Intelligent Laboratory Systems, 78(1–2), 103–112. 10.1016/j.chemolab.2004.12.011

[bib23] Cicchetti, D. V., & Sparrow, S. A. (1981). Developing criteria for establishing interrater reliability of specific items: Applications to assessment of adaptive behavior. American Journal of Mental Deficiency, 86(2), 127–137. 7315877

[bib24] Cosgrove, K. P., Mazure, C. M., & Staley, J. K. (2007). Evolving knowledge of sex differences in brain structure, function, and chemistry. Biological Psychiatry, 62(8), 847–855. 10.1016/j.biopsych.2007.03.001, 17544382 PMC2711771

[bib25] Crimmins, E. M. (2015). Lifespan and healthspan: Past, present, and promise. The Gerontologist, 55(6), 901–911. 10.1093/geront/gnv130, 26561272 PMC4861644

[bib26] Damoiseaux, J. S. (2017). Effects of aging on functional and structural brain connectivity. NeuroImage, 160, 32–40. 10.1016/j.neuroimage.2017.01.077, 28159687

[bib27] Deary, I. J., Corley, J., Gow, A. J., Harris, S. E., Houlihan, L. M., Marioni, R. E., … Starr, J. M. (2009). Age-associated cognitive decline. British Medical Bulletin, 92(1), 135–152. 10.1093/bmb/ldp033, 19776035

[bib28] Desikan, R. S., Ségonne, F., Fischl, B., Quinn, B. T., Dickerson, B. C., Blacker, D., … Killiany, R. J. (2006). An automated labeling system for subdividing the human cerebral cortex on MRI scans into gyral based regions of interest. NeuroImage, 31(3), 968–980. 10.1016/j.neuroimage.2006.01.021, 16530430

[bib29] Dhingra, R., & Vasan, R. S. (2012). Age as a risk factor. Medical Clinics, 96(1), 87–91. 10.1016/j.mcna.2011.11.003, 22391253 PMC3297980

[bib30] Elliott, L. T., Sharp, K., Alfaro-Almagro, F., Shi, S., Miller, K. L., Douaud, G., … Smith, S. M. (2018). Genome-wide association studies of brain imaging phenotypes in UK Biobank. Nature, 562(7726), 210–216. 10.1038/s41586-018-0571-7, 30305740 PMC6786974

[bib31] Fawns-Ritchie, C., & Deary, I. J. (2020). Reliability and validity of the UK Biobank cognitive tests. PLoS One, 15(4), e0231627. 10.1371/journal.pone.0231627, 32310977 PMC7170235

[bib32] Ferreira, L. K., Regina, A. C. B., Kovacevic, N., Martin, M. d. G. M., Santos, P. P., Carneiro, C. d. G., … Busatto, G. F. (2016). Aging effects on whole-brain functional connectivity in adults free of cognitive and psychiatric disorders. Cerebral Cortex, 26(9), 3851–3865. 10.1093/cercor/bhv190, 26315689

[bib33] Ferretti, M. T., Iulita, M. F., Cavedo, E., Chiesa, P. A., Dimech, A. S., Chadha, A. S., … Women’s Brain Project and the Alzheimer Precision Medicine Initiative. (2018). Sex differences in Alzheimer disease—The gateway to precision medicine. Nature Reviews Neurology, 14(8), 457–469. 10.1038/s41582-018-0032-9, 29985474

[bib34] Filippi, M., Valsasina, P., Misci, P., Falini, A., Comi, G., & Rocca, M. A. (2013). The organization of intrinsic brain activity differs between genders: A resting-state fMRI study in a large cohort of young healthy subjects. Human Brain Mapping, 34(6), 1330–1343. 10.1002/hbm.21514, 22359372 PMC6870508

[bib35] Foo, H., Thalamuthu, A., Jiang, J., Koch, F. C., Mather, K. A., Wen, W., & Sachdev, P. S. (2021a). Age- and sex-related topological organization of human brain functional networks and their relationship to cognition. Frontiers in Aging Neuroscience, 13, 758817. 10.3389/fnagi.2021.758817, 34975453 PMC8718995

[bib36] Foo, H., Thalamuthu, A., Jiang, J., Koch, F. C., Mather, K. A., Wen, W., & Sachdev, P. S. (2021b). Novel genetic variants associated with brain functional networks in 18,445 adults from the UK Biobank. Scientific Reports, 11(1), 14633. 10.1038/s41598-021-94182-9, 34272439 PMC8285376

[bib37] Fornito, A., Zalesky, A., & Breakspear, M. (2013). Graph analysis of the human connectome: Promise, progress, and pitfalls. NeuroImage, 80, 426–444. 10.1016/j.neuroimage.2013.04.087, 23643999

[bib38] Fox, M. D., Snyder, A. Z., Vincent, J. L., Corbetta, M., Van Essen, D. C., & Raichle, M. E. (2005). The human brain is intrinsically organized into dynamic, anticorrelated functional networks. Proceedings of the National Academy of Sciences, 102(27), 9673–9678. 10.1073/pnas.0504136102, 15976020 PMC1157105

[bib39] Fox, M. D., Zhang, D., Snyder, A. Z., & Raichle, M. E. (2009). The global signal and observed anticorrelated resting state brain networks. Journal of Neurophysiology, 101(6), 3270–3283. 10.1152/jn.90777.2008, 19339462 PMC2694109

[bib40] Geerligs, L., Renken, R. J., Saliasi, E., Maurits, N. M., & Lorist, M. M. (2015). A brain-wide study of age-related changes in functional connectivity. Cerebral Cortex, 25(7), 1987–1999. 10.1093/cercor/bhu012, 24532319

[bib41] Giambartolomei, C., Vukcevic, D., Schadt, E. E., Franke, L., Hingorani, A. D., Wallace, C., & Plagnol, V. (2014). Bayesian test for colocalisation between pairs of genetic association studies using summary statistics. PLoS Genetics, 10(5), e1004383. 10.1371/journal.pgen.1004383, 24830394 PMC4022491

[bib42] Gillis, E. E., & Sullivan, J. C. (2016). Sex differences in hypertension: Recent advances. Hypertension, 68(6), 1322–1327. 10.1161/HYPERTENSIONAHA.116.06602, 27777357 PMC5159215

[bib43] Goelman, G., Gordon, N., & Bonne, O. (2014). Maximizing negative correlations in resting-state functional connectivity MRI by time-lag. PLoS One, 9(11), e111554. 10.1371/journal.pone.0111554, 25396416 PMC4232255

[bib44] Golden, L. C., & Voskuhl, R. (2017). The importance of studying sex differences in disease: The example of multiple sclerosis. Journal of Neuroscience Research, 95(1–2), 633–643. 10.1002/jnr.23955, 27870415 PMC5825192

[bib45] Goldstone, A., Mayhew, S. D., Przezdzik, I., Wilson, R. S., Hale, J. R., & Bagshaw, A. P. (2016). Gender specific re-organization of resting-state networks in older age. Frontiers in Aging Neuroscience, 8, 285. 10.3389/fnagi.2016.00285, 27932978 PMC5122714

[bib46] Griffa, A., Baumann, P. S., Thiran, J.-P., & Hagmann, P. (2013). Structural connectomics in brain diseases. NeuroImage, 80, 515–526. 10.1016/j.neuroimage.2013.04.056, 23623973

[bib47] Griffanti, L., Zamboni, G., Khan, A., Li, L., Bonifacio, G., Sundaresan, V., … Jenkinson, M. (2016). BIANCA (Brain Intensity AbNormality Classification Algorithm): A new tool for automated segmentation of white matter hyperintensities. NeuroImage, 141, 191–205. 10.1016/j.neuroimage.2016.07.018, 27402600 PMC5035138

[bib48] GTEx Consortium. (2020). The GTEx Consortium atlas of genetic regulatory effects across human tissues. Science, 369(6509), 1318–1330. 10.1126/science.aaz1776, 32913098 PMC7737656

[bib49] Guelfi, S., D’Sa, K., Botía, J. A., Vandrovcova, J., Reynolds, R. H., Zhang, D., … Ryten, M. (2020). Regulatory sites for splicing in human basal ganglia are enriched for disease-relevant information. Nature Communications, 11(1), 1041. 10.1038/s41467-020-14483-x, 32098967 PMC7042265

[bib50] Hampson, M., Driesen, N., Roth, J. K., Gore, J. C., & Constable, R. T. (2010). Functional connectivity between task-positive and task-negative brain areas and its relation to working memory performance. Magnetic Resonance Imaging, 28(8), 1051–1057. 10.1016/j.mri.2010.03.021, 20409665 PMC2936669

[bib51] Hou, Y., Dan, X., Babbar, M., Wei, Y., Hasselbalch, S. G., Croteau, D. L., & Bohr, V. A. (2019). Ageing as a risk factor for neurodegenerative disease. Nature Reviews Neurology, 15(10), 565–581. 10.1038/s41582-019-0244-7, 31501588

[bib52] Jaffe, A. E., Straub, R. E., Shin, J. H., Tao, R., Gao, Y., Collado-Torres, L., … Weinberger, D. R. (2018). Developmental and genetic regulation of the human cortex transcriptome illuminate schizophrenia pathogenesis. Nature Neuroscience, 21(8), 1117–1125. 10.1038/s41593-018-0197-y, 30050107 PMC6438700

[bib53] Jamialahmadi, O., Mancina, R. M., Ciociola, E., Tavaglione, F., Luukkonen, P. K., Baselli, G., … Romeo, S. (2021). Exome-wide association study on alanine aminotransferase identifies sequence variants in the GPAM and APOE associated with fatty liver disease. Gastroenterology, 160(5), 1634–1646. 10.1053/j.gastro.2020.12.023, 33347879

[bib54] Joel, D., & Fausto-Sterling, A. (2016). Beyond sex differences: New approaches for thinking about variation in brain structure and function. Philosophical Transactions of the Royal Society B: Biological Sciences, 371(1688), 20150451. 10.1098/rstb.2015.0451, 26833844 PMC4785909

[bib55] Jones, S. E., van Hees, V. T., Mazzotti, D. R., Marques-Vidal, P., Sabia, S., van der Spek, A., … Wood, A. R. (2019). Genetic studies of accelerometer-based sleep measures yield new insights into human sleep behaviour. Nature Communications, 10(1), 1585. 10.1038/s41467-019-09576-1, 30952852 PMC6451011

[bib56] Keller, J. B., Hedden, T., Thompson, T. W., Anteraper, S. A., Gabrieli, J. D., & Whitfield-Gabrieli, S. (2015). Resting-state anticorrelations between medial and lateral prefrontal cortex: Association with working memory, aging, and individual differences. Cortex, 64, 271–280. 10.1016/j.cortex.2014.12.001, 25562175 PMC4346444

[bib57] Kerimov, N., Hayhurst, J. D., Peikova, K., Manning, J. R., Walter, P., Kolberg, L., … Alasoo, K. (2021). A compendium of uniformly processed human gene expression and splicing quantitative trait loci. Nature Genetics, 53(9), 1290–1299. 10.1038/s41588-021-00924-w, 34493866 PMC8423625

[bib58] Klein, S. L., Neilson, K. M., Orban, J., Yaklichkin, S., Hoffbauer, J., Mood, K., … Moody, S. A. (2013). Conserved structural domains in FoxD4L1, a neural forkhead box transcription factor, are required to repress or activate target genes. PLoS One, 8(4), e61845. 10.1371/journal.pone.0061845, 23610594 PMC3627651

[bib59] Krämer, N., & Sugiyama, M. (2011). The degrees of freedom of partial least squares regression. Journal of the American Statistical Association, 106(494), 697–705. 10.1198/jasa.2011.tm10107

[bib60] Li, L., Pan, Z., & Yang, X. (2019). Key genes and co-expression network analysis in the livers of type 2 diabetes patients. Journal of Diabetes Investigation, 10(4), 951–962. 10.1111/jdi.12998, 30592156 PMC6626963

[bib61] Liu, S., Wang, Y.-S., Zhang, Q., Zhou, Q., Cao, L.-Z., Jiang, C., … Chinese Color Nest Consortium. (2021). Chinese Color Nest Project: An accelerated longitudinal brain-mind cohort. Developmental Cognitive Neuroscience, 52, 101020. 10.1016/j.dcn.2021.101020, 34653938 PMC8517840

[bib62] Lord, L.-D., Stevner, A. B., Deco, G., & Kringelbach, M. L. (2017). Understanding principles of integration and segregation using whole-brain computational connectomics: implications for neuropsychiatric disorders. Philosophical Transactions of the Royal Society A: Mathematical, Physical, and Engineering Sciences, 375(2096), 20160283. 10.1098/rsta.2016.0283, 28507228 PMC5434074

[bib63] Marek, S., Tervo-Clemmens, B., Calabro, F. J., Montez, D. F., Kay, B. P., Hatoum, A. S., … Dosenbach, N. U. F. (2022). Reproducible brain-wide association studies require thousands of individuals. Nature, 603(7902), 654–660. 10.1038/s41586-022-04492-9, 35296861 PMC8991999

[bib64] Masuda, N., Sakaki, M., Ezaki, T., & Watanabe, T. (2018). Clustering coefficients for correlation networks. Frontiers in Neuroinformatics, 12, 7. 10.3389/fninf.2018.00007, 29599714 PMC5863042

[bib65] Mazure, C. M., & Swendsen, J. (2016). Sex differences in Alzheimer’s disease and other dementias. The Lancet Neurology, 15(5), 451–452. 10.1016/S1474-4422(16)00067-3, 26987699 PMC4864429

[bib66] Mbatchou, J., Barnard, L., Backman, J., Marcketta, A., Kosmicki, J. A., Ziyatdinov, A., … Marchini, J. (2021). Computationally efficient whole-genome regression for quantitative and binary traits. Nature Genetics, 53(7), 1097–1103. 10.1038/s41588-021-00870-7, 34017140

[bib67] McCarrey, A. C., An, Y., Kitner-Triolo, M. H., Ferrucci, L., & Resnick, S. M. (2016). Sex differences in cognitive trajectories in clinically normal older adults. Psychology and Aging, 31(2), 166–175. 10.1037/pag0000070, 26796792 PMC4783196

[bib68] McGraw, K. O., & Wong, S. P. (1996). Forming inferences about some intraclass correlation coefficients. Psychological Methods, 1(1), 30–46. 10.1037/1082-989X.1.1.30

[bib69] Mijalkov, M., Kakaei, E., Pereira, J. B., Westman, E., Volpe, G., & Alzheimer’s Disease Neuroimaging Initiative. (2017). BRAPH: A graph theory software for the analysis of brain connectivity. PLoS One, 12(8), e0178798. 10.1371/journal.pone.0178798, 28763447 PMC5538719

[bib70] Mijalkov, M., Volpe, G., & Pereira, J. B. (2022). Directed brain connectivity identifies widespread functional network abnormalities in Parkinson’s disease. Cerebral Cortex, 32(3), 593–607. 10.1093/cercor/bhab237, 34331060 PMC8805861

[bib71] Miller, K. L., Alfaro-Almagro, F., Bangerter, N. K., Thomas, D. L., Yacoub, E., Xu, J., … Smith, S. M. (2016). Multimodal population brain imaging in the UK Biobank prospective epidemiological study. Nature Neuroscience, 19(11), 1523–1536. 10.1038/nn.4393, 27643430 PMC5086094

[bib72] Neitzel, J., Malik, R., Muetzel, R., Knol, M. J., Zonneveld, H., Georgakis, M. K., … Ewers, M. (2021). Genetic variants link lower segregation of brain networks to higher blood pressure and worse cognition within the general aging population. medRxiv. 10.1101/2021.08.12.21261975

[bib73] Ng, B., White, C. C., Klein, H.-U., Sieberts, S. K., McCabe, C., Patrick, E., … De Jager, P. L. (2017). An xQTL map integrates the genetic architecture of the human brain’s transcriptome and epigenome. Nature Neuroscience, 20(10), 1418–1426. 10.1038/nn.4632, 28869584 PMC5785926

[bib74] O’Brien, L. M., Ziegler, D. A., Deutsch, C. K., Frazier, J. A., Herbert, M. R., & Locascio, J. J. (2011). Statistical adjustments for brain size in volumetric neuroimaging studies: Some practical implications in methods. Psychiatry Research: Neuroimaging, 193(2), 113–122. 10.1016/j.pscychresns.2011.01.007, 21684724 PMC3510982

[bib75] Park, D. C., & Reuter-Lorenz, P. (2009). The adaptive brain: Aging and neurocognitive scaffolding. Annual Review of Psychology, 60, 173–196. 10.1146/annurev.psych.59.103006.093656, 19035823 PMC3359129

[bib76] Prata, D. P., Papagni, S. A., Mechelli, A., Fu, C. H. Y., Kambeitz, J., Picchioni, M., … McGuire, P. K. (2012). Effect of D-amino acid oxidase activator (DAOA; G72) on brain function during verbal fluency. Human Brain Mapping, 33(1), 143–153. 10.1002/hbm.21198, 21391259 PMC6870192

[bib77] Ramirez, L. A., & Sullivan, J. C. (2018). Sex differences in hypertension: Where we have been and where we are going. American Journal of Hypertension, 31(12), 1247–1254. 10.1093/ajh/hpy148, 30299518 PMC6233684

[bib78] Ritchie, S. J., Cox, S. R., Shen, X., Lombardo, M. V., Reus, L. M., Alloza, C., … Deary, I. J. (2018). Sex differences in the adult human brain: Evidence from 5216 UK Biobank participants. Cerebral Cortex, 28(8), 2959–2975. 10.1093/cercor/bhy109, 29771288 PMC6041980

[bib79] Ruigrok, A. N. V., Salimi-Khorshidi, G., Lai, M.-C., Baron-Cohen, S., Lombardo, M. V., Tait, R. J., & Suckling, J. (2014). A meta-analysis of sex differences in human brain structure. Neuroscience & Biobehavioral Reviews, 39, 34–50. 10.1016/j.neubiorev.2013.12.004, 24374381 PMC3969295

[bib80] Saberi, M., Khosrowabadi, R., Khatibi, A., Misic, B., & Jafari, G. (2021). Topological impact of negative links on the stability of resting-state brain network. Scientific Reports, 11(1), 2176. 10.1038/s41598-021-81767-7, 33500525 PMC7838299

[bib81] Sachdev, P. S., Parslow, R., Wen, W., Anstey, K., & Easteal, S. (2009). Sex differences in the causes and consequences of white matter hyperintensities. Neurobiology of Aging, 30(6), 946–956. 10.1016/j.neurobiolaging.2007.08.023, 17950492

[bib82] Sala-Llonch, R., Bartrés-Faz, D., & Junqué, C. (2015). Reorganization of brain networks in aging: A review of functional connectivity studies. Frontiers in Psychology, 6, 663. 10.3389/fpsyg.2015.00663, 26052298 PMC4439539

[bib83] Satterthwaite, T. D., Wolf, D. H., Roalf, D. R., Ruparel, K., Erus, G., Vandekar, S., … Gur, R. C. (2015). Linked sex differences in cognition and functional connectivity in youth. Cerebral Cortex, 25(9), 2383–2394. 10.1093/cercor/bhu036, 24646613 PMC4537416

[bib84] Scheinost, D., Finn, E. S., Tokoglu, F., Shen, X., Papademetris, X., Hampson, M., & Constable, R. T. (2015). Sex differences in normal age trajectories of functional brain networks. Human Brain Mapping, 36(4), 1524–1535. 10.1002/hbm.22720, 25523617 PMC5522589

[bib85] Sieberts, S. K., Perumal, T. M., Carrasquillo, M. M., Allen, M., Reddy, J. S., Hoffman, G. E., … Mangravite, L. M. (2020). Large eQTL meta-analysis reveals differing patterns between cerebral cortical and cerebellar brain regions. Scientific Data, 7(1), 340. 10.1038/s41597-020-00642-8, 33046718 PMC7550587

[bib86] Smith, K. M., & Dahodwala, N. (2014). Sex differences in Parkinson’s disease and other movement disorders. Experimental Neurology, 259, 44–56. 10.1016/j.expneurol.2014.03.010, 24681088

[bib87] Smith, S. M., Elliott, L. T., Alfaro-Almagro, F., McCarthy, P., Nichols, T. E., Douaud, G., & Miller, K. L. (2020). Brain aging comprises many modes of structural and functional change with distinct genetic and biophysical associations. Elife, 9, e52677. 10.7554/eLife.52677, 32134384 PMC7162660

[bib88] Spreng, R. N., Stevens, W. D., Viviano, J. D., & Schacter, D. L. (2016). Attenuated anticorrelation between the default and dorsal attention networks with aging: Evidence from task and rest. Neurobiology of Aging, 45, 149–160. 10.1016/j.neurobiolaging.2016.05.020, 27459935 PMC5003045

[bib89] Stumme, J., Jockwitz, C., Hoffstaedter, F., Amunts, K., & Caspers, S. (2020). Functional network reorganization in older adults: Graph-theoretical analyses of age, cognition and sex. NeuroImage, 214, 116756. 10.1016/j.neuroimage.2020.116756, 32201326

[bib90] Tagliazucchi, E., von Wegner, F., Morzelewski, A., Borisov, S., Jahnke, K., & Laufs, H. (2012). Automatic sleep staging using fMRI functional connectivity data. NeuroImage, 63(1), 63–72. 10.1016/j.neuroimage.2012.06.036, 22743197

[bib91] Tomasi, D., & Volkow, N. D. (2012a). Aging and functional brain networks. Molecular Psychiatry, 17(5), 549–558. 10.1038/mp.2011.81, 21727896 PMC3193908

[bib92] Tomasi, D., & Volkow, N. D. (2012b). Gender differences in brain functional connectivity density. Human Brain Mapping, 33(4), 849–860. 10.1002/hbm.21252, 21425398 PMC3250567

[bib93] van den Heuvel, M. P., & Pol, H. E. H. (2010). Exploring the brain network: A review on resting-state fMRI functional connectivity. European Neuropsychopharmacology, 20(8), 519–534. 10.1016/j.euroneuro.2010.03.008, 20471808

[bib94] Vidal-Piñeiro, D., Wang, Y., Krogsrud, S. K., Amlien, I. K., Baaré, W. F. C., Bartres-Faz, D., … Fjell, A. (2021). Individual variations in ‘brain age’ relate to early-life factors more than to longitudinal brain change. Elife, 10, e69995. 10.7554/eLife.69995, 34756163 PMC8580481

[bib95] Wang, Y., Xu, Q., Luo, J., Hu, M., & Zuo, C. (2019). Effects of age and sex on subcortical volumes. Frontiers in Aging Neuroscience, 11, 259. 10.3389/fnagi.2019.00259, 31616285 PMC6775221

[bib96] Weiss, E. M., Kemmler, G., Deisenhammer, E. A., Fleischhacker, W. W., & Delazer, M. (2003). Sex differences in cognitive functions. Personality and Individual Differences, 35(4), 863–875. 10.1016/S0191-8869(02)00288-X

[bib97] Wyss-Coray, T. (2016). Ageing, neurodegeneration and brain rejuvenation. Nature, 539(7628), 180–186. 10.1038/nature20411, 27830812 PMC5172605

[bib98] Xing, X.-X. (2021). Globally aging cortical spontaneous activity revealed by multiple metrics and frequency bands using resting-state functional MRI. Frontiers in Aging Neuroscience, 13, 803436. 10.3389/fnagi.2021.803436, 35027890 PMC8748263

[bib99] Yang, J., Ferreira, T., Morris, A. P., Medland, S. E., Madden, P. A. F., Heath, A. C., … Visscher, P. M. (2012). Conditional and joint multiple-SNP analysis of GWAS summary statistics identifies additional variants influencing complex traits. Nature Genetics, 44(4), 369–375. 10.1038/ng.2213, 22426310 PMC3593158

[bib100] Zalesky, A., Fornito, A., & Bullmore, E. (2012). On the use of correlation as a measure of network connectivity. NeuroImage, 60(4), 2096–2106. 10.1016/j.neuroimage.2012.02.001, 22343126

[bib101] Zhang, C., Cahill, N. D., Arbabshirani, M. R., White, T., Baum, S. A., & Michael, A. M. (2016). Sex and age effects of functional connectivity in early adulthood. Brain Connectivity, 6(9), 700–713. 10.1089/brain.2016.0429, 27527561 PMC5105352

[bib102] Zhang, C., Dougherty, C. C., Baum, S. A., White, T., & Michael, A. M. (2018). Functional connectivity predicts gender: Evidence for gender differences in resting brain connectivity. Human Brain Mapping, 39(4), 1765–1776. 10.1002/hbm.23950, 29322586 PMC6866578

