# Supplementary material for: Sex differences in multilayer functional network topology over the course of aging in 37543 UK Biobank participants
Source: Netw Neurosci. 2023 Jan 1;7(1):351–76. doi: 10.1162/netn_a_00286 (PMC10275214; doi:10.1162/netn_a_00286)
Supplement: Supplementary file 1 [file netn-7-1-351-s001.pdf]

Mijalkov, M., Veréb, D., Jamialahmadi, O., Canal-Garcia, A., Gómez-Ruiz, E., Vidal-Piñeiro, D., Romeo, S., Volpe, G. & Pereira, J. (2023). Supporting information for "Sex differences in multilayer functional network topology over the course of aging in 37543 UK Biobank participants. *Network Neuroscience*, *7*(1), 351–376. https://doi.org/10.1162/netn_a_00286

Sex differences in multilayer functional network topology over the course of aging in 37543 UK Biobank participants

Supplementary information

Figures

Mite Mijalkov,[1,*] Dániel Veréb,[1] Anna Canal Garcia,[1] Emiliano Gomez Ruiz,[2] Oveis Jamialahmadi,[3] Stefano Romeo,[3] Giovanni Volpe,[2] and Joana B. Pereira[1,4,*]

[1]*Department of Neurobiology, Care Sciences and Society, Karolinska Institutet, Stockholm, Sweden*

[2]*Department of Physics, Goteborg University, Goteborg, Sweden*

[3]*Department of Molecular and Clinical Medicine, Goteborg University, Goteborg, Sweden*

[4]*Memory Research Unit, Department of Clinical Sciences Malmö, Lund University, Lund, Sweden*

[*] Corresponding authors: Email: mite.mijalkov@ki.se // joana.pereira@ki.se. Address: KI, Dept. NVS, division of clinical geriatrics, Neo 7th floor, Blickagången 16, 141 83 Huddinge, Sweden.

I. SAMPLE CHARACTERISTICS

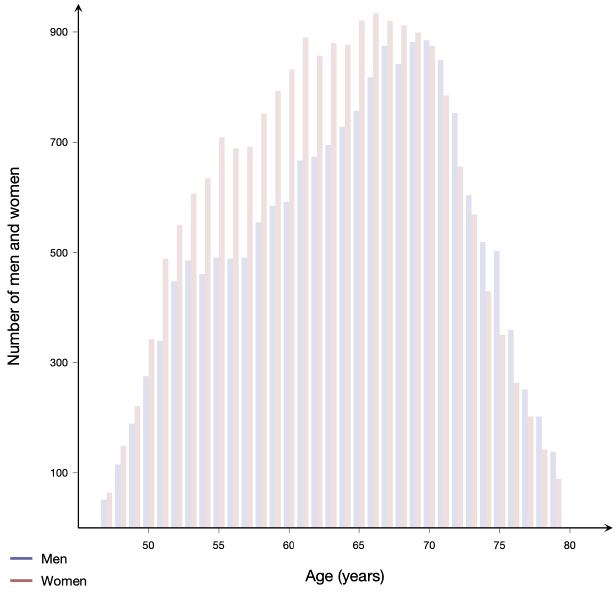

FIG. S1. **Sample characteristics.** Number of men (blue bars) and women (red bars) in the age range 47 - 79 years that were included in the current study.

A. Difference between men and women in different parameters.

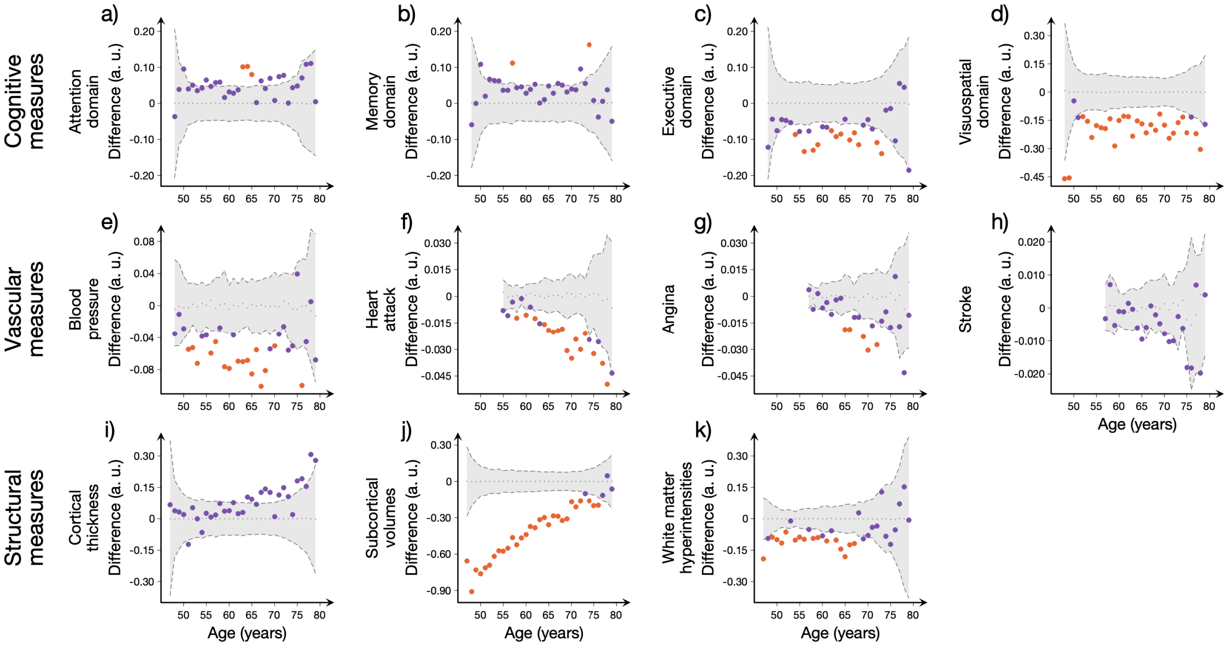

FIG. S2. **Differences between men and women in cognition, vascular and structural measures.** Plots showing the differences between men and women (calculated as women — men) in the a) attention, b) memory, c) executive and d) visuospatial cogntive domains. Differences between the prevalence of e) high blood pressure, f) heart attack, g) angina and h) stroke in men and women are also shown. Figure i-k show the observed sex differences in average cortical thickness, average subcortical volumes and white matter hyperintensities. The areas show the upper and lower bounds of the 95% confidence intervals (CI), and the differences in the corresponding measures between groups in blue circles as a function of individual's age. The differences are considered statistically significant if they fall outside the CIs. In particular, the orange circles show the differences that remained significant after applying a correction for multiple comparisons across the different age groups (FDR at $q < 0.05$).

II. CONNECTOGRAMS FOR MEN AND WOMEN AT DIFFERENT AGES.

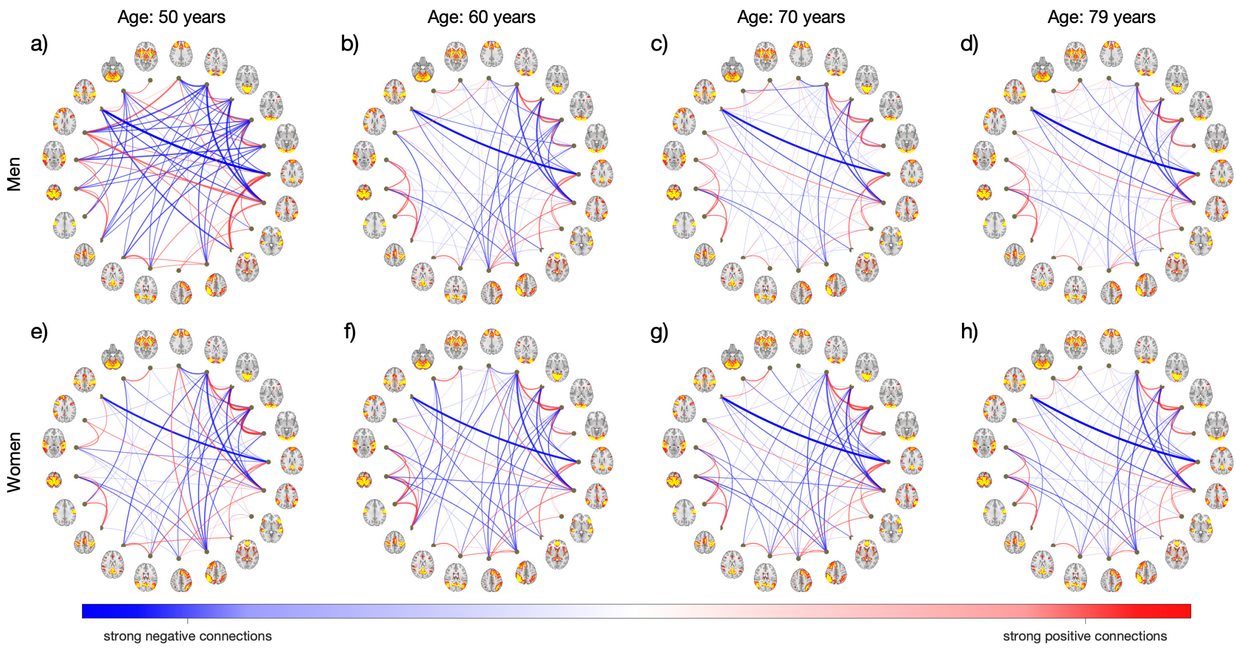

FIG. S3. **Connectogram-based representation of men's and women's average functional connectome at representative ages.** A representation of the men's and women's average functional connectivity networks at age of a-e) 50 years, b-f) 60 years, c-g) 70 years and d-h) 79 years of age. Thicker connections represent stronger functional connections; the positive and negative connections are shown in red and blue respectively. The 21 networks defined by group-ICA procedure represent the network nodes.

III. DIFFERENCES BETWEEN MEN AND WOMEN IN CONNECTIVITY, SINGLE LAYER AND MULTILAYER MEASURES.

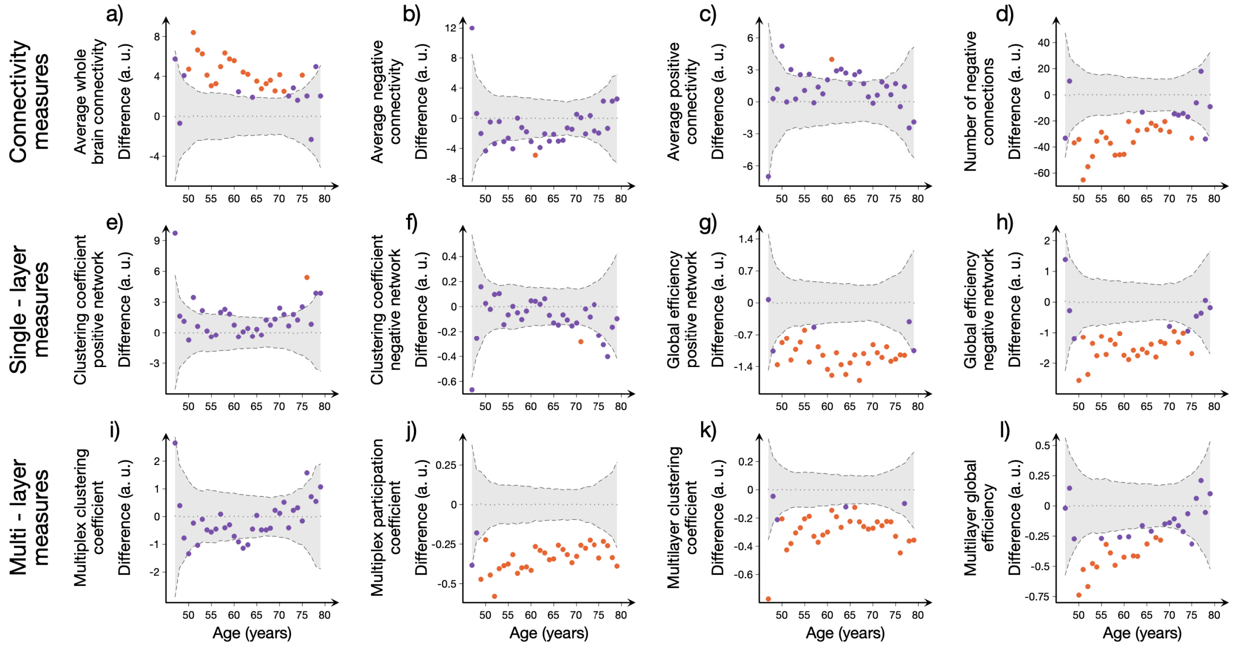

FIG. S4. **Differences between men and women in connectivity, single layer and multilayer measures.** Plots showing the differences between men and women (calculated as women - men) in a-d) simple connectivity measures: average whole-brain connectivity, average positive and negative connectivity and number of negative connections; e-f) single-layer topology measures: clustering coefficient and global efficiency of positive and negative networks; i-j) multiplex measures: clustering and participation coefficients and k-l) multilayer measures: clustering coefficient and global efficiency. The areas show the upper and lower bounds of the 95% confidence intervals (CI), and the differences in the corresponding measures between groups in blue circles as a function of individual's age. The differences are considered statistically significant if they fall outside the CIs. In particular, the orange circles show the differences that remained significant after applying a correction for multiple comparisons across the different age groups (FDR at $q < 0.05$)

IV. MULTILAYER MEASURES AS FUNCTION OF INTER-LAYER WEIGHT.

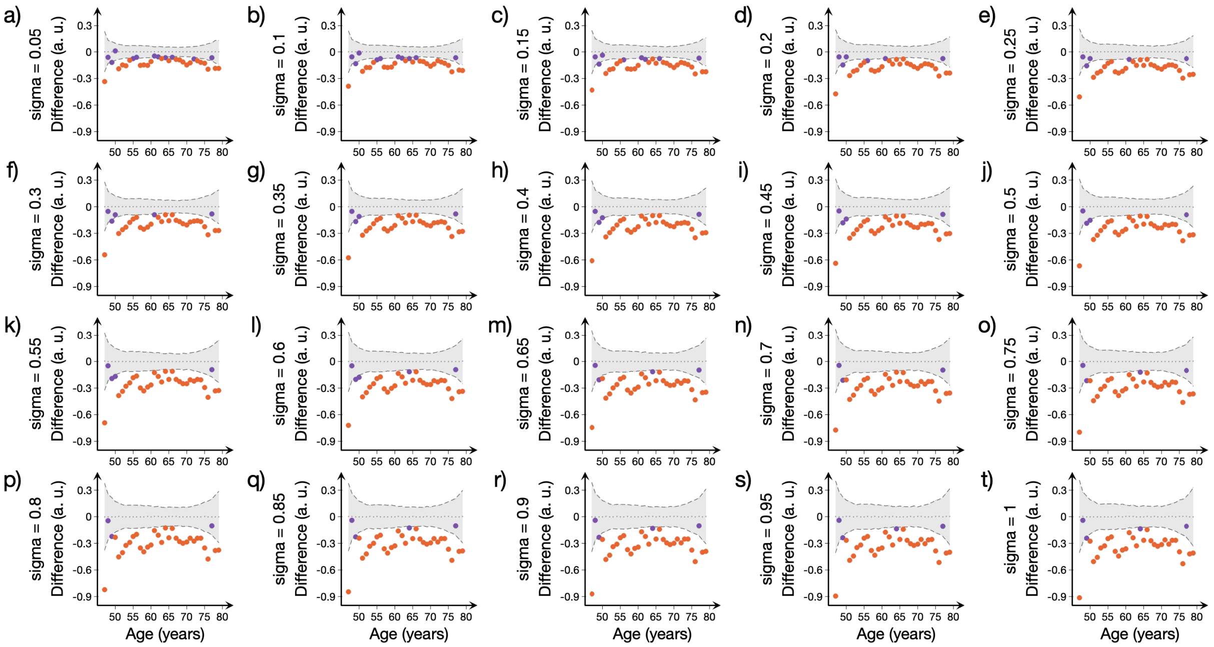

FIG. S5. **Differences between men and women in multilayer clustering**. Plots showing the differences between men and women (calculated as women — men) in the multilayer clustering for different values of inter-layer weight, ranging from a) $\sigma = 0.05$ to t) $\sigma = 1$. The areas show the upper and lower bounds of the 95% confidence intervals (CI), and the differences in the corresponding measures between groups in blue circles as a function of individual's age. The differences are considered statistically significant if they fall outside the CIs. In particular, the orange circles show the differences that remained significant after applying a correction for multiple comparisons across the different age groups (FDR at q < 0.05).

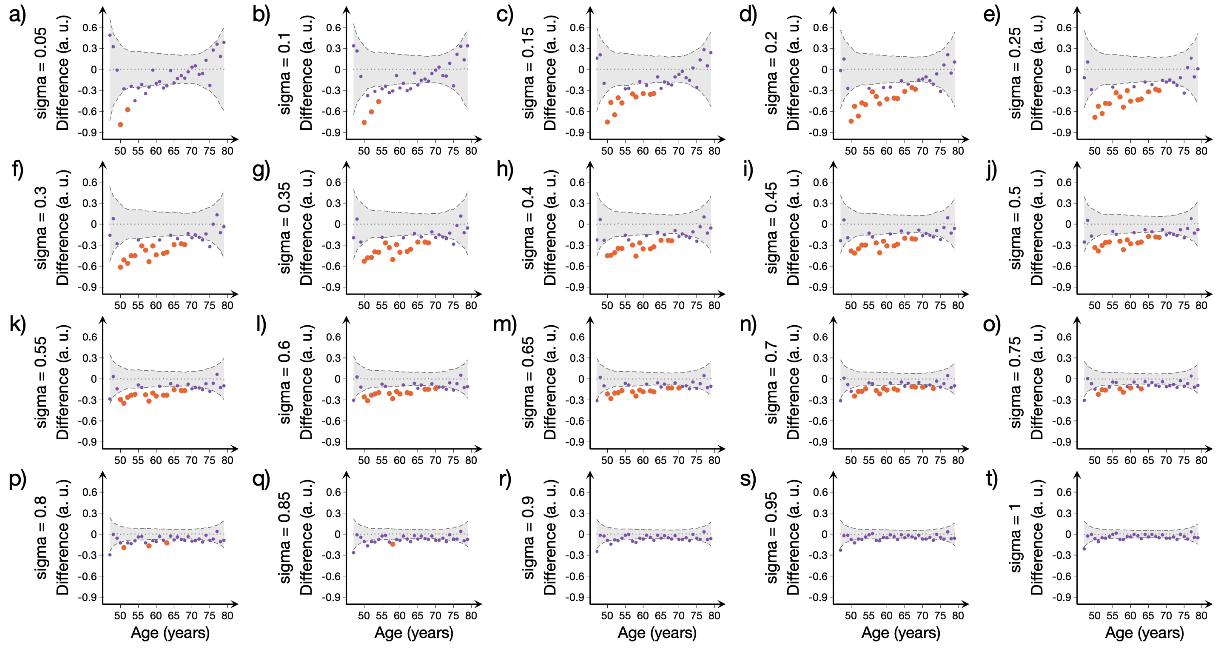

FIG. S6. **Differences between men and women in multilayer global efficiency**. Plots showing the differences between men and women (calculated as women — men) in the multilayer global efficiency for different values of inter-layer weight, ranging from a) $\sigma = 0.05$ to t) $\sigma = 1$. The areas show the upper and lower bounds of the 95% confidence intervals (CI), and the differences in the corresponding measures between groups in blue circles as a function of individual's age. The differences are considered statistically significant if they fall outside the CIs. In particular, the orange circles show the differences that remained significant after applying a correction for multiple comparisons across the different age groups (FDR at q < 0.05).

V. CORRELATION BETWEEN FUNCTIONAL CONNECTIVITY MEASURES.

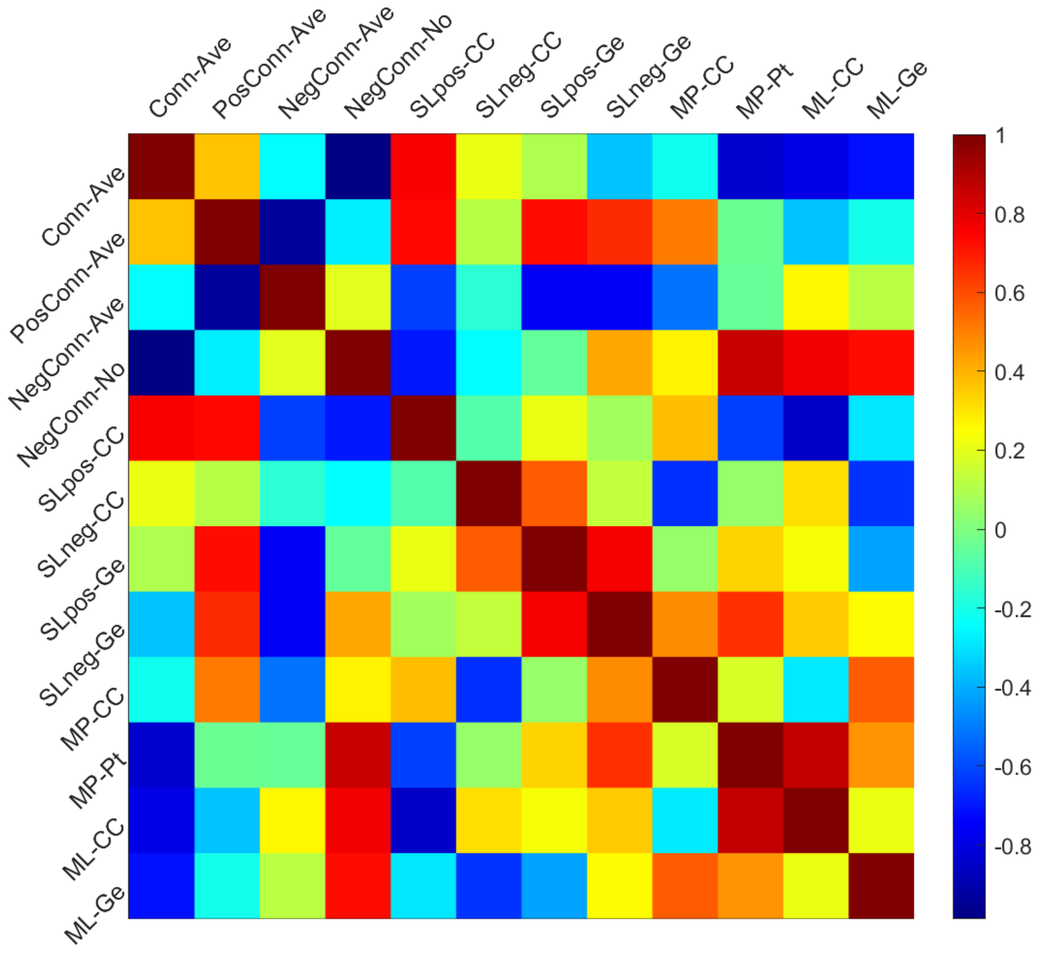

FIG. S7. **Correlation matrix between the different measures of functional connectivity**. Abbreviations: Conn-Ave: Average connectivity (VIF = 332.34); PosConn-Ave: Average positive connectivity (VIF = 67.90); NegConn-Ave: Average negative connectivity (VIF = 74.33); NegConn-No: Number of negative connections (VIF = 287.39); SLpos-CC and SLneg-CC: Single layer clustering coefficient for networks of positive and negative connections (VIF = 21.00 and 6.44); SLpos-Ge and SLneg-Ge: Single layer global efficiency for networks of positive and negative connections (VIF = 1489.2 and 1172.4); MP-CC: Multiplex clustering coefficient (VIF = 19.34); MP-Pt: Multiplex participation coefficient (VIF = 36.13); ML-CC and ML-Ge: Multilayer clustering coefficient and global efficiency (VIF = 128.42 and 772.01).

VI. GENOME-WIDE ASSOCIATION STUDY (GWAS) RESULTS: QUANTILE-QUANTILE PLOTS.

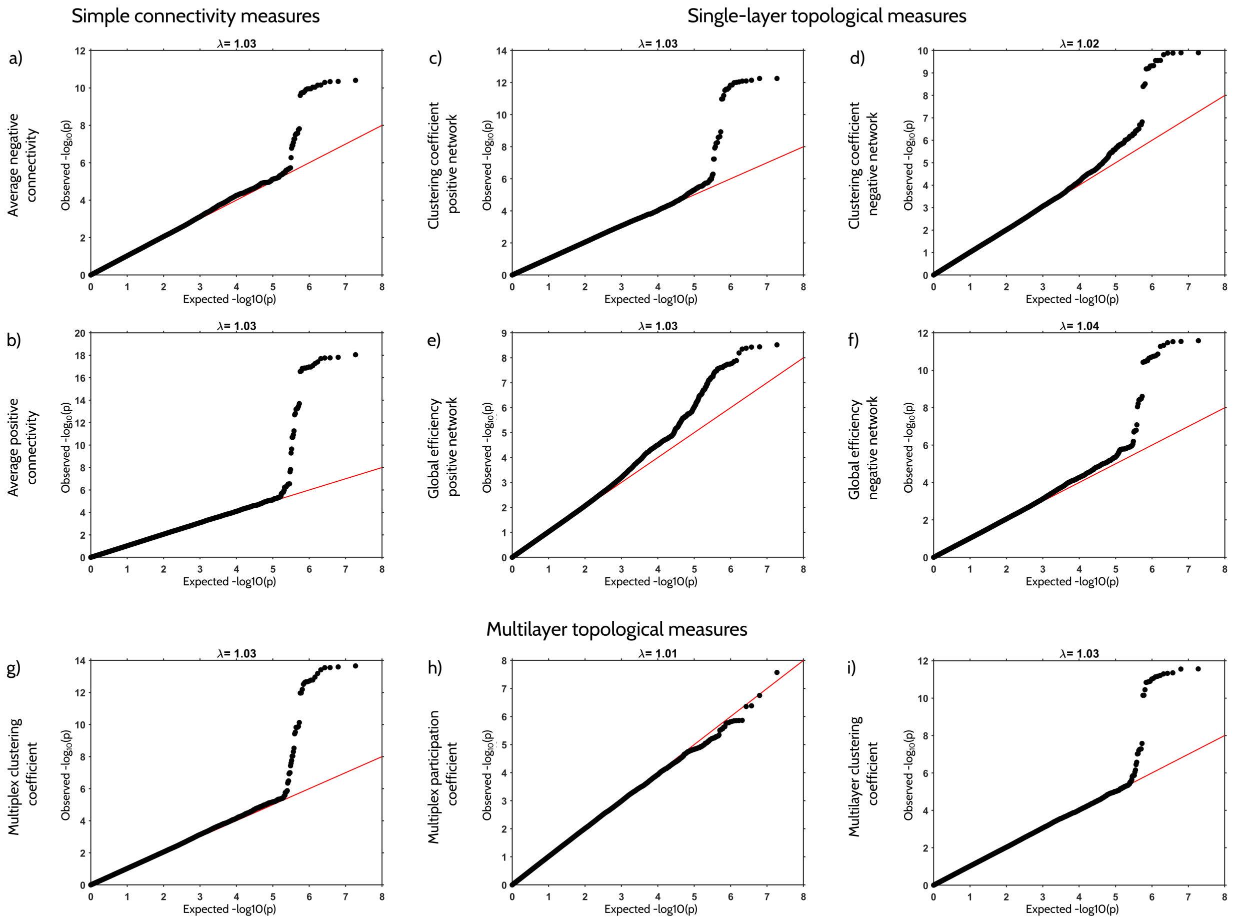

FIG. S8. **The quantile-quantile (QQ) plots for all functional connectivity measures that showed significant association with given genes**. QQ plots for all functional measures genomic inflation control (lambda) value. The red line shows the 95% confidence interval under the null hypothesis that there is no association among functional measures and SNPs. The black dots show the p-values of the complete study.

VII. MULTILAYER MEASURES UNCOVER SIMILAR PATTERNS OF BETWEEN-SEX DIFFERENCES FOR DIFFERENT SAMPLE SIZES.

We assessed the reproducibility of the multilayer measures as a function of sample size, by calculating sub-samples of men and women at each age, across a range of 40%-95% of the individuals included in the original sample. For each sub-sample, we randomly drew 100 sets of men and women, which were compared using permutation testing. The reproducibility was assessed as the percentage of the sets where we were able to detect between-sex differences that were also significant in the original sample. These results are shown in Supplementary Figs. S9 and S10 and they indicate that, at most ages, more than 90% of the sub-sampled sets with sizes as low as 55%-60% can also detect the differences identified in the original sample.

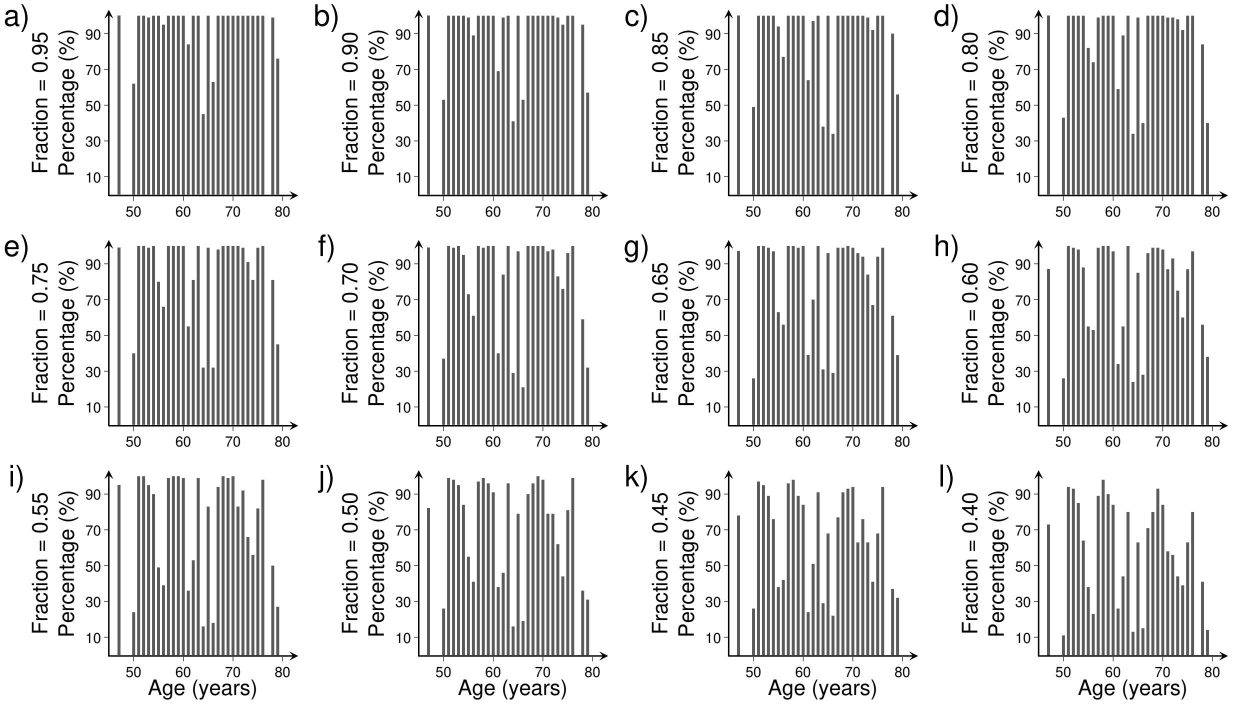

FIG. S9. **Reproducibility analysis for multilayer clustering coefficient**. Sub-samples of men and women were drawn from the original sample (range 40% to 95%) at each age, and compared between each other. For each sub-sample size, the comparison was drawn between 100 sets; the dark gray bars show the percentage of sets that were able to detect the between-sex differences that were significant in the original sample.

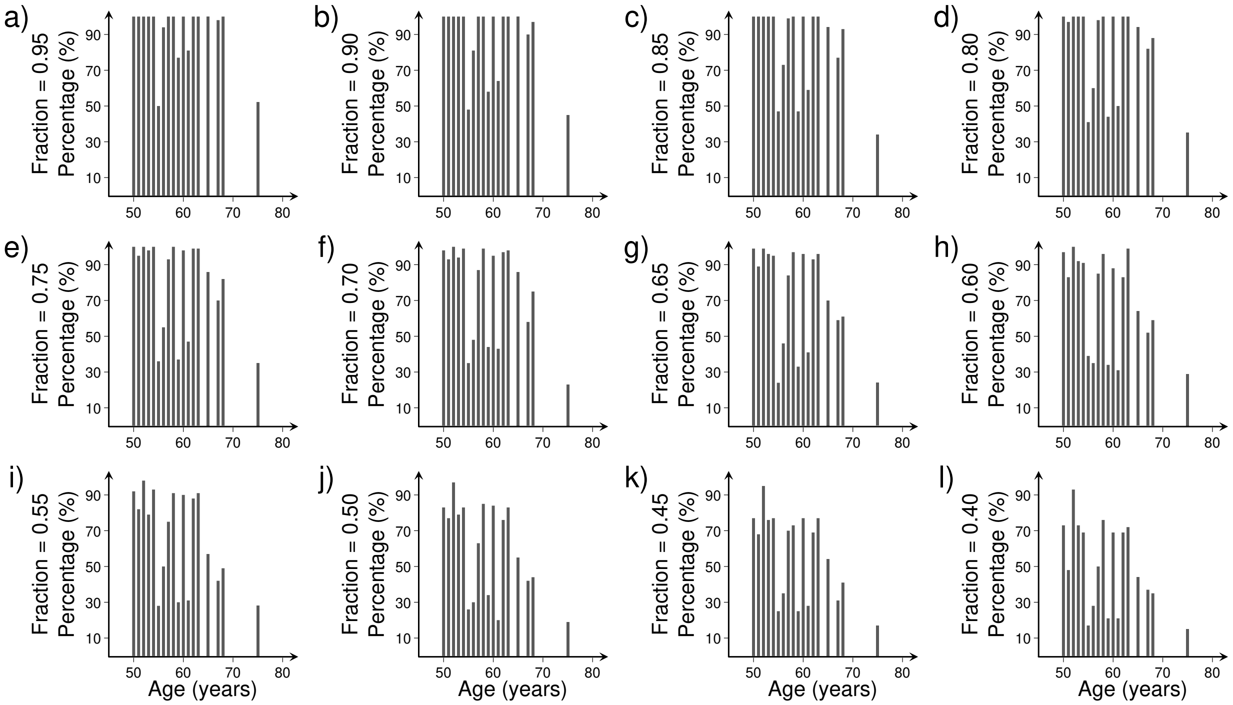

FIG. S10. **Reproducibility analysis for multilayer global efficiency**. Sub-samples of men and women were drawn from the original sample (range 40% to 95%) at each age, and compared between each other. For each sub-sample size, the comparison was drawn between 100 sets; the dark gray bars show the percentage of sets that were able to detect the between-sex differences that were significant in the original sample.

VIII. MULTILAYER MEASURES UNCOVER SIMILAR PATTERNS OF BETWEEN-SEX DIFFERENCES FOR INDIVIDUAL NETWORK DENSITIES.

We assessed whether our results were replicable at individual network densities (Supplementary Fig. S11). These analyses showed that the multilayer measures are less able to detect between-sex differences at low densities. However, we obtained highly reproducible results for all densities larger than 15%.

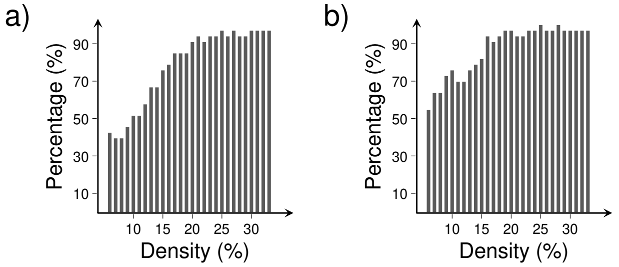

FIG. S11. **Multilayer network measures as a function of network density**. The percentage of age-range results calculated using the main AUC analysis in the density range 6% to 33% that can be reproduced at individual densities for a) multilayer clustering coefficient and b) multilayer global efficiency.

IX. DENSITY OF THE POSITIVE AND NEGATIVE NETWORKS.

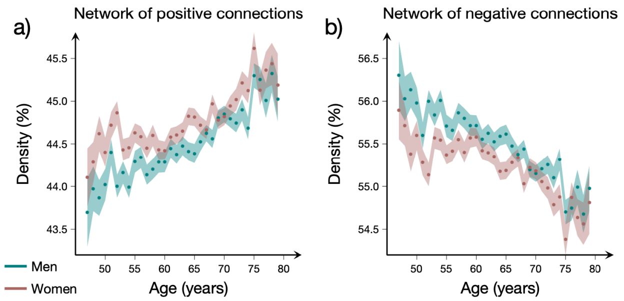

FIG. S12. **Density of the networks of positive and negative connections between men and women**. Plots showing the average density of the network of a) positive and b) negative connections for men (green) and women (red). The dots represent the mean density at a given age, while the shaded areas represent the standard error of the mean.

X. ILLUSTRATION OF THE AREA UNDER THE CURVE (AUC) ANALYSIS.

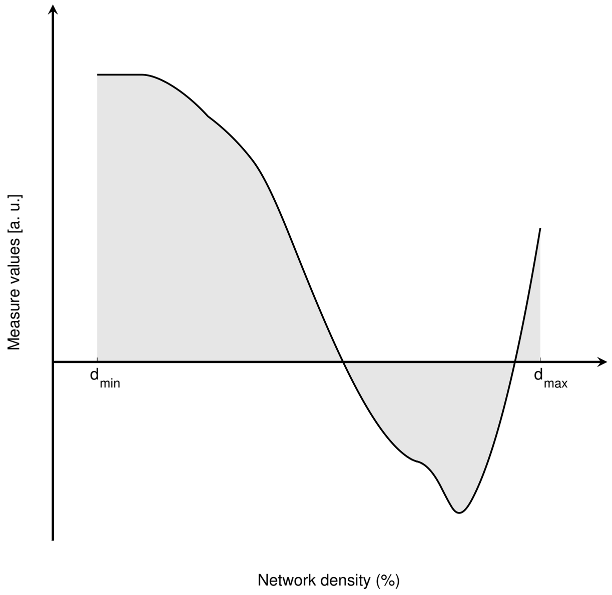

FIG. S13. **Illustration of the AUC analysis for the functional connectivity measures**. Each weighted connectivity network was binarized at a density range 6% to 33% in steps of 1%. We calculated each functional connectivity measure at all densities within this range and plotted the measure as a function of density (solid black line). For each measure, we integrated the total area under the curve (gray area). Calculated in this way, the AUC measure summarizes the behavior of the corresponding measure over the complete density range considered, and as such, it is less sensitive to the thresholding process.
