# Supplementary material for: Sex differences in multilayer functional network topology over the course of aging in 37543 UK Biobank participants
Source: Netw Neurosci. 2023 Jan 1;7(1):351–76. doi: 10.1162/netn_a_00286 (PMC10275214; doi:10.1162/netn_a_00286)
Supplement: Supplementary file 2 [file netn-7-1-351-s002.pdf]

Mijalkov, M., Veréb, D., Jamialahmadi, O., Canal-Garcia, A., Gómez-Ruiz, E., Vidal-Piñeiro, D., Romeo, S., Volpe, G. & Pereira, J. (2023). Supporting information for "Sex differences in multilayer functional network topology over the course of aging in 37543 UK Biobank participants. *Network Neuroscience*, *7*(1), 351–376. https://doi.org/10.1162/netn_a_00286

Table S1. **Results of genome-wide association study of brain traits and colocalization. Genome-wide significant (P < 5E-8) and independent (COJO-GCTA) variants for 9 brain traits have been shown.** Colocalization was conducted using all variants withing a window of 1 Mb around each lead variant. Genes with a H4 posterior probability > 0.8 were considered to colocalize with the GWAS locus. Conn-Ave: Average connectivity; PosConn-Ave: Average positive connectivity; SLpos-CC and SLneg-CC: Single layer clustering coefficient for networks of positive and negative connections; SLpos-Ge and SLneg-Ge: Single layer global efficiency for networks of positive and negative connections; MP-CC: Multiplex clustering coefficient; MP-Pt: Multiplex participation coefficient; ML-CC: Multilayer clustering coefficient.

FC measure	CHR	POS	SNP	Beta	SE	A1.freq	A2	A1	P	Nearest gene	eQTL.symbol	Max.H4.PP	Tissue(s)	Study
PosConn-Ave	2	114081827	rs62158169	0,083	0,009	0,786	T	C	8,9E-19	PAX8	IGKV1OR2-108	0,989	esophagus (muscularis);lung;skin (suprapubic);skin	GTEx
											FOXD4L1	0,970	thyroid	GTEx
											CBWD2	0,966	adrenal gland	GTEx
MP-CC	2	114081827	rs62158169	0,071	0,009	0,786	T	C	2,2E-14	PAX8	IGKV1OR2-108	0,990	esophagus (muscularis);lung;skin (suprapubic);skin	GTEx
											FOXD4L1	0,968	thyroid	GTEx
											CBWD2	0,965	adrenal gland	GTEx
SLpos-CC	2	114103966	rs56093896	0,068	0,009	0,788	A	C	5,6E-13	PAX8	IGKV1OR2-108	0,976	esophagus (muscularis);lung;skin (suprapubic);skin	GTEx
											FOXD4L1	0,949	thyroid	GTEx
											CBWD2	0,940	adrenal gland	GTEx
SLneg-Ge	2	114089551	rs2863957	0,065	0,009	0,781	A	C	2,6E-12	PAX8	IGKV1OR2-108	0,992	esophagus (muscularis);lung;skin (suprapubic);skin	GTEx
											FOXD4L1	0,976	thyroid	GTEx
											CBWD2	0,967	adrenal gland	GTEx
ML-CC	2	114106139	rs62158211	-0,066	0,009	0,788	T	G	2,8E-12	PAX8	IGKV1OR2-108	0,976	esophagus (muscularis);lung;skin (suprapubic);skin	GTEx
											FOXD4L1	0,943	thyroid	GTEx
											CBWD2	0,936	adrenal gland	GTEx
NegConn-Ave	2	114081827	rs62158169	-0,062	0,009	0,786	T	C	4,0E-11	PAX8	IGKV1OR2-108	0,988	esophagus (muscularis);lung;skin (suprapubic);skin	GTEx
											FOXD4L1	0,978	thyroid	GTEx
											CBWD2	0,959	adrenal gland	GTEx
SLneg-CC	2	114110568	rs62158213	-0,060	0,009	0,789	A	G	1,3E-10	PAX8	IGKV1OR2-108	0,978	esophagus (muscularis);lung;skin (suprapubic);skin	GTEx
											FOXD4L1	0,927	thyroid	GTEx
											CBWD2	0,929	adrenal gland	GTEx
SLpos-Ge	2	114085785	rs7556815	0,054	0,009	0,782	A	G	3,0E-09	PAX8	IGKV1OR2-108	0,993	esophagus (muscularis);lung;skin (suprapubic);skin	GTEx
											FOXD4L1	0,977	thyroid	GTEx
											CBWD2	0,967	adrenal gland	GTEx
SLpos-Ge	10	134300091	rs4309079	0,043	0,008	0,484	T	C	1,8E-08	INPP5A	INPP5A	0,878	brain (DLPFC)	CommonMind
MP-Pt	13	105359436	rs9514306	0,085	0,015	0,932	C	T	2,7E-08	DAOA	-	-	-	-
MP-CC	10	2002410	rs2152237	0,173	0,031	0,985	A	G	3,7E-08	ADARB2	-	-	-	-
