# Supplementary material for: Sex differences in multilayer functional network topology over the course of aging in 37543 UK Biobank participants
Source: Netw Neurosci. 2023 Jan 1;7(1):351–76. doi: 10.1162/netn_a_00286 (PMC10275214; doi:10.1162/netn_a_00286)
Supplement: Supplementary file 3 [file netn-7-1-351-s003.pdf]

Mijalkov, M., Veréb, D., Jamialahmadi, O., Canal-Garcia, A., Gómez-Ruiz, E., Vidal-Piñeiro, D., Romeo, S., Volpe, G. & Pereira, J. (2023). Supporting information for "Sex differences in multilayer functional network topology over the course of aging in 37543 UK Biobank participants. *Network Neuroscience*, *7*(1), 351–376. https://doi.org/10.1162/netn_a_00286

Sex differences in multilayer functional network topology over the course of aging in 37543 UK Biobank participants

Supplementary information

Linear models and PLS analysis

Mite Mijalkov,[1,*] Dániel Veréb,[1] Anna Canal Garcia,[1] Emiliano Gomez Ruiz,[2] Oveis Jamialahmadi,[3] Stefano Romeo,[3] Giovanni Volpe,[2] and Joana B. Pereira[1,4,*]

[1]*Department of Neurobiology, Care Sciences and Society, Karolinska Institutet, Stockholm, Sweden*

[2]*Department of Physics, Goteborg University, Goteborg, Sweden*

[3]*Department of Molecular and Clinical Medicine, Goteborg University, Goteborg, Sweden*

[4]*Memory Research Unit, Department of Clinical Sciences Malmö, Lund University, Lund, Sweden*

[*] Corresponding authors: Email: mite.mijalkov@ki.se // joana.pereira@ki.se. Address: KI, Dept. NVS, division of clinical geriatrics, Neo 7th floor, Blickagången 16, 141 83 Huddinge, Sweden.

I. PREDICTION OF FUNCTIONAL CONNECTIVITY MEASURES: SUMMARY OF BEST FITTING MODEL FOR EACH MEASURE.

TABLE S1: Summary of the best models for each measure of functional connectivity.

**Average connectivity** $Y \sim 1 + Age^2 + Age * Sex$					
	$\mathbf{R^2}$	**AIC**	**F − statistics**	**p − value**	
**Overall model**	0.769	244.651	55.2	< 0.000	
	**Age**	$\mathbf{Age^2}$	**Sex**	**Age ∗ Sex**	$\mathbf{Age^2}$ **∗ Sex**
**Coefficient**	0.606	-0.004	-10.054	0.101	−
**p-value**	0.024	0.112	< 0.000	0.010	−
**Average positive connectivity** $Y \sim 1 + Age + Sex$					
	$\mathbf{R^2}$	**AIC**	**F − statistics**	**p − value**	
**Overall model**	0.403	281.229	22.9	< 0.000	
	**Age**	$\mathbf{Age^2}$	**Sex**	**Age ∗ Sex**	$\mathbf{Age^2}$ **∗ Sex**
**Coefficient**	0.163	−	1.138	−	−
**p-value**	< 0.000	−	0.023	−	−
**Average negative connectivity** $Y \sim 1 + Age^2 + Sex$					
	$\mathbf{R^2}$	**AIC**	**F − statistics**	**p − value**	
**Overall model**	0.408	236.801	23.4	< 0.000	
	**Age**	$\mathbf{Age^2}$	**Sex**	**Age ∗ Sex**	$\mathbf{Age^2}$ **∗ Sex**
**Coefficient**	−	-0.001	-1.149	−	−
**p-value**	−	< 0.000	0.002	−	−
**Number of negative connections** $Y \sim 1 + Age * Sex$					
	$\mathbf{R^2}$	**AIC**	**F − statistics**	**p − value**	

Continued on next page

TABLE S1 – continued from previous page

**Overall model**	0.778	501.106	76.9	< 0.000	
	**Age**	**Age^2**	**Sex**	**Age ∗ Sex**	**Age^2 ∗ Sex**
**Coefficient**	-0.932	–	83.806	-0.895	–
**p-value**	< 0.000	–	< 0.000	0.001	–

Single layer: Clustering Coefficient - positive connections network

$Y \sim 1 + Age + Sex$

	**R^2**	**AIC**	**F – statistics**	**p – value**	
**Overall model**	0.459	226.729	28.6	< 0.000	
	**Age**	**Age^2**	**Sex**	**Age ∗ Sex**	**Age^2 ∗ Sex**
**Coefficient**	0.098	–	-1.579	–	–
**p-value**	< 0.000	–	< 0.000	–	–

Single layer: Clustering coefficient - negative connections network

$Y \sim 1 + Age + Sex$

	**R^2**	**AIC**	**F – statistics**	**p – value**	
**Overall model**	0.533	-96.294	38.1	< 0.000	
	**Age**	**Age^2**	**Sex**	**Age ∗ Sex**	**Age^2 ∗ Sex**
**Coefficient**	0.012	–	0.096	–	–
**p-value**	< 0.000	–	0.001	–	–

Single layer: Global efficiency - positive connections network

$Y \sim 1 + Age^2 + Sex$

	**R^2**	**AIC**	**F – statistics**	**p – value**	
**Overall model**	0.924	-1.051	396	< 0.000	
	**Age**	**Age^2**	**Sex**	**Age ∗ Sex**	**Age^2 ∗ Sex**
**Coefficient**	–	0.0005	1.089	–	–
**p-value**	–	< 0.000	< 0.000	–	–

Single layer: Global efficiency - negative connections network

$Y \sim 1 + Age^2 * Sex$

Continued on next page

	$\mathbf{R^2}$	**AIC**	**F − statistics**	**p − value**	
**Overall model**	0.649	88.603	41	< 0.000	
	**Age**	$\mathbf{Age^2}$	**Sex**	**Age ∗ Sex**	$\mathbf{Age^2}$ **∗ Sex**
**Coefficient**	–	0.0002	1.750	–	-0.0001
**p-value**	–	0.006	< 0.000	–	0.150
**Multiplex network: Clustering coefficient** $Y \sim 1 + Age + Age^2$					
	$\mathbf{R^2}$	**AIC**	**F − statistics**	**p − value**	
**Overall model**	0.096	106.989	4.45	0.016	
	**Age**	$\mathbf{Age^2}$	**Sex**	**Age ∗ Sex**	$\mathbf{Age^2}$ **∗ Sex**
**Coefficient**	-0.220	0.002	–	–	–
**p-value**	0.034	0.048	–	–	–
**Multiplex network: Participation coefficient** $Y \sim 1 + Age * Sex$					
	$\mathbf{R^2}$	**AIC**	**F − statistics**	**p − value**	
**Overall model**	0.891	-172.848	178	< 0.000	
	**Age**	$\mathbf{Age^2}$	**Sex**	**Age ∗ Sex**	$\mathbf{Age^2}$ **∗ Sex**
**Coefficient**	-0.005	–	0.556	-0.003	–
**p-value**	< 0.000	–	< 0.000	0.036	–
**Multilayer network: Clustering coefficient** $Y \sim 1 + Age^2 + Sex$					
	$\mathbf{R^2}$	**AIC**	**F − statistics**	**p − value**	
**Overall model**	0.799	-130.497	130	< 0.000	
	**Age**	$\mathbf{Age^2}$	**Sex**	**Age ∗ Sex**	$\mathbf{Age^2}$ **∗ Sex**
**Coefficient**	–	-0.0001	0.272	–	–
**p-value**	–	< 0.000	< 0.000	–	–
**Multilayer network: Global efficiency**					

Continued on next page

TABLE S1 – continued from previous page

Y ∼ 1 + Age ∗ Sex					
	$\mathbf{R^2}$	**AIC**	**F − statistics**	**p − value**	
**Overall model**	0.795	-82.061	85.1	< 0.000	
	**Age**	Age^2	**Sex**	$Age * Sex$	$Age^2 * Sex$
**Coefficient**	-0.015	–	1.027	-0.012	–
**p-value**	< 0.000	–	< 0.000	< 0.000	–

II. PREDICTION OF FUNCTIONAL CONNECTIVITY MEASURES: SUMMARY OF FULL MODEL FOR EACH MEASURE.

TABLE S2: Summary of the full linear models used in the prediction of all measures of functional connectivity. For each measure, the predictor variables include age, sex, Age^2, $\text{Age} * \text{Sex}$ and $\text{Age}^2 * \text{Sex}$.

Average connectivity<br>Full model parameters	
$R^2 = 0.768$	MSE = 2.194
AIC = 246.287	
Average positive connectivity<br>Full model parameters	
$R^2 = 0.507$	MSE = 3.259
AIC = 272.417	
Average negative connectivity<br>Full model parameters	
$R^2 = 0.501$	MSE = 1.696
AIC = 229.304	
Number of negative connections<br>Full model parameters	
$R^2 = 0.786$	MSE = 104.585
AIC = 501.333	
Clustering coefficient positive connections<br>Full model parameters	
$R^2 = 0.554$	MSE = 1.424
AIC = 217.751	
Clustering coefficient negative connections<br>Full model parameters	

Continued on next page

TABLE S2 – continued from previous page

$R^2 = 0.564$	MSE = 0.012
AIC = −97.035	
Global efficiency positive connections<br>Full model parameters	
$R^2 = 0.934$	MSE = 0.048
AIC = −6.367	
Global efficiency negative connections<br>Full model parameters	
$R^2 = 0.728$	MSE = 0.162
AIC = 74.259	
Multiplex clustering coefficient<br>Full model parameters	
$R^2 = 0.294$	MSE = 0.220
AIC = 94.458	
Multiplex participation coefficient<br>Full model parameters	
$R^2 = 0.890$	MSE = 0.004
AIC = −169.56	
Multilayer clustering coefficient<br>Full model parameters	
$R^2 = 0.805$	MSE = 0.007
AIC = −128.689	
Multilayer global efficiency<br>Full model parameters	
$R^2 = 0.817$	MSE = 0.014
AIC = −87.031	

III. PARTIAL LEAST SQUARES REGRESSION ANALYSIS (PLS) DETAILS.

TABLE S3: **Relationship between executive domain test scores and connectivity, single-layer, multiplex and multilayer measures.** Details of the corresponding PLS analysis show the loadings in each latent variable, the VIP scores of each measure and the variance explained by the latent variables and the total model. Abbreviations: LV: latent variables; load.: loadings; Conn-Ave: Average connectivity; PosConn-Ave: Average positive connectivity; NegConn-Ave: Average negative connectivity; NegConn-No: Number of negative connections; SLpos-CC and SLneg-CC: Single layer clustering coefficient for networks of positive and negative connections; SLpos-Ge and SLneg-Ge: Single layer global efficiency for networks of positive and negative connections; MP-CC: Multiplex clustering coefficient; MP-Pt: Multiplex participation coefficient; ML-CC and ML-Ge: Multilayer clustering coefficient and global efficiency.

**Executive domain**							
Variables	LV1 load.	LV2 load.	LV3 load.	LV4 load.	LV5 load.	LV6 load.	VIP scores
Age	7,13	0,62	2,60	0,72	1,40	-1,22	1,82
Sex	1,93	5,12	-4,97	0,53	-1,14	2,38	0,68
Conn-ave	3,42	-4,94	4,79	-0,43	-1,04	1,55	0,54
PosConn-Ave	6,38	-2,13	-3,96	-0,09	0,33	0,66	1,08
NegConn-Ave	-6,01	1,54	4,40	-1,02	0,75	1,46	1,06
NegConn-No	-2,98	4,62	-5,36	-0,14	1,52	-0,82	0,53
SLpos-CC	5,00	-5,83	0,25	-0,30	1,19	1,26	0,81
SLneg-CC	4,72	3,96	2,59	-3,91	0,85	-0,78	1,29
SLpos-Ge	6,77	2,93	-2,42	1,13	-0,64	0,01	1,57
SLneg-Ge	4,12	1,86	-5,95	2,33	-0,26	0,02	0,97
MP-CC	0,57	-3,93	-6,66	0,63	0,28	-0,69	0,77
MP-Pt	-0,65	5,33	-5,49	-0,11	-0,35	0,61	0,35
ML-CC	-3,68	5,63	-3,98	0,48	0,03	0,81	0,59
ML-Ge	-3,77	1,86	-6,22	1,77	0,59	-0,52	0,77
**Variance explained by latent variables**							

Continued on next page

TABLE S3 – continued from previous page

	LV1	LV2	LV3	LV4	LV5	LV6	Total variance
Predictor matrix	0,33	0,25	0,33	0,03	0,01	0,02	0,98
Predicted variable	0,72	0,08	0,02	0,08	0,02	0,00	0,92

TABLE S3: **Relationship between visuospatial domain test scores and connectivity, single-layer, multiplex and multilayer measures.** Details of the corresponding PLS analysis show the loadings in each latent variable, the VIP scores of each measure and the variance explained by the latent variables and the total model. Abbreviations are as Supplementary Table S2 and Fig. 4.

**Visuospatial domain**							
Variables	LV1 load.	LV2 load.	LV3 load.	LV4 load.	LV5 load.	LV6 load.	VIP scores
Age	-6,49	-4,08	-0,98	-0,84	-1,52	-0,10	1,76
Sex	3,03	-5,88	3,59	2,24	-0,55	0,36	0,84
Conn-ave	-6,90	3,45	-0,13	1,67	-0,36	0,01	1,14
PosConn-Ave	-4,12	-2,93	5,75	-1,36	0,52	-0,85	0,77
NegConn-Ave	3,47	3,63	-5,12	2,87	-0,89	-1,04	0,77
NegConn-No	6,66	-3,51	1,04	-1,40	0,51	-0,90	1,08
SLpos-CC	-6,46	1,62	3,60	-1,38	0,50	-1,21	1,05
SLneg-CC	-3,25	-5,33	-3,28	2,14	2,64	-1,29	0,99
SLpos-Ge	-3,13	-6,50	2,89	0,38	-0,98	0,85	0,82
SLneg-Ge	0,09	-5,33	5,36	-1,60	-0,47	1,01	0,25
MP-CC	0,68	0,72	6,31	-4,29	0,65	-1,16	0,40
MP-Pt	5,24	-5,03	2,35	0,80	-0,40	-1,32	0,86
ML-CC	7,07	-3,19	0,30	1,07	-0,26	-0,24	1,19
ML-Ge	6,68	-1,14	2,82	-2,45	0,51	0,67	1,18
**Variance explained by latent variables**							

Continued on next page

TABLE S3 – continued from previous page

	LV1	LV2	LV3	LV4	LV5	LV6	Total variance
Predictor matrix	0,40	0,27	0,21	0,06	0,02	0,01	0,98
Predicted variable	0,78	0,06	0,04	0,07	0,01	0,00	0,96

TABLE S4: **Relationship between prevalence of high blood pressure and connectivity, single-layer, multiplex and multilayer measures.** Details of the corresponding PLS analysis show the loadings in each latent variable, the VIP scores of each measure and the variance explained by the latent variables and the total model. Abbreviations are as Supplementary Table S2 and Fig. 4.

**Blood pressure**					
Variables	LV1 load.	LV2 load.	LV3 load.	LV4 load.	VIP scores
Age	-0,58	1,12	1,35	-0,73	0,24
Sex	7,56	2,21	0,12	0,88	1,78
Conn-ave	-6,99	3,51	1,34	0,02	1,08
PosConn-Ave	1,46	-0,30	7,53	-1,69	0,40
NegConn-Ave	-2,95	1,37	-7,09	0,57	0,51
NegConn-No	7,00	-3,28	-0,52	-0,65	1,06
SLpos-CC	-3,69	-0,84	6,97	-0,14	0,64
SLneg-CC	-3,69	-0,84	6,97	-0,14	0,64
SLpos-Ge	6,35	2,83	3,52	-1,20	1,45
SLneg-Ge	6,08	-1,34	4,94	0,43	1,02
MP-CC	1,34	-3,35	6,44	-2,78	0,62
MP-Pt	7,51	0,71	-1,56	-0,43	1,38
ML-CC	5,91	0,97	-5,20	0,98	1,09
ML-Ge	3,52	-5,54	3,91	1,96	0,79
**Variance explained by latent variables**					

Continued on next page

TABLE S4 – continued from previous page

	LV1	LV2	LV3	LV4	Total variance
Predictor matrix	0,41	0,09	0,36	0,02	0,89
Predicted variable	0,39	0,08	0,01	0,04	0,51

TABLE S5: **Relationship between prevalence of heart attack and connectivity, single-layer, multiplex and multilayer measures.** Details of the corresponding PLS analysis show the loadings in each latent variable, the VIP scores of each measure and the variance explained by the latent variables and the total model. Abbreviations are as Supplementary Table S2 and Fig. 4.

**Heart attack**				
Variables	LV1 load.	LV2 load.	LV3 load.	VIP scores
Age	-0,48	-0,93	2,37	0,27
Sex	6,70	1,00	1,78	2,78
Conn-ave	-1,66	1,19	5,98	0,38
PosConn-Ave	2,98	-5,89	2,27	0,44
NegConn-Ave	-2,89	5,96	-2,26	0,48
NegConn-No	2,26	-2,29	-6,02	0,75
SLpos-CC	0,97	-5,89	3,13	0,63
SLneg-CC	0,97	-5,89	3,13	0,63
SLpos-Ge	4,75	-3,54	2,37	1,08
SLneg-Ge	4,57	-5,27	0,35	0,82
MP-CC	2,76	-6,30	1,38	0,40
MP-Pt	4,54	1,55	-3,74	1,30
ML-CC	0,38	5,68	-4,08	0,67
ML-Ge	1,57	-4,29	-3,09	0,36
**Variance explained by latent variables**				

Continued on next page

TABLE S5 – continued from previous page

	LV1	LV2	LV3	Total variance
Predictor matrix	0,20	0,39	0,22	0,81
Predicted variable	0,48	0,09	0,04	0,61

TABLE S6: **Relationship between subcortical volumes and connectivity, single-layer, multiplex and multilayer measures.** Details of the corresponding PLS analysis show the loadings in each latent variable, the VIP scores of each measure and the variance explained by the latent variables and the total model. Abbreviations are as Supplementary Table S2 and Fig. 4.

**Subcortical volumes**						
Variables	LV1 load.	LV2 load.	LV3 load.	LV4 load.	LV5 load.	VIP scores
Age	-6,32	-4,19	1,39	-2,12	-0,42	1,43
Sex	4,19	-4,93	4,62	0,43	-0,54	0,85
Conn-Ave	-7,74	1,22	-0,22	1,72	-0,37	1,34
PosConn-Ave	-3,71	-0,29	6,58	-2,51	-0,03	0,63
NegConn-Ave	2,93	0,78	-6,67	2,93	-0,64	0,51
NegConn-No	7,65	-1,07	0,82	-1,88	0,22	1,32
SLpos-CC	-6,28	3,11	3,18	-1,62	0,99	1,02
SLneg-CC	-3,12	-6,98	-0,58	0,56	2,06	0,82
SLpos-Ge	-2,07	-5,29	5,34	-1,51	-1,22	0,55
SLneg-Ge	1,97	-2,64	6,79	-2,47	0,65	0,34
MP-CC	1,69	4,62	5,05	-3,54	-0,34	0,51
MP-Pt	6,37	-3,40	3,11	-0,16	-0,60	1,09
ML-CC	7,48	-2,77	0,11	0,66	-0,24	1,26
ML-Ge	7,42	1,33	1,62	-1,35	1,61	1,38
**Variance explained by latent variables**						
	LV1	LV2	LV3	LV4	LV5	Total variance

Continued on next page

TABLE S6 – continued from previous page

Predictor matrix	0,45	0,20	0,26	0,06	0,01	0,98
Predicted variable	0,84	0,03	0,01	0,04	0,01	0,93

TABLE S7: **Relationship between white matter hyperintensities and connectivity, single-layer, multiplex and multilayer measures.** Details of the corresponding PLS analysis show the loadings in each latent variable, the VIP scores of each measure and the variance explained by the latent variables and the total model. Abbreviations are as Supplementary Table S2 and Fig. 4.

**White matter hyperintensities**						
Variables	LV1 load.	LV2 load.	LV3 load.	LV4 load.	LV5 load.	VIP scores
Age	7,48	1,62	1,74	1,27	0,29	1,72
Sex	-0,75	7,33	-2,48	-1,50	0,88	0,54
Conn-ave	6,20	-4,48	1,74	-1,60	0,43	0,98
PosConn-Ave	6,06	1,03	-5,01	0,59	0,06	1,07
NegConn-Ave	-5,45	-1,84	5,27	-1,22	0,51	1,02
NegConn-No	-5,92	4,57	-2,26	1,54	-0,38	0,92
SLpos-CC	5,91	-4,20	-3,00	0,38	-1,18	0,89
SLneg-CC	4,01	4,71	4,58	-0,19	-1,89	1,21
SLpos-Ge	5,37	5,46	-2,10	-0,11	1,04	1,29
SLneg-Ge	1,48	5,31	-5,69	0,38	-0,79	0,51
MP-CC	-0,75	-1,16	-7,20	2,86	0,54	0,54
MP-Pt	-3,55	6,66	-2,29	-0,51	0,52	0,49
ML-CC	-5,80	5,48	-0,22	-0,48	0,17	0,84
ML-Ge	-6,34	2,51	-3,84	0,90	-1,47	1,14
**Variance explained by latent variables**						
	LV1	LV2	LV3	LV4	LV5	Total variance

Continued on next page

TABLE S7 – continued from previous page

Predictor matrix	0,40	0,31	0,23	0,02	0,01	0,97
Predicted variable	0,78	0,07	0,02	0,07	0,01	0,95
