# Supplementary material for: Sex differences in multilayer functional network topology over the course of aging in 37543 UK Biobank participants
Source: Netw Neurosci. 2023 Jan 1;7(1):351–76. doi: 10.1162/netn_a_00286 (PMC10275214; doi:10.1162/netn_a_00286)
Supplement: Supplementary file 4 [file netn-7-1-351-s004.pdf]

Mijalkov, M., Veréb, D., Jamialahmadi, O., Canal-Garcia, A., Gómez-Ruiz, E., Vidal-Piñeiro, D., Romeo, S., Volpe, G. & Pereira, J. (2023). Supporting information for "Sex differences in multilayer functional network topology over the course of aging in 37543 UK Biobank participants. *Network Neuroscience*, *7*(1), 351–376. https://doi.org/10.1162/netn_a_00286

Sex differences in multilayer functional network topology over the course of aging in 37543 UK Biobank participants

Supplementary information

Power analysis

Mite Mijalkov,[1,*] Dániel Veréb,[1] Anna Canal Garcia,[1] Emiliano Gomez Ruiz,[2] Oveis Jamialahmadi,[3] Stefano Romeo,[3] Giovanni Volpe,[2] and Joana B. Pereira[1,4,*]

[1]*Department of Neurobiology, Care Sciences and Society, Karolinska Institutet, Stockholm, Sweden*

[2]*Department of Physics, Goteborg University, Goteborg, Sweden*

[3]*Department of Molecular and Clinical Medicine, Goteborg University, Goteborg, Sweden*

[4]*Memory Research Unit, Department of Clinical Sciences Malmö, Lund University, Lund, Sweden*

[*] Corresponding authors: Email: mite.mijalkov@ki.se // joana.pereira@ki.se. Address: KI, Dept. NVS, division of clinical geriatrics, Neo 7th floor, Blickagången 16, 141 83 Huddinge, Sweden.

Table S1: Effect sizes and power analysis of the comparison tests. The table contains: MD effect-size, the mean difference effect size for differences between women and men; CI-low and CI-high, the lower and higher bound of 95% confidence interval for the effect size; and power of the statistical test to detect the reported effect size.

Average whole brain connectivity				
Age	MD effect-size	CI-low	CI-high	Power (%)
47	-5,743	-13,537	2,025	43,490
48	0,715	-4,434	6,002	8,440
49	-4,084	-8,302	0,239	57,340
50	-4,737	-8,464	-1,030	79,100
51	-8,402	-11,790	-5,064	99,970
52	-6,625	-9,525	-3,755	99,740
53	-6,248	-8,999	-3,447	99,610
54	-4,137	-6,886	-1,399	89,840
55	-3,053	-5,873	-0,214	68,040
56	-3,266	-5,992	-0,548	76,520
57	-4,988	-7,619	-2,220	96,620
58	-6,359	-8,870	-3,855	99,910
59	-5,758	-8,389	-3,196	99,410
60	-5,567	-7,997	-3,057	99,680
61	-2,440	-4,771	-0,127	66,480
62	-4,410	-6,829	-1,953	96,980
63	-4,194	-6,408	-1,888	97,860
64	-1,905	-4,175	0,292	52,080
65	-3,514	-5,735	-1,267	91,700
66	-2,760	-4,994	-0,513	77,430
67	-3,281	-5,495	-1,031	89,680
68	-3,620	-5,778	-1,453	95,260
69	-2,557	-4,702	-0,336	72,890
70	-4,181	-6,340	-2,063	97,880
71	-2,511	-4,835	-0,308	70,890
72	-2,015	-4,574	0,513	44,540
73	-2,857	-5,402	-0,124	66,040
74	-1,596	-4,765	1,501	26,770
75	-4,131	-7,552	-0,840	79,750
76	-2,015	-5,720	1,789	28,420
77	2,325	-2,022	6,493	29,710
78	-4,990	-9,982	-0,310	63,070
79	-2,027	-8,210	4,294	16,270
Average negative connectivity				
Age	MD effect-size	CI-low	CI-high	Power (%)
47	7,010	-2,591	15,236	46,550

48	-0,317	-5,834	5,338	6,190
49	-1,185	-5,800	3,557	12,970
50	-5,230	-9,458	-1,144	82,330
51	0,025	-3,191	3,139	5,180
52	-3,016	-5,944	-0,067	62,030
53	-0,267	-3,061	2,558	7,210
54	-2,535	-5,554	0,450	47,640
55	-1,051	-3,871	1,715	17,550
56	-2,596	-5,541	0,157	56,670
57	0,088	-2,674	2,760	5,660
58	-1,385	-4,100	1,213	27,160
59	-0,742	-3,358	1,850	13,870
60	-2,057	-4,673	0,588	46,270
61	-3,971	-6,421	-1,503	94,080
62	-2,898	-5,419	-0,357	72,930
63	-3,047	-5,412	-0,628	80,330
64	-2,688	-5,148	-0,296	70,640
65	-1,700	-4,082	0,676	41,060
66	-2,531	-4,829	-0,379	71,010
67	-2,825	-5,012	-0,670	81,240
68	-1,695	-3,796	0,502	43,920
69	-0,477	-2,701	1,769	10,680
70	0,141	-2,229	2,510	6,350
71	-0,610	-3,047	1,794	12,180
72	-1,853	-4,383	0,698	43,470
73	-1,482	-4,215	1,206	29,430
74	-0,651	-3,865	2,728	10,540
75	-1,720	-5,019	1,381	25,420
76	0,450	-3,455	4,701	7,810
77	-1,440	-5,835	3,144	15,160
78	2,453	-3,224	8,410	21,690
79	1,880	-4,091	8,202	15,820
Average positive connectivity				
Age	MD effect-size	CI-low	CI-high	Power (%)
47	-11,973	-21,363	-1,327	75,490
48	-0,647	-7,368	6,038	7,350
49	2,024	-3,778	7,749	16,940
50	4,312	-0,345	9,166	55,770
51	0,476	-3,602	4,331	7,640
52	3,372	-0,209	6,877	58,320
53	0,445	-2,825	3,905	8,260
54	3,089	-0,527	6,725	51,620
55	2,644	-0,757	6,161	42,690
56	4,049	0,584	7,638	72,970

57	-0,029	-3,424	3,285	5,160
58	1,229	-2,056	4,437	17,780
59	1,784	-1,396	4,943	29,970
60	3,113	-0,076	6,275	61,970
61	4,921	1,974	7,868	93,880
62	3,907	0,842	7,158	79,860
63	2,061	-0,820	4,922	40,640
64	3,031	0,221	5,827	68,750
65	2,141	-0,767	4,993	43,210
66	3,022	0,258	5,763	69,420
67	2,921	0,184	5,587	68,100
68	1,260	-1,256	3,986	23,940
69	1,430	-1,299	4,197	28,210
70	-0,517	-3,413	2,429	9,570
71	-0,099	-3,071	2,814	5,880
72	2,047	-1,173	5,129	35,070
73	-0,351	-3,600	2,923	7,440
74	1,696	-2,135	5,533	21,700
75	1,970	-1,814	5,928	24,540
76	-2,278	-7,104	2,407	24,440
77	1,366	-3,895	6,711	12,250
78	-2,300	-9,355	4,298	17,140
79	-2,573	-9,368	4,588	18,090

Number of negative connections

Age	MD effect-size	CI-low	CI-high	Power (%)
47	33,329	-23,917	90,119	31,460
48	-10,549	-45,837	23,827	14,540
49	36,907	8,812	66,403	79,520
50	34,089	7,508	59,505	82,460
51	65,111	42,458	86,980	100,000
52	55,188	35,452	74,343	99,980
53	47,241	28,367	65,676	99,960
54	35,618	17,844	53,821	98,400
55	28,742	10,187	46,308	91,320
56	32,818	14,185	50,989	95,960
57	37,079	18,841	54,675	98,780
58	46,282	29,239	63,461	99,980
59	45,972	29,104	63,473	99,990
60	45,599	29,333	61,315	100,000
61	20,472	5,050	35,809	82,450
62	36,445	20,113	52,853	99,660
63	27,506	12,255	42,850	96,790
64	13,468	-1,147	28,391	54,760
65	26,726	11,787	41,659	96,590

66	21,973	7,329	37,611	88,240
67	23,733	9,086	38,271	93,050
68	27,018	12,634	41,473	97,210
69	20,573	6,427	35,009	87,110
70	28,492	13,958	42,659	98,860
71	14,603	-0,313	29,576	59,900
72	15,531	-0,877	32,662	54,970
73	14,354	-3,943	31,970	48,030
74	17,078	-2,997	38,348	51,490
75	33,102	9,836	55,810	89,730
76	6,217	-18,658	31,005	12,410
77	-18,028	-45,074	9,177	36,490
78	33,830	2,718	68,027	65,720
79	9,006	-32,054	49,946	11,490

Clustering coefficient - positive network

Age	MD effect-size	CI-low	CI-high	Power (%)
47	-9,716	-15,919	-2,870	88,830
48	-1,632	-5,628	2,606	19,380
49	-1,142	-4,519	2,395	16,080
50	0,735	-2,119	3,619	11,850
51	-3,462	-6,019	-0,982	83,510
52	-0,638	-2,927	1,652	14,170
53	-2,161	-4,324	-0,048	63,320
54	-0,177	-2,446	2,098	6,670
55	0,404	-1,772	2,623	10,070
56	0,243	-1,894	2,500	7,830
57	-1,984	-4,049	0,145	56,350
58	-2,302	-4,350	-0,235	70,790
59	-1,854	-3,871	0,231	54,540
60	-0,759	-2,734	1,309	17,690
61	0,388	-1,439	2,234	11,530
62	-0,073	-2,002	1,851	5,810
63	-0,403	-2,208	1,361	11,380
64	0,369	-1,445	2,211	11,110
65	-0,353	-2,186	1,451	9,710
66	0,226	-1,480	1,986	8,460
67	-1,264	-3,001	0,513	40,410
68	-0,771	-2,451	0,982	21,750
69	-1,332	-3,142	0,499	42,860
70	-2,411	-4,237	-0,612	81,680
71	-1,782	-3,665	0,078	58,690
72	-0,681	-2,737	1,328	16,510
73	-1,804	-4,007	0,397	48,100
74	-1,255	-3,727	1,272	25,570

75	-2,538	-5,196	0,132	57,270
76	-5,420	-8,610	-2,284	96,610
77	-0,855	-4,277	2,645	12,380
78	-3,852	-8,373	0,308	55,660
79	-3,862	-8,359	0,604	51,100
Clustering coefficient - negative network				
Age	MD effect-size	CI-low	CI-high	Power (%)
47	0,667	0,005	1,333	60,910
48	0,256	-0,220	0,750	27,560
49	-0,157	-0,544	0,200	19,600
50	-0,027	-0,305	0,248	6,980
51	0,022	-0,213	0,256	7,450
52	-0,097	-0,314	0,120	21,890
53	-0,104	-0,300	0,102	26,220
54	0,144	-0,062	0,355	36,940
55	0,066	-0,146	0,282	15,060
56	-0,003	-0,215	0,209	5,340
57	0,049	-0,146	0,259	11,700
58	0,102	-0,093	0,298	26,640
59	0,036	-0,150	0,232	10,140
60	-0,048	-0,233	0,140	13,080
61	-0,042	-0,220	0,133	12,630
62	-0,021	-0,203	0,166	7,680
63	-0,064	-0,241	0,111	17,200
64	0,069	-0,104	0,250	18,930
65	0,129	-0,048	0,299	42,610
66	0,148	-0,020	0,317	53,210
67	0,067	-0,103	0,240	18,630
68	0,105	-0,068	0,286	31,750
69	0,154	-0,015	0,328	54,990
70	0,132	-0,041	0,303	47,010
71	0,283	0,118	0,454	94,110
72	0,019	-0,169	0,205	7,430
73	0,081	-0,125	0,283	19,010
74	-0,014	-0,248	0,219	6,410
75	0,233	-0,019	0,478	56,130
76	0,308	0,006	0,601	66,580
77	0,402	0,056	0,750	73,990
78	0,164	-0,258	0,563	19,550
79	0,097	-0,416	0,597	10,640
Global efficiency - positive network				
Age	MD effect-size	CI-low	CI-high	Power (%)

47	-0,070	-1,805	1,790	5,780
48	1,062	-0,088	2,233	54,960
49	1,364	0,440	2,277	89,790
50	0,878	0,041	1,706	68,590
51	0,780	0,107	1,458	71,950
52	1,257	0,634	1,866	98,970
53	1,023	0,438	1,622	96,070
54	0,866	0,253	1,462	87,160
55	0,600	0,038	1,165	66,550
56	1,302	0,714	1,868	99,770
57	0,530	-0,032	1,093	58,200
58	0,966	0,429	1,514	96,900
59	1,159	0,625	1,705	99,620
60	1,461	0,947	2,001	100,000
61	1,592	1,063	2,104	100,000
62	1,105	0,585	1,620	99,550
63	1,363	0,865	1,865	99,990
64	1,574	1,078	2,070	100,000
65	1,327	0,828	1,829	99,980
66	1,143	0,666	1,618	99,850
67	1,702	1,253	2,164	100,000
68	1,123	0,654	1,592	99,880
69	1,336	0,870	1,803	99,990
70	0,927	0,454	1,396	98,310
71	1,103	0,613	1,593	99,740
72	1,199	0,646	1,752	99,550
73	0,969	0,359	1,552	92,810
74	1,286	0,614	1,950	98,450
75	1,243	0,537	1,972	95,500
76	1,144	0,336	1,938	88,080
77	1,152	0,218	2,079	76,790
78	0,409	-0,788	1,527	17,440
79	1,055	-0,200	2,363	45,570

Global efficiency - negative network

Age	MD effect-size	CI-low	CI-high	Power (%)
47	-1,379	-3,901	1,463	26,890
48	0,280	-1,475	2,062	9,130
49	1,208	-0,160	2,678	48,630
50	2,559	1,373	3,786	99,310
51	1,151	0,146	2,155	72,350
52	2,367	1,448	3,296	99,920
53	1,364	0,492	2,230	92,060
54	1,759	0,840	2,685	97,700
55	1,125	0,249	1,976	82,910

56	1,712	0,854	2,616	98,170
57	1,251	0,434	2,096	89,540
58	1,379	0,569	2,193	94,510
59	1,023	0,226	1,832	79,450
60	1,740	0,967	2,501	99,600
61	1,883	1,128	2,648	99,930
62	1,573	0,785	2,346	98,990
63	1,762	1,027	2,497	99,840
64	1,549	0,806	2,262	99,310
65	1,656	0,929	2,389	99,660
66	1,380	0,665	2,051	98,760
67	1,795	1,129	2,468	99,980
68	1,307	0,628	2,004	98,180
69	1,360	0,683	2,045	98,460
70	0,786	0,029	1,529	65,470
71	0,951	0,240	1,669	83,410
72	1,321	0,507	2,115	94,900
73	1,012	0,139	1,849	76,580
74	0,945	-0,073	1,908	60,710
75	1,690	0,664	2,664	93,430
76	0,458	-0,823	1,725	17,650
77	0,358	-1,086	1,819	12,250
78	-0,059	-1,753	1,697	5,880
79	0,177	-1,907	2,091	6,790
Multiplex clustering coefficient				
Age	MD effect-size	CI-low	CI-high	Power (%)
47	-2,646	-5,929	0,964	43,750
48	-0,391	-2,557	1,885	9,400
49	0,778	-1,057	2,656	20,710
50	1,342	-0,166	2,912	54,900
51	0,232	-1,021	1,503	9,870
52	1,027	-0,121	2,162	51,850
53	0,090	-0,999	1,148	6,970
54	0,476	-0,656	1,625	20,390
55	0,552	-0,548	1,669	25,670
56	0,445	-0,647	1,571	19,220
57	-0,082	-1,108	0,958	6,510
58	0,403	-0,641	1,460	19,190
59	0,297	-0,692	1,308	14,180
60	0,708	-0,262	1,714	41,310
61	0,910	-0,059	1,848	59,890
62	1,145	0,197	2,135	76,280
63	1,011	0,109	1,963	69,990
64	0,442	-0,483	1,331	25,950

65	-0,036	-0,940	0,843	5,640
66	0,478	-0,365	1,350	28,200
67	0,476	-0,358	1,310	28,970
68	0,422	-0,395	1,246	26,290
69	-0,233	-1,095	0,597	12,880
70	-0,127	-0,996	0,793	8,700
71	-0,523	-1,439	0,389	29,190
72	0,409	-0,555	1,376	21,010
73	-0,229	-1,267	0,808	11,330
74	-0,319	-1,563	0,833	13,040
75	0,163	-1,055	1,321	8,070
76	-1,582	-3,098	-0,094	68,580
77	-0,714	-2,434	0,987	20,440
78	-0,555	-2,761	1,556	13,490
79	-1,072	-3,308	1,036	24,330

Multiplex participation coefficient

Age	MD effect-size	CI-low	CI-high	Power (%)
47	0,384	-0,097	0,829	49,520
48	0,178	-0,106	0,443	36,310
49	0,474	0,232	0,717	98,220
50	0,223	0,019	0,439	66,950
51	0,447	0,284	0,609	99,970
52	0,580	0,422	0,731	100,000
53	0,405	0,266	0,545	99,990
54	0,385	0,240	0,525	99,980
55	0,376	0,233	0,522	99,980
56	0,318	0,177	0,458	99,630
57	0,434	0,289	0,579	100,000
58	0,401	0,268	0,537	100,000
59	0,395	0,257	0,527	99,990
60	0,415	0,280	0,545	100,000
61	0,265	0,147	0,379	99,770
62	0,289	0,162	0,414	99,730
63	0,305	0,184	0,427	99,970
64	0,350	0,233	0,469	99,980
65	0,345	0,231	0,457	100,000
66	0,258	0,144	0,370	99,540
67	0,284	0,170	0,394	99,960
68	0,317	0,201	0,432	100,000
69	0,367	0,254	0,480	100,000
70	0,327	0,216	0,436	100,000
71	0,257	0,134	0,375	99,550
72	0,277	0,143	0,412	99,180
73	0,225	0,088	0,358	94,680

74	0,254	0,101	0,406	94,650
75	0,339	0,162	0,511	98,630
76	0,233	0,043	0,427	77,490
77	0,260	0,044	0,482	74,800
78	0,336	0,086	0,591	85,050
79	0,389	0,048	0,714	75,020

Multilayer clustering coefficient

Age	MD effect-size	CI-low	CI-high	Power
47	0,773	0,367	1,170	97,130
48	0,045	-0,237	0,330	9,400
49	0,211	-0,024	0,449	53,560
50	0,206	0,014	0,399	66,520
51	0,428	0,254	0,598	99,860
52	0,381	0,227	0,528	99,950
53	0,306	0,164	0,445	99,270
54	0,270	0,119	0,411	97,600
55	0,206	0,067	0,352	87,330
56	0,187	0,037	0,333	80,190
57	0,331	0,186	0,478	99,800
58	0,371	0,235	0,503	99,990
59	0,321	0,187	0,463	99,830
60	0,299	0,165	0,430	99,710
61	0,146	0,022	0,272	73,420
62	0,192	0,059	0,319	88,110
63	0,267	0,149	0,383	99,590
64	0,120	0,004	0,240	63,910
65	0,225	0,100	0,346	97,020
66	0,125	0,007	0,244	66,290
67	0,227	0,114	0,342	98,530
68	0,259	0,144	0,373	99,710
69	0,280	0,157	0,403	99,700
70	0,273	0,158	0,390	99,840
71	0,228	0,104	0,353	97,040
72	0,254	0,117	0,393	97,870
73	0,226	0,077	0,373	90,550
74	0,228	0,069	0,393	85,790
75	0,331	0,141	0,515	96,790
76	0,448	0,248	0,667	99,620
77	0,097	-0,128	0,331	22,010
78	0,362	0,100	0,649	83,930
79	0,356	0,048	0,686	69,610

Multilayer global efficiency

Age	MD effect-size	CI-low	CI-high	Power
47	0,018	-0,636	0,710	5,600
48	-0,147	-0,677	0,363	13,620
49	0,273	-0,148	0,687	33,190
50	0,738	0,371	1,105	98,770
51	0,526	0,215	0,830	94,970
52	0,668	0,399	0,944	99,880
53	0,477	0,224	0,736	97,070
54	0,505	0,228	0,772	97,510
55	0,270	0,003	0,524	64,310
56	0,319	0,067	0,572	75,570
57	0,388	0,145	0,631	93,190
58	0,493	0,243	0,736	98,900
59	0,259	0,024	0,496	68,490
60	0,423	0,193	0,651	97,130
61	0,257	0,026	0,487	71,950
62	0,414	0,183	0,647	96,390
63	0,416	0,199	0,626	98,260
64	0,163	-0,057	0,387	41,080
65	0,315	0,093	0,527	88,260
66	0,210	-0,006	0,426	62,740
67	0,262	0,055	0,466	81,340
68	0,283	0,077	0,488	84,050
69	0,150	-0,060	0,359	40,770
70	0,140	-0,072	0,352	35,960
71	0,105	-0,115	0,324	23,660
72	0,165	-0,067	0,394	38,240
73	0,212	-0,048	0,464	49,220
74	0,065	-0,231	0,357	11,430
75	0,316	0,007	0,617	63,680
76	-0,064	-0,421	0,286	9,880
77	-0,210	-0,627	0,218	24,630
78	0,053	-0,452	0,575	7,700
79	-0,102	-0,752	0,548	9,380
