# Supplementary material for: Sex differences in multilayer functional network topology over the course of aging in 37543 UK Biobank participants
Source: Netw Neurosci. 2023 Jan 1;7(1):351–76. doi: 10.1162/netn_a_00286 (PMC10275214; doi:10.1162/netn_a_00286)
Supplement: Supplementary file 5 [file netn-7-1-351-s005.pdf]

Mijalkov, M., Veréb, D., Jamialahmadi, O., Canal-Garcia, A., Gómez-Ruiz, E., Vidal-Piñeiro, D., Romeo, S., Volpe, G. & Pereira, J. (2023). Supporting information for "Sex differences in multilayer functional network topology over the course of aging in 37543 UK Biobank participants. *Network Neuroscience*, *7*(1), 351–376. https://doi.org/10.1162/netn_a_00286

# Sex differences in multilayer functional network topology over the course of aging in 37543 UK Biobank participants

## Supplementary information

## Statistical comparisons

Mite Mijalkov,[1,*] Dániel Veréb,[1] Anna Canal Garcia,[1] Emiliano Gomez Ruiz,[2] Oveis Jamialahmadi,[3] Stefano Romeo,[3] Giovanni Volpe,[2] and Joana B. Pereira[1,4,*]

[1]*Department of Neurobiology, Care Sciences and Society, Karolinska Institutet, Stockholm, Sweden*

[2]*Department of Physics, Goteborg University, Goteborg, Sweden*

[3]*Department of Molecular and Clinical Medicine, Goteborg University, Goteborg, Sweden*

[4]*Memory Research Unit, Department of Clinical Sciences Malmö, Lund University, Lund, Sweden*

[*] Corresponding authors: Email: mite.mijalkov@ki.se // joana.pereira@ki.se. Address: KI, Dept. NVS, division of clinical geriatrics, Neo 7th floor, Blickagången 16, 141 83 Huddinge, Sweden.

Table S1: Report of statistics for all comparisons presented in the main text.

| Age | Mean-Women | S.E.-Women | Mean-Men | S.E.-Men | Difference | p-value | CI-low | CI-high |
|---|---|---|---|---|---|---|---|---|
| Average whole brain connectivity | | | | | | | | |
| Age | Mean-Women | S.E.-Women | Mean-Men | S.E.-Men | Difference | p-value | CI-low | CI-high |
| 47 | 2,830 | 2,488 | -2,913 | 2,488 | 5,743 | 0,150 | -6,454 | 6,576 |
| 48 | -3,842 | 1,794 | -3,127 | 1,794 | -0,715 | 0,786 | -4,401 | 4,445 |
| 49 | 4,991 | 1,583 | 0,907 | 1,583 | 4,084 | 0,063 | -3,671 | 3,569 |
| 50 | 3,800 | 1,305 | -0,938 | 1,305 | 4,737 | 0,015 | -3,318 | 3,139 |
| 51 | 5,771 | 1,123 | -2,631 | 1,123 | 8,402 | <0,001 | -2,768 | 2,883 |
| 52 | 5,674 | 1,028 | -0,951 | 1,028 | 6,625 | <0,001 | -2,417 | 2,503 |
| 53 | 5,834 | 1,015 | -0,414 | 1,015 | 6,248 | <0,001 | -2,423 | 2,369 |
| 54 | 5,720 | 0,933 | 1,583 | 0,933 | 4,137 | 0,004 | -2,292 | 2,287 |
| 55 | 6,037 | 0,912 | 2,984 | 0,912 | 3,053 | 0,032 | -2,345 | 2,393 |
| 56 | 5,373 | 0,932 | 2,107 | 0,932 | 3,266 | 0,019 | -2,319 | 2,312 |
| 57 | 4,996 | 0,957 | 0,008 | 0,957 | 4,988 | <0,001 | -2,355 | 2,339 |
| 58 | 7,791 | 0,902 | 1,432 | 0,902 | 6,359 | <0,001 | -2,215 | 2,241 |
| 59 | 7,473 | 0,910 | 1,715 | 0,910 | 5,758 | <0,001 | -2,197 | 2,222 |
| 60 | 6,750 | 0,820 | 1,183 | 0,820 | 5,567 | <0,001 | -2,062 | 2,073 |
| 61 | 4,717 | 0,777 | 2,277 | 0,777 | 2,440 | 0,040 | -1,905 | 1,969 |
| 62 | 6,214 | 0,827 | 1,804 | 0,827 | 4,410 | <0,001 | -1,989 | 2,037 |
| 63 | 6,290 | 0,774 | 2,096 | 0,774 | 4,194 | <0,001 | -1,904 | 1,882 |
| 64 | 4,857 | 0,787 | 2,952 | 0,787 | 1,905 | 0,099 | -1,923 | 1,889 |
| 65 | 6,729 | 0,779 | 3,215 | 0,779 | 3,514 | 0,002 | -1,902 | 1,892 |
| 66 | 5,665 | 0,816 | 2,905 | 0,816 | 2,760 | 0,016 | -1,842 | 1,902 |
| 67 | 5,656 | 0,815 | 2,375 | 0,815 | 3,281 | 0,003 | -1,911 | 1,854 |
| 68 | 7,526 | 0,760 | 3,906 | 0,760 | 3,620 | 0,001 | -1,790 | 1,815 |
| 69 | 6,960 | 0,820 | 4,403 | 0,820 | 2,557 | 0,022 | -1,889 | 1,833 |
| 70 | 6,601 | 0,813 | 2,420 | 0,813 | 4,181 | <0,001 | -1,809 | 1,820 |
| 71 | 6,882 | 0,856 | 4,371 | 0,856 | 2,511 | 0,027 | -1,909 | 1,911 |
| 72 | 7,888 | 0,961 | 5,874 | 0,961 | 2,015 | 0,128 | -2,182 | 2,173 |
| 73 | 6,601 | 0,960 | 3,744 | 0,960 | 2,857 | 0,035 | -2,254 | 2,276 |
| 74 | 6,926 | 1,185 | 5,330 | 1,185 | 1,596 | 0,309 | -2,567 | 2,587 |
| 75 | 8,186 | 1,353 | 4,055 | 1,353 | 4,131 | 0,013 | -2,749 | 2,718 |
| 76 | 7,268 | 1,464 | 5,252 | 1,464 | 2,015 | 0,290 | -3,136 | 3,129 |
| 77 | 5,670 | 1,622 | 7,995 | 1,622 | -2,325 | 0,280 | -3,596 | 3,652 |
| 78 | 10,368 | 1,818 | 5,377 | 1,818 | 4,990 | 0,046 | -4,089 | 4,150 |
| 79 | 8,522 | 2,686 | 6,495 | 2,686 | 2,027 | 0,519 | -5,127 | 5,091 |
| Average negative connectivity | | | | | | | | |
| Age | Mean-Women | S.E.-Women | Mean-Men | S.E.-Men | Difference | p-value | CI-low | CI-high |
| 47 | -165,555 | 2,722 | -158,545 | 2,722 | -7,010 | 0,123 | -7,432 | 7,432 |
| 48 | -163,014 | 1,875 | -163,330 | 1,875 | 0,317 | 0,918 | -4,618 | 4,670 |
| 49 | -163,821 | 1,651 | -165,006 | 1,651 | 1,185 | 0,626 | -3,989 | 4,020 |

| | | | | | | | | |
|---|---|---|---|---|---|---|---|---|
| 50 | -161,818 | 1,278 | -167,049 | 1,278 | 5,230 | 0,013 | -3,346 | 3,439 |
| 51 | -163,473 | 1,086 | -163,448 | 1,086 | -0,025 | 0,990 | -2,686 | 2,697 |
| 52 | -163,545 | 1,075 | -166,561 | 1,075 | 3,016 | 0,053 | -2,568 | 2,583 |
| 53 | -164,170 | 1,028 | -164,437 | 1,028 | 0,267 | 0,849 | -2,393 | 2,422 |
| 54 | -164,549 | 0,999 | -167,084 | 0,999 | 2,535 | 0,108 | -2,557 | 2,596 |
| 55 | -164,991 | 0,906 | -166,042 | 0,906 | 1,051 | 0,473 | -2,343 | 2,355 |
| 56 | -163,571 | 0,944 | -166,168 | 0,944 | 2,596 | 0,076 | -2,429 | 2,406 |
| 57 | -163,300 | 0,923 | -163,213 | 0,923 | -0,088 | 0,946 | -2,283 | 2,320 |
| 58 | -163,870 | 0,881 | -165,255 | 0,881 | 1,385 | 0,318 | -2,244 | 2,235 |
| 59 | -164,582 | 0,874 | -165,324 | 0,874 | 0,742 | 0,560 | -2,248 | 2,149 |
| 60 | -163,490 | 0,860 | -165,547 | 0,860 | 2,057 | 0,118 | -2,178 | 2,159 |
| 61 | -162,679 | 0,797 | -166,649 | 0,797 | 3,971 | 0,001 | -2,011 | 2,082 |
| 62 | -163,476 | 0,825 | -166,374 | 0,825 | 2,898 | 0,026 | -2,100 | 2,126 |
| 63 | -163,127 | 0,801 | -166,175 | 0,801 | 3,047 | 0,014 | -1,998 | 1,958 |
| 64 | -163,386 | 0,795 | -166,074 | 0,795 | 2,688 | 0,024 | -1,948 | 1,961 |
| 65 | -164,530 | 0,815 | -166,230 | 0,815 | 1,700 | 0,158 | -2,013 | 2,003 |
| 66 | -163,014 | 0,795 | -165,544 | 0,795 | 2,531 | 0,030 | -1,895 | 1,948 |
| 67 | -163,819 | 0,769 | -166,643 | 0,769 | 2,825 | 0,012 | -1,841 | 1,840 |
| 68 | -163,344 | 0,749 | -165,039 | 0,749 | 1,695 | 0,133 | -1,803 | 1,847 |
| 69 | -165,204 | 0,837 | -165,681 | 0,837 | 0,477 | 0,680 | -1,878 | 1,918 |
| 70 | -167,085 | 0,899 | -166,943 | 0,899 | -0,141 | 0,893 | -2,031 | 2,065 |
| 71 | -166,369 | 0,892 | -166,978 | 0,892 | 0,610 | 0,620 | -2,030 | 2,058 |
| 72 | -164,464 | 0,917 | -166,317 | 0,917 | 1,853 | 0,152 | -2,140 | 2,105 |
| 73 | -165,236 | 1,002 | -166,718 | 1,002 | 1,482 | 0,282 | -2,299 | 2,267 |
| 74 | -165,892 | 1,277 | -166,543 | 1,277 | 0,651 | 0,699 | -2,676 | 2,685 |
| 75 | -165,190 | 1,195 | -166,910 | 1,195 | 1,720 | 0,310 | -2,812 | 2,757 |
| 76 | -167,956 | 1,632 | -167,506 | 1,632 | -0,450 | 0,828 | -3,363 | 3,312 |
| 77 | -164,967 | 1,711 | -166,407 | 1,711 | 1,440 | 0,536 | -3,741 | 3,812 |
| 78 | -170,742 | 2,324 | -168,289 | 2,324 | -2,453 | 0,389 | -4,732 | 4,748 |
| 79 | -167,733 | 2,442 | -165,853 | 2,442 | -1,880 | 0,564 | -5,231 | 5,125 |

Average positive connectivity

| Age | Mean-Women | S.E.-Women | Mean-Men | S.E.-Men | Difference | p-value | CI-low | CI-high |
|---|---|---|---|---|---|---|---|---|
| 47 | 182,061 | 3,143 | 170,087 | 3,143 | 11,973 | 0,018 | -8,412 | 8,519 |
| 48 | 174,932 | 2,255 | 174,285 | 2,255 | 0,647 | 0,850 | -5,727 | 5,619 |
| 49 | 178,146 | 2,038 | 180,170 | 2,038 | -2,024 | 0,508 | -4,929 | 4,831 |
| 50 | 176,054 | 1,594 | 180,367 | 1,594 | -4,312 | 0,083 | -4,148 | 3,970 |
| 51 | 177,076 | 1,370 | 177,552 | 1,370 | -0,476 | 0,814 | -3,404 | 3,449 |
| 52 | 177,366 | 1,286 | 180,739 | 1,286 | -3,372 | 0,071 | -3,083 | 3,057 |
| 53 | 178,594 | 1,233 | 179,039 | 1,233 | -0,445 | 0,798 | -2,932 | 2,926 |
| 54 | 179,169 | 1,169 | 182,258 | 1,169 | -3,089 | 0,090 | -3,028 | 2,984 |
| 55 | 179,540 | 1,132 | 182,184 | 1,132 | -2,644 | 0,136 | -2,915 | 2,859 |
| 56 | 177,770 | 1,153 | 181,819 | 1,153 | -4,049 | 0,023 | -2,956 | 2,921 |
| 57 | 177,405 | 1,164 | 177,375 | 1,164 | 0,029 | 0,981 | -2,909 | 2,892 |
| 58 | 178,147 | 1,100 | 179,376 | 1,100 | -1,229 | 0,458 | -2,757 | 2,729 |

| | | | | | | | | |
|---|---|---|---|---|---|---|---|---|
| 59 | 178,922 | 1,099 | 180,706 | 1,099 | -1,784 | 0,291 | -2,734 | 2,734 |
| 60 | 177,935 | 1,068 | 181,048 | 1,068 | -3,113 | 0,061 | -2,713 | 2,702 |
| 61 | 177,098 | 0,997 | 182,019 | 0,997 | -4,921 | 0,001 | -2,517 | 2,508 |
| 62 | 178,106 | 1,024 | 182,013 | 1,024 | -3,907 | 0,014 | -2,628 | 2,590 |
| 63 | 178,378 | 1,000 | 180,439 | 1,000 | -2,061 | 0,164 | -2,387 | 2,447 |
| 64 | 177,750 | 0,998 | 180,781 | 0,998 | -3,031 | 0,038 | -2,368 | 2,435 |
| 65 | 179,947 | 1,001 | 182,089 | 1,001 | -2,141 | 0,141 | -2,412 | 2,373 |
| 66 | 177,670 | 0,967 | 180,692 | 0,967 | -3,022 | 0,030 | -2,291 | 2,343 |
| 67 | 178,676 | 0,961 | 181,597 | 0,961 | -2,921 | 0,031 | -2,261 | 2,246 |
| 68 | 178,281 | 0,916 | 179,541 | 0,916 | -1,260 | 0,348 | -2,163 | 2,141 |
| 69 | 179,433 | 1,022 | 180,862 | 1,022 | -1,430 | 0,316 | -2,332 | 2,359 |
| 70 | 182,825 | 1,117 | 182,307 | 1,117 | 0,517 | 0,734 | -2,469 | 2,477 |
| 71 | 182,124 | 1,097 | 182,026 | 1,097 | 0,099 | 0,953 | -2,425 | 2,481 |
| 72 | 179,587 | 1,190 | 181,634 | 1,190 | -2,047 | 0,195 | -2,598 | 2,645 |
| 73 | 180,988 | 1,254 | 180,637 | 1,254 | 0,351 | 0,843 | -2,810 | 2,837 |
| 74 | 180,343 | 1,473 | 182,040 | 1,473 | -1,696 | 0,384 | -3,199 | 3,199 |
| 75 | 178,858 | 1,433 | 180,828 | 1,433 | -1,970 | 0,330 | -3,302 | 3,399 |
| 76 | 184,302 | 2,006 | 182,024 | 2,006 | 2,278 | 0,348 | -3,979 | 3,990 |
| 77 | 181,011 | 2,036 | 182,377 | 2,036 | -1,366 | 0,618 | -4,442 | 4,404 |
| 78 | 185,909 | 2,778 | 183,609 | 2,778 | 2,300 | 0,501 | -5,431 | 5,513 |
| 79 | 184,547 | 2,682 | 181,974 | 2,682 | 2,573 | 0,475 | -5,889 | 5,765 |

Number of negative connections

| Age | Mean-Women | S.E.-Women | Mean-Men | S.E.-Men | Difference | p-value | CI-low | CI-high |
|---|---|---|---|---|---|---|---|---|
| 47 | 1404,789 | 17,478 | 1438,118 | 17,478 | -33,329 | 0,252 | -47,772 | 47,367 |
| 48 | 1438,782 | 12,205 | 1428,233 | 12,205 | 10,549 | 0,555 | -29,247 | 29,474 |
| 49 | 1378,811 | 11,249 | 1415,718 | 11,249 | -36,907 | 0,013 | -24,583 | 24,453 |
| 50 | 1387,199 | 9,353 | 1421,288 | 9,353 | -34,089 | 0,009 | -21,685 | 22,335 |
| 51 | 1373,107 | 7,699 | 1438,219 | 7,699 | -65,111 | <0,001 | -19,498 | 19,248 |
| 52 | 1371,490 | 7,167 | 1426,677 | 7,167 | -55,188 | <0,001 | -16,943 | 16,987 |
| 53 | 1375,077 | 6,839 | 1422,318 | 6,839 | -47,241 | <0,001 | -16,320 | 16,109 |
| 54 | 1375,497 | 6,221 | 1411,115 | 6,221 | -35,618 | <0,001 | -15,573 | 15,470 |
| 55 | 1373,960 | 6,034 | 1402,702 | 6,034 | -28,742 | 0,002 | -15,652 | 15,205 |
| 56 | 1375,774 | 6,498 | 1408,592 | 6,498 | -32,818 | 0,001 | -15,843 | 16,067 |
| 57 | 1382,107 | 6,309 | 1419,186 | 6,309 | -37,079 | <0,001 | -15,635 | 15,439 |
| 58 | 1359,398 | 6,116 | 1405,680 | 6,116 | -46,282 | <0,001 | -14,729 | 14,865 |
| 59 | 1363,671 | 6,208 | 1409,643 | 6,208 | -45,972 | <0,001 | -14,836 | 14,713 |
| 60 | 1368,956 | 5,454 | 1414,555 | 5,454 | -45,599 | <0,001 | -13,716 | 13,350 |
| 61 | 1383,000 | 5,371 | 1403,472 | 5,371 | -20,472 | 0,008 | -12,672 | 13,219 |
| 62 | 1372,227 | 5,702 | 1408,672 | 5,702 | -36,445 | <0,001 | -13,943 | 13,610 |
| 63 | 1373,636 | 5,306 | 1401,142 | 5,306 | -27,506 | <0,001 | -12,683 | 13,150 |
| 64 | 1382,055 | 5,314 | 1395,523 | 5,314 | -13,468 | 0,073 | -12,278 | 12,371 |
| 65 | 1372,466 | 5,330 | 1399,192 | 5,330 | -26,726 | 0,001 | -12,527 | 12,644 |
| 66 | 1376,612 | 5,581 | 1398,586 | 5,581 | -21,973 | 0,005 | -13,068 | 12,560 |
| 67 | 1377,711 | 5,488 | 1401,444 | 5,488 | -23,733 | 0,002 | -12,630 | 11,850 |

| | | | | | | | | |
|---|---|---|---|---|---|---|---|---|
| 68 | 1362,899 | 5,227 | 1389,917 | 5,227 | -27,018 | <0,001 | -12,366 | 12,260 |
| 69 | 1366,153 | 5,420 | 1386,726 | 5,420 | -20,573 | 0,006 | -12,282 | 12,151 |
| 70 | 1372,619 | 5,375 | 1401,111 | 5,375 | -28,492 | <0,001 | -12,239 | 12,107 |
| 71 | 1371,346 | 5,689 | 1385,948 | 5,689 | -14,603 | 0,052 | -12,331 | 12,672 |
| 72 | 1361,806 | 6,408 | 1377,337 | 6,408 | -15,531 | 0,068 | -14,058 | 13,961 |
| 73 | 1372,264 | 6,387 | 1386,618 | 6,387 | -14,354 | 0,111 | -14,938 | 14,930 |
| 74 | 1365,460 | 8,003 | 1382,539 | 8,003 | -17,078 | 0,097 | -17,072 | 16,736 |
| 75 | 1352,408 | 9,137 | 1385,511 | 9,137 | -33,102 | 0,003 | -19,151 | 19,069 |
| 76 | 1370,228 | 9,491 | 1376,444 | 9,491 | -6,217 | 0,624 | -20,741 | 20,316 |
| 77 | 1382,164 | 10,178 | 1364,136 | 10,178 | 18,028 | 0,201 | -23,293 | 23,128 |
| 78 | 1343,772 | 12,584 | 1377,602 | 12,584 | -33,830 | 0,043 | -27,079 | 27,509 |
| 79 | 1362,816 | 17,262 | 1371,822 | 17,262 | -9,006 | 0,668 | -33,484 | 32,669 |

Clustering coefficient - positive network

| Age | Mean-Women | S.E.-Women | Mean-Men | S.E.-Men | Difference | p-value | CI-low | CI-high |
|---|---|---|---|---|---|---|---|---|
| 47 | 67,470 | 2,020 | 57,754 | 2,020 | 9,716 | 0,004 | -5,557 | 5,632 |
| 48 | 61,477 | 1,409 | 59,844 | 1,409 | 1,632 | 0,444 | -3,436 | 3,479 |
| 49 | 64,327 | 1,246 | 63,185 | 1,246 | 1,142 | 0,517 | -2,946 | 2,878 |
| 50 | 62,331 | 0,976 | 63,065 | 0,976 | -0,735 | 0,616 | -2,462 | 2,480 |
| 51 | 64,318 | 0,855 | 60,856 | 0,855 | 3,462 | 0,008 | -2,114 | 2,186 |
| 52 | 64,210 | 0,792 | 63,572 | 0,792 | 0,638 | 0,575 | -1,919 | 1,899 |
| 53 | 64,362 | 0,744 | 62,201 | 0,744 | 2,161 | 0,051 | -1,818 | 1,800 |
| 54 | 65,048 | 0,738 | 64,870 | 0,738 | 0,177 | 0,879 | -1,894 | 1,911 |
| 55 | 64,842 | 0,722 | 65,247 | 0,722 | -0,404 | 0,726 | -1,865 | 1,806 |
| 56 | 63,775 | 0,725 | 64,018 | 0,725 | -0,243 | 0,833 | -1,834 | 1,825 |
| 57 | 63,094 | 0,716 | 61,110 | 0,716 | 1,984 | 0,063 | -1,772 | 1,762 |
| 58 | 65,142 | 0,691 | 62,839 | 0,691 | 2,302 | 0,032 | -1,767 | 1,748 |
| 59 | 65,359 | 0,718 | 63,505 | 0,718 | 1,854 | 0,080 | -1,733 | 1,742 |
| 60 | 64,303 | 0,674 | 63,544 | 0,674 | 0,759 | 0,469 | -1,667 | 1,707 |
| 61 | 63,768 | 0,626 | 64,155 | 0,626 | -0,388 | 0,683 | -1,571 | 1,544 |
| 62 | 64,710 | 0,649 | 64,637 | 0,649 | 0,073 | 0,928 | -1,608 | 1,596 |
| 63 | 64,119 | 0,620 | 63,716 | 0,620 | 0,403 | 0,658 | -1,550 | 1,529 |
| 64 | 64,070 | 0,644 | 64,439 | 0,644 | -0,369 | 0,701 | -1,556 | 1,530 |
| 65 | 65,070 | 0,629 | 64,717 | 0,629 | 0,353 | 0,698 | -1,538 | 1,515 |
| 66 | 63,299 | 0,615 | 63,525 | 0,615 | -0,226 | 0,794 | -1,466 | 1,479 |
| 67 | 64,853 | 0,639 | 63,589 | 0,639 | 1,264 | 0,146 | -1,422 | 1,434 |
| 68 | 64,501 | 0,603 | 63,731 | 0,603 | 0,771 | 0,375 | -1,448 | 1,448 |
| 69 | 65,473 | 0,679 | 64,141 | 0,679 | 1,332 | 0,150 | -1,517 | 1,521 |
| 70 | 66,650 | 0,679 | 64,240 | 0,679 | 2,411 | 0,010 | -1,557 | 1,539 |
| 71 | 66,762 | 0,693 | 64,981 | 0,693 | 1,782 | 0,062 | -1,578 | 1,559 |
| 72 | 65,548 | 0,771 | 64,867 | 0,771 | 0,681 | 0,507 | -1,722 | 1,691 |
| 73 | 65,312 | 0,817 | 63,507 | 0,817 | 1,804 | 0,105 | -1,863 | 1,842 |
| 74 | 65,555 | 0,958 | 64,299 | 0,958 | 1,255 | 0,323 | -2,114 | 2,104 |
| 75 | 65,769 | 1,022 | 63,230 | 1,022 | 2,538 | 0,067 | -2,234 | 2,302 |
| 76 | 68,721 | 1,315 | 63,301 | 1,315 | 5,420 | <0,001 | -2,550 | 2,582 |

| | | | | | | | | |
|---|---|---|---|---|---|---|---|---|
| 77 | 65,646 | 1,300 | 64,791 | 1,300 | 0,855 | 0,647 | -2,870 | 2,957 |
| 78 | 68,986 | 1,722 | 65,133 | 1,722 | 3,852 | 0,080 | -3,557 | 3,647 |
| 79 | 67,840 | 1,753 | 63,978 | 1,753 | 3,862 | 0,098 | -3,780 | 3,865 |

Clustering coefficient - negative network

| Age | Mean-Women | S.E.-Women | Mean-Men | S.E.-Men | Difference | p-value | CI-low | CI-high |
|---|---|---|---|---|---|---|---|---|
| 47 | 2,083 | 0,223 | 2,750 | 0,223 | -0,667 | 0,053 | -0,565 | 0,573 |
| 48 | 2,529 | 0,155 | 2,785 | 0,155 | -0,256 | 0,290 | -0,402 | 0,401 |
| 49 | 2,486 | 0,145 | 2,328 | 0,145 | 0,157 | 0,407 | -0,317 | 0,311 |
| 50 | 2,308 | 0,096 | 2,281 | 0,096 | 0,027 | 0,847 | -0,231 | 0,232 |
| 51 | 2,371 | 0,081 | 2,394 | 0,081 | -0,022 | 0,848 | -0,202 | 0,200 |
| 52 | 2,390 | 0,080 | 2,293 | 0,080 | 0,097 | 0,382 | -0,182 | 0,184 |
| 53 | 2,454 | 0,074 | 2,350 | 0,074 | 0,104 | 0,308 | -0,175 | 0,169 |
| 54 | 2,344 | 0,071 | 2,488 | 0,071 | -0,144 | 0,177 | -0,174 | 0,171 |
| 55 | 2,378 | 0,068 | 2,444 | 0,068 | -0,066 | 0,532 | -0,178 | 0,179 |
| 56 | 2,478 | 0,072 | 2,475 | 0,072 | 0,003 | 0,974 | -0,183 | 0,179 |
| 57 | 2,462 | 0,067 | 2,511 | 0,067 | -0,049 | 0,643 | -0,174 | 0,169 |
| 58 | 2,470 | 0,066 | 2,571 | 0,066 | -0,102 | 0,308 | -0,167 | 0,165 |
| 59 | 2,439 | 0,064 | 2,475 | 0,064 | -0,036 | 0,722 | -0,157 | 0,153 |
| 60 | 2,574 | 0,063 | 2,527 | 0,063 | 0,048 | 0,624 | -0,158 | 0,159 |
| 61 | 2,564 | 0,062 | 2,522 | 0,062 | 0,042 | 0,642 | -0,148 | 0,150 |
| 62 | 2,524 | 0,061 | 2,503 | 0,061 | 0,021 | 0,836 | -0,155 | 0,155 |
| 63 | 2,609 | 0,061 | 2,545 | 0,061 | 0,064 | 0,470 | -0,147 | 0,150 |
| 64 | 2,596 | 0,061 | 2,666 | 0,061 | -0,069 | 0,441 | -0,147 | 0,148 |
| 65 | 2,488 | 0,061 | 2,618 | 0,061 | -0,129 | 0,140 | -0,143 | 0,147 |
| 66 | 2,545 | 0,059 | 2,693 | 0,059 | -0,148 | 0,082 | -0,140 | 0,139 |
| 67 | 2,647 | 0,063 | 2,714 | 0,063 | -0,067 | 0,435 | -0,141 | 0,143 |
| 68 | 2,746 | 0,059 | 2,851 | 0,059 | -0,105 | 0,242 | -0,148 | 0,152 |
| 69 | 2,665 | 0,063 | 2,819 | 0,063 | -0,154 | 0,078 | -0,144 | 0,145 |
| 70 | 2,522 | 0,063 | 2,654 | 0,063 | -0,132 | 0,124 | -0,142 | 0,141 |
| 71 | 2,446 | 0,062 | 2,729 | 0,062 | -0,283 | 0,001 | -0,146 | 0,145 |
| 72 | 2,745 | 0,071 | 2,764 | 0,071 | -0,019 | 0,840 | -0,156 | 0,155 |
| 73 | 2,643 | 0,075 | 2,725 | 0,075 | -0,081 | 0,442 | -0,171 | 0,170 |
| 74 | 2,767 | 0,090 | 2,752 | 0,090 | 0,014 | 0,896 | -0,194 | 0,194 |
| 75 | 2,600 | 0,093 | 2,833 | 0,093 | -0,233 | 0,069 | -0,210 | 0,210 |
| 76 | 2,530 | 0,115 | 2,837 | 0,115 | -0,308 | 0,036 | -0,242 | 0,245 |
| 77 | 2,559 | 0,122 | 2,962 | 0,122 | -0,402 | 0,028 | -0,293 | 0,295 |
| 78 | 2,543 | 0,163 | 2,707 | 0,163 | -0,164 | 0,430 | -0,334 | 0,345 |
| 79 | 2,682 | 0,196 | 2,779 | 0,196 | -0,097 | 0,713 | -0,414 | 0,422 |

Global efficiency - positive network

| Age | Mean-Women | S.E.-Women | Mean-Men | S.E.-Men | Difference | p-value | CI-low | CI-high |
|---|---|---|---|---|---|---|---|---|
| 47 | 45,825 | 0,594 | 45,755 | 0,594 | 0,070 | 0,955 | -1,478 | 1,501 |
| 48 | 45,059 | 0,393 | 46,121 | 0,393 | -1,062 | 0,073 | -0,976 | 0,982 |

| | | | | | | | | |
|---|---|---|---|---|---|---|---|---|
| 49 | 45,252 | 0,296 | 46,617 | 0,296 | -1,364 | 0,004 | -0,770 | 0,762 |
| 50 | 45,600 | 0,273 | 46,477 | 0,273 | -0,878 | 0,032 | -0,676 | 0,674 |
| 51 | 45,529 | 0,225 | 46,308 | 0,225 | -0,780 | 0,021 | -0,565 | 0,569 |
| 52 | 45,594 | 0,214 | 46,851 | 0,214 | -1,257 | <0,001 | -0,531 | 0,518 |
| 53 | 45,574 | 0,200 | 46,597 | 0,200 | -1,023 | 0,001 | -0,500 | 0,490 |
| 54 | 45,866 | 0,203 | 46,731 | 0,203 | -0,866 | 0,007 | -0,522 | 0,517 |
| 55 | 46,045 | 0,181 | 46,645 | 0,181 | -0,600 | 0,035 | -0,475 | 0,477 |
| 56 | 45,808 | 0,185 | 47,110 | 0,185 | -1,302 | <0,001 | -0,469 | 0,479 |
| 57 | 45,740 | 0,193 | 46,270 | 0,193 | -0,530 | 0,067 | -0,482 | 0,468 |
| 58 | 45,848 | 0,182 | 46,814 | 0,182 | -0,966 | 0,001 | -0,470 | 0,461 |
| 59 | 45,777 | 0,171 | 46,936 | 0,171 | -1,159 | <0,001 | -0,455 | 0,451 |
| 60 | 45,705 | 0,168 | 47,166 | 0,168 | -1,461 | <0,001 | -0,442 | 0,445 |
| 61 | 45,790 | 0,163 | 47,382 | 0,163 | -1,592 | <0,001 | -0,430 | 0,430 |
| 62 | 46,003 | 0,172 | 47,108 | 0,172 | -1,105 | <0,001 | -0,430 | 0,434 |
| 63 | 46,029 | 0,164 | 47,393 | 0,164 | -1,363 | <0,001 | -0,421 | 0,418 |
| 64 | 45,856 | 0,169 | 47,430 | 0,169 | -1,574 | <0,001 | -0,421 | 0,417 |
| 65 | 46,219 | 0,167 | 47,546 | 0,167 | -1,327 | <0,001 | -0,421 | 0,427 |
| 66 | 46,304 | 0,167 | 47,447 | 0,167 | -1,143 | <0,001 | -0,411 | 0,392 |
| 67 | 46,056 | 0,156 | 47,758 | 0,156 | -1,702 | <0,001 | -0,398 | 0,386 |
| 68 | 46,235 | 0,165 | 47,358 | 0,165 | -1,123 | <0,001 | -0,404 | 0,388 |
| 69 | 46,383 | 0,165 | 47,719 | 0,165 | -1,336 | <0,001 | -0,380 | 0,395 |
| 70 | 46,781 | 0,175 | 47,708 | 0,175 | -0,927 | <0,001 | -0,407 | 0,392 |
| 71 | 46,688 | 0,180 | 47,791 | 0,180 | -1,103 | <0,001 | -0,419 | 0,411 |
| 72 | 46,537 | 0,203 | 47,736 | 0,203 | -1,199 | <0,001 | -0,480 | 0,456 |
| 73 | 46,934 | 0,225 | 47,903 | 0,225 | -0,969 | 0,001 | -0,513 | 0,505 |
| 74 | 46,598 | 0,253 | 47,884 | 0,253 | -1,286 | <0,001 | -0,558 | 0,564 |
| 75 | 46,765 | 0,273 | 48,009 | 0,273 | -1,243 | 0,001 | -0,615 | 0,604 |
| 76 | 47,119 | 0,316 | 48,263 | 0,316 | -1,144 | 0,005 | -0,648 | 0,665 |
| 77 | 46,975 | 0,375 | 48,128 | 0,375 | -1,152 | 0,018 | -0,787 | 0,799 |
| 78 | 47,991 | 0,468 | 48,400 | 0,468 | -0,409 | 0,469 | -0,938 | 0,971 |
| 79 | 47,127 | 0,484 | 48,182 | 0,484 | -1,055 | 0,131 | -1,138 | 1,146 |

Global efficiency - negative network

| Age | Mean-Women | S.E.-Women | Mean-Men | S.E.-Men | Difference | p-value | CI-low | CI-high |
|---|---|---|---|---|---|---|---|---|
| 47 | 60,483 | 0,903 | 59,103 | 0,903 | 1,379 | 0,319 | -2,250 | 2,228 |
| 48 | 59,873 | 0,595 | 60,153 | 0,595 | -0,280 | 0,759 | -1,488 | 1,474 |
| 49 | 59,588 | 0,528 | 60,796 | 0,528 | -1,208 | 0,102 | -1,206 | 1,228 |
| 50 | 58,719 | 0,411 | 61,279 | 0,411 | -2,559 | <0,001 | -1,058 | 1,023 |
| 51 | 59,315 | 0,338 | 60,466 | 0,338 | -1,151 | 0,024 | -0,836 | 0,859 |
| 52 | 59,139 | 0,341 | 61,506 | 0,341 | -2,367 | <0,001 | -0,801 | 0,808 |
| 53 | 59,305 | 0,311 | 60,669 | 0,311 | -1,364 | 0,003 | -0,751 | 0,736 |
| 54 | 59,538 | 0,315 | 61,296 | 0,315 | -1,759 | <0,001 | -0,786 | 0,782 |
| 55 | 59,574 | 0,281 | 60,699 | 0,281 | -1,125 | 0,011 | -0,724 | 0,725 |
| 56 | 59,435 | 0,301 | 61,147 | 0,301 | -1,712 | <0,001 | -0,757 | 0,723 |
| 57 | 59,073 | 0,273 | 60,325 | 0,273 | -1,251 | 0,003 | -0,712 | 0,685 |

| | | | | | | | | |
|---|---|---|---|---|---|---|---|---|
| 58 | 59,323 | 0,277 | 60,701 | 0,277 | -1,379 | 0,001 | -0,684 | 0,699 |
| 59 | 59,766 | 0,278 | 60,789 | 0,278 | -1,023 | 0,018 | -0,697 | 0,688 |
| 60 | 59,088 | 0,258 | 60,828 | 0,258 | -1,740 | <0,001 | -0,655 | 0,641 |
| 61 | 59,232 | 0,252 | 61,115 | 0,252 | -1,883 | <0,001 | -0,623 | 0,644 |
| 62 | 59,444 | 0,256 | 61,017 | 0,256 | -1,573 | <0,001 | -0,665 | 0,658 |
| 63 | 59,212 | 0,252 | 60,974 | 0,252 | -1,762 | <0,001 | -0,627 | 0,617 |
| 64 | 59,327 | 0,252 | 60,876 | 0,252 | -1,549 | <0,001 | -0,623 | 0,621 |
| 65 | 59,488 | 0,250 | 61,144 | 0,250 | -1,656 | <0,001 | -0,622 | 0,608 |
| 66 | 59,363 | 0,248 | 60,742 | 0,248 | -1,380 | <0,001 | -0,581 | 0,581 |
| 67 | 59,525 | 0,239 | 61,320 | 0,239 | -1,795 | <0,001 | -0,570 | 0,577 |
| 68 | 59,301 | 0,244 | 60,608 | 0,244 | -1,307 | <0,001 | -0,577 | 0,563 |
| 69 | 59,614 | 0,250 | 60,975 | 0,250 | -1,360 | <0,001 | -0,570 | 0,584 |
| 70 | 60,438 | 0,273 | 61,223 | 0,273 | -0,786 | 0,041 | -0,627 | 0,630 |
| 71 | 60,207 | 0,265 | 61,158 | 0,265 | -0,951 | 0,011 | -0,607 | 0,595 |
| 72 | 59,455 | 0,287 | 60,776 | 0,287 | -1,321 | 0,001 | -0,669 | 0,652 |
| 73 | 59,904 | 0,319 | 60,916 | 0,319 | -1,012 | 0,022 | -0,717 | 0,718 |
| 74 | 59,878 | 0,391 | 60,823 | 0,391 | -0,945 | 0,057 | -0,816 | 0,823 |
| 75 | 59,294 | 0,381 | 60,983 | 0,381 | -1,690 | 0,001 | -0,869 | 0,861 |
| 76 | 60,583 | 0,496 | 61,041 | 0,496 | -0,458 | 0,471 | -1,031 | 1,036 |
| 77 | 60,193 | 0,559 | 60,551 | 0,559 | -0,358 | 0,598 | -1,176 | 1,237 |
| 78 | 61,213 | 0,669 | 61,153 | 0,669 | 0,059 | 0,952 | -1,460 | 1,450 |
| 79 | 59,850 | 0,833 | 60,027 | 0,833 | -0,177 | 0,874 | -1,694 | 1,650 |

Multiplex clustering coefficient

| Age | Mean-Women | S.E.-Women | Mean-Men | S.E.-Men | Difference | p-value | CI-low | CI-high |
|---|---|---|---|---|---|---|---|---|
| 47 | 36,260 | 1,121 | 33,614 | 1,121 | 2,646 | 0,135 | -2,898 | 2,872 |
| 48 | 34,707 | 0,731 | 34,316 | 0,731 | 0,391 | 0,730 | -1,849 | 1,848 |
| 49 | 34,907 | 0,657 | 35,685 | 0,657 | -0,778 | 0,413 | -1,597 | 1,584 |
| 50 | 34,543 | 0,495 | 35,885 | 0,495 | -1,342 | 0,084 | -1,269 | 1,287 |
| 51 | 34,521 | 0,435 | 34,753 | 0,435 | -0,232 | 0,735 | -1,084 | 1,077 |
| 52 | 34,683 | 0,427 | 35,710 | 0,427 | -1,027 | 0,090 | -1,017 | 0,971 |
| 53 | 34,702 | 0,399 | 34,792 | 0,399 | -0,090 | 0,868 | -0,917 | 0,918 |
| 54 | 34,817 | 0,384 | 35,293 | 0,384 | -0,476 | 0,405 | -0,939 | 0,978 |
| 55 | 34,893 | 0,357 | 35,444 | 0,357 | -0,552 | 0,320 | -0,937 | 0,942 |
| 56 | 34,657 | 0,377 | 35,102 | 0,377 | -0,445 | 0,422 | -0,945 | 0,928 |
| 57 | 34,392 | 0,359 | 34,310 | 0,359 | 0,082 | 0,891 | -0,862 | 0,886 |
| 58 | 34,357 | 0,343 | 34,760 | 0,343 | -0,403 | 0,454 | -0,889 | 0,866 |
| 59 | 34,622 | 0,343 | 34,919 | 0,343 | -0,297 | 0,575 | -0,861 | 0,851 |
| 60 | 34,201 | 0,329 | 34,909 | 0,329 | -0,708 | 0,160 | -0,833 | 0,847 |
| 61 | 34,183 | 0,315 | 35,093 | 0,315 | -0,910 | 0,059 | -0,786 | 0,803 |
| 62 | 34,050 | 0,320 | 35,195 | 0,320 | -1,145 | 0,018 | -0,792 | 0,790 |
| 63 | 34,043 | 0,312 | 35,054 | 0,312 | -1,011 | 0,030 | -0,767 | 0,764 |
| 64 | 34,101 | 0,306 | 34,543 | 0,306 | -0,442 | 0,339 | -0,770 | 0,766 |
| 65 | 34,725 | 0,314 | 34,689 | 0,314 | 0,036 | 0,928 | -0,756 | 0,732 |
| 66 | 34,026 | 0,305 | 34,504 | 0,305 | -0,478 | 0,281 | -0,724 | 0,729 |

| | | | | | | | | |
|---|---|---|---|---|---|---|---|---|
| 67 | 34,195 | 0,302 | 34,671 | 0,302 | -0,476 | 0,258 | -0,694 | 0,713 |
| 68 | 33,730 | 0,291 | 34,152 | 0,291 | -0,422 | 0,325 | -0,711 | 0,685 |
| 69 | 34,172 | 0,312 | 33,939 | 0,312 | 0,233 | 0,595 | -0,709 | 0,711 |
| 70 | 35,256 | 0,339 | 35,129 | 0,339 | 0,127 | 0,789 | -0,746 | 0,765 |
| 71 | 34,943 | 0,341 | 34,420 | 0,341 | 0,523 | 0,250 | -0,757 | 0,739 |
| 72 | 33,966 | 0,357 | 34,374 | 0,357 | -0,409 | 0,401 | -0,802 | 0,799 |
| 73 | 34,541 | 0,381 | 34,312 | 0,381 | 0,229 | 0,659 | -0,882 | 0,855 |
| 74 | 34,345 | 0,459 | 34,026 | 0,459 | 0,319 | 0,583 | -0,978 | 0,996 |
| 75 | 34,033 | 0,448 | 34,195 | 0,448 | -0,163 | 0,793 | -1,014 | 1,015 |
| 76 | 35,747 | 0,624 | 34,165 | 0,624 | 1,582 | 0,034 | -1,215 | 1,241 |
| 77 | 34,602 | 0,648 | 33,888 | 0,648 | 0,714 | 0,386 | -1,448 | 1,383 |
| 78 | 35,647 | 0,860 | 35,092 | 0,860 | 0,555 | 0,620 | -1,796 | 1,830 |
| 79 | 35,321 | 0,855 | 34,250 | 0,855 | 1,072 | 0,344 | -1,884 | 1,893 |

Multiplex participation coefficient

| Age | Mean-Women | S.E.-Women | Mean-Men | S.E.-Men | Difference | p-value | CI-low | CI-high |
|---|---|---|---|---|---|---|---|---|
| 47 | 22,077 | 0,152 | 22,461 | 0,152 | -0,384 | 0,102 | -0,390 | 0,377 |
| 48 | 22,110 | 0,084 | 22,289 | 0,084 | -0,178 | 0,205 | -0,232 | 0,221 |
| 49 | 21,902 | 0,092 | 22,376 | 0,092 | -0,474 | <0,001 | -0,210 | 0,208 |
| 50 | 21,992 | 0,069 | 22,215 | 0,069 | -0,223 | 0,033 | -0,173 | 0,174 |
| 51 | 21,865 | 0,058 | 22,311 | 0,058 | -0,447 | <0,001 | -0,144 | 0,142 |
| 52 | 21,740 | 0,057 | 22,320 | 0,057 | -0,580 | <0,001 | -0,135 | 0,136 |
| 53 | 21,869 | 0,051 | 22,274 | 0,051 | -0,405 | <0,001 | -0,122 | 0,122 |
| 54 | 21,899 | 0,049 | 22,284 | 0,049 | -0,385 | <0,001 | -0,120 | 0,124 |
| 55 | 21,777 | 0,049 | 22,153 | 0,049 | -0,376 | <0,001 | -0,125 | 0,123 |
| 56 | 21,891 | 0,048 | 22,209 | 0,048 | -0,318 | <0,001 | -0,118 | 0,120 |
| 57 | 21,873 | 0,049 | 22,307 | 0,049 | -0,434 | <0,001 | -0,125 | 0,123 |
| 58 | 21,777 | 0,046 | 22,179 | 0,046 | -0,401 | <0,001 | -0,114 | 0,117 |
| 59 | 21,851 | 0,048 | 22,246 | 0,048 | -0,395 | <0,001 | -0,118 | 0,117 |
| 60 | 21,884 | 0,047 | 22,299 | 0,047 | -0,415 | <0,001 | -0,115 | 0,113 |
| 61 | 21,918 | 0,039 | 22,182 | 0,039 | -0,265 | <0,001 | -0,100 | 0,101 |
| 62 | 21,894 | 0,044 | 22,182 | 0,044 | -0,289 | <0,001 | -0,106 | 0,109 |
| 63 | 21,890 | 0,042 | 22,195 | 0,042 | -0,305 | <0,001 | -0,102 | 0,101 |
| 64 | 21,868 | 0,042 | 22,217 | 0,042 | -0,350 | <0,001 | -0,098 | 0,104 |
| 65 | 21,850 | 0,041 | 22,194 | 0,041 | -0,345 | <0,001 | -0,100 | 0,097 |
| 66 | 21,914 | 0,043 | 22,172 | 0,043 | -0,258 | <0,001 | -0,098 | 0,100 |
| 67 | 21,918 | 0,040 | 22,202 | 0,040 | -0,284 | <0,001 | -0,095 | 0,093 |
| 68 | 21,865 | 0,042 | 22,182 | 0,042 | -0,317 | <0,001 | -0,099 | 0,097 |
| 69 | 21,785 | 0,042 | 22,151 | 0,042 | -0,367 | <0,001 | -0,094 | 0,095 |
| 70 | 21,850 | 0,042 | 22,177 | 0,042 | -0,327 | <0,001 | -0,095 | 0,095 |
| 71 | 21,839 | 0,046 | 22,096 | 0,046 | -0,257 | <0,001 | -0,099 | 0,100 |
| 72 | 21,819 | 0,051 | 22,096 | 0,051 | -0,277 | <0,001 | -0,113 | 0,111 |
| 73 | 21,905 | 0,051 | 22,130 | 0,051 | -0,225 | 0,001 | -0,112 | 0,114 |
| 74 | 21,856 | 0,057 | 22,110 | 0,057 | -0,254 | 0,001 | -0,130 | 0,123 |
| 75 | 21,742 | 0,071 | 22,080 | 0,071 | -0,339 | <0,001 | -0,146 | 0,144 |

| | | | | | | | | |
|---|---|---|---|---|---|---|---|---|
| 76 | 21,877 | 0,074 | 22,110 | 0,074 | -0,233 | 0,017 | -0,160 | 0,162 |
| 77 | 21,818 | 0,085 | 22,078 | 0,085 | -0,260 | 0,020 | -0,183 | 0,183 |
| 78 | 21,824 | 0,102 | 22,161 | 0,102 | -0,336 | 0,006 | -0,206 | 0,206 |
| 79 | 21,667 | 0,133 | 22,056 | 0,133 | -0,389 | 0,020 | -0,272 | 0,269 |

Multilayer clustering coefficient

| Age | Mean-Women | S.E.-Women | Mean-Men | S.E.-Men | Difference | p-value | CI-low | CI-high |
|---|---|---|---|---|---|---|---|---|
| 47 | -2,095 | 0,129 | -1,323 | 0,129 | -0,773 | 0,001 | -0,354 | 0,366 |
| 48 | -1,654 | 0,093 | -1,609 | 0,093 | -0,045 | 0,768 | -0,239 | 0,234 |
| 49 | -1,958 | 0,086 | -1,747 | 0,086 | -0,211 | 0,083 | -0,201 | 0,197 |
| 50 | -1,907 | 0,066 | -1,701 | 0,066 | -0,206 | 0,037 | -0,162 | 0,163 |
| 51 | -2,024 | 0,057 | -1,596 | 0,057 | -0,428 | <0,001 | -0,149 | 0,148 |
| 52 | -2,085 | 0,053 | -1,704 | 0,053 | -0,381 | <0,001 | -0,126 | 0,126 |
| 53 | -2,015 | 0,050 | -1,709 | 0,050 | -0,306 | <0,001 | -0,120 | 0,120 |
| 54 | -2,084 | 0,049 | -1,814 | 0,049 | -0,270 | <0,001 | -0,128 | 0,124 |
| 55 | -2,113 | 0,048 | -1,906 | 0,048 | -0,206 | 0,007 | -0,123 | 0,122 |
| 56 | -2,008 | 0,049 | -1,821 | 0,049 | -0,187 | 0,014 | -0,124 | 0,122 |
| 57 | -1,978 | 0,050 | -1,647 | 0,050 | -0,331 | <0,001 | -0,123 | 0,124 |
| 58 | -2,103 | 0,047 | -1,731 | 0,047 | -0,371 | <0,001 | -0,118 | 0,116 |
| 59 | -2,113 | 0,048 | -1,791 | 0,048 | -0,321 | <0,001 | -0,118 | 0,117 |
| 60 | -2,062 | 0,045 | -1,763 | 0,045 | -0,299 | <0,001 | -0,112 | 0,113 |
| 61 | -1,981 | 0,043 | -1,836 | 0,043 | -0,146 | 0,021 | -0,103 | 0,105 |
| 62 | -2,048 | 0,045 | -1,856 | 0,045 | -0,192 | 0,004 | -0,110 | 0,110 |
| 63 | -2,071 | 0,041 | -1,803 | 0,041 | -0,267 | <0,001 | -0,099 | 0,101 |
| 64 | -1,990 | 0,041 | -1,870 | 0,041 | -0,120 | 0,043 | -0,099 | 0,097 |
| 65 | -2,096 | 0,043 | -1,871 | 0,043 | -0,225 | <0,001 | -0,103 | 0,103 |
| 66 | -1,981 | 0,042 | -1,856 | 0,042 | -0,125 | 0,038 | -0,100 | 0,100 |
| 67 | -2,041 | 0,042 | -1,814 | 0,042 | -0,227 | <0,001 | -0,097 | 0,098 |
| 68 | -2,100 | 0,042 | -1,841 | 0,042 | -0,259 | <0,001 | -0,095 | 0,097 |
| 69 | -2,159 | 0,047 | -1,879 | 0,047 | -0,280 | <0,001 | -0,105 | 0,102 |
| 70 | -2,139 | 0,045 | -1,866 | 0,045 | -0,273 | <0,001 | -0,099 | 0,099 |
| 71 | -2,157 | 0,046 | -1,929 | 0,046 | -0,228 | <0,001 | -0,105 | 0,105 |
| 72 | -2,181 | 0,052 | -1,927 | 0,052 | -0,254 | <0,001 | -0,115 | 0,117 |
| 73 | -2,150 | 0,056 | -1,925 | 0,056 | -0,226 | 0,002 | -0,123 | 0,124 |
| 74 | -2,153 | 0,061 | -1,925 | 0,061 | -0,228 | 0,006 | -0,136 | 0,140 |
| 75 | -2,236 | 0,074 | -1,906 | 0,074 | -0,331 | 0,001 | -0,155 | 0,155 |
| 76 | -2,354 | 0,085 | -1,906 | 0,085 | -0,448 | <0,001 | -0,168 | 0,170 |
| 77 | -2,135 | 0,086 | -2,038 | 0,086 | -0,097 | 0,403 | -0,193 | 0,191 |
| 78 | -2,362 | 0,109 | -2,000 | 0,109 | -0,362 | 0,009 | -0,233 | 0,228 |
| 79 | -2,423 | 0,128 | -2,068 | 0,128 | -0,356 | 0,031 | -0,268 | 0,266 |

Multilayer global efficiency

| Age | Mean-Women | S.E.-Women | Mean-Men | S.E.-Men | Difference | p-value | CI-low | CI-high |
|---|---|---|---|---|---|---|---|---|
| 47 | 4,937 | 0,243 | 4,956 | 0,243 | -0,018 | 0,955 | -0,557 | 0,559 |

| | | | | | | | | |
|---|---|---|---|---|---|---|---|---|
| 48 | 5,314 | 0,167 | 5,167 | 0,167 | 0,147 | 0,569 | -0,426 | 0,435 |
| 49 | 4,765 | 0,167 | 5,038 | 0,167 | -0,273 | 0,217 | -0,359 | 0,371 |
| 50 | 4,611 | 0,129 | 5,348 | 0,129 | -0,738 | <0,001 | -0,309 | 0,313 |
| 51 | 4,641 | 0,101 | 5,167 | 0,101 | -0,526 | 0,001 | -0,257 | 0,254 |
| 52 | 4,611 | 0,102 | 5,279 | 0,102 | -0,668 | <0,001 | -0,240 | 0,233 |
| 53 | 4,597 | 0,093 | 5,074 | 0,093 | -0,477 | <0,001 | -0,219 | 0,222 |
| 54 | 4,646 | 0,091 | 5,151 | 0,091 | -0,505 | <0,001 | -0,225 | 0,227 |
| 55 | 4,631 | 0,086 | 4,902 | 0,086 | -0,270 | 0,046 | -0,221 | 0,218 |
| 56 | 4,643 | 0,091 | 4,962 | 0,091 | -0,319 | 0,016 | -0,221 | 0,222 |
| 57 | 4,588 | 0,081 | 4,977 | 0,081 | -0,388 | 0,002 | -0,203 | 0,205 |
| 58 | 4,474 | 0,082 | 4,967 | 0,082 | -0,493 | <0,001 | -0,206 | 0,205 |
| 59 | 4,652 | 0,081 | 4,911 | 0,081 | -0,259 | 0,030 | -0,195 | 0,196 |
| 60 | 4,491 | 0,077 | 4,914 | 0,077 | -0,423 | <0,001 | -0,195 | 0,194 |
| 61 | 4,587 | 0,075 | 4,843 | 0,075 | -0,257 | 0,029 | -0,191 | 0,188 |
| 62 | 4,525 | 0,077 | 4,939 | 0,077 | -0,414 | 0,001 | -0,189 | 0,193 |
| 63 | 4,457 | 0,074 | 4,873 | 0,074 | -0,416 | <0,001 | -0,183 | 0,183 |
| 64 | 4,599 | 0,076 | 4,762 | 0,076 | -0,163 | 0,148 | -0,189 | 0,184 |
| 65 | 4,480 | 0,075 | 4,795 | 0,075 | -0,315 | 0,005 | -0,185 | 0,182 |
| 66 | 4,520 | 0,078 | 4,730 | 0,078 | -0,210 | 0,052 | -0,180 | 0,178 |
| 67 | 4,581 | 0,073 | 4,843 | 0,073 | -0,262 | 0,011 | -0,172 | 0,172 |
| 68 | 4,361 | 0,074 | 4,645 | 0,074 | -0,283 | 0,008 | -0,176 | 0,174 |
| 69 | 4,470 | 0,076 | 4,620 | 0,076 | -0,150 | 0,160 | -0,175 | 0,179 |
| 70 | 4,619 | 0,079 | 4,759 | 0,079 | -0,140 | 0,192 | -0,178 | 0,178 |
| 71 | 4,538 | 0,080 | 4,644 | 0,080 | -0,105 | 0,351 | -0,185 | 0,182 |
| 72 | 4,320 | 0,085 | 4,485 | 0,085 | -0,165 | 0,174 | -0,196 | 0,196 |
| 73 | 4,366 | 0,093 | 4,577 | 0,093 | -0,212 | 0,108 | -0,218 | 0,215 |
| 74 | 4,461 | 0,112 | 4,526 | 0,112 | -0,065 | 0,660 | -0,245 | 0,244 |
| 75 | 4,227 | 0,122 | 4,543 | 0,122 | -0,316 | 0,049 | -0,260 | 0,258 |
| 76 | 4,518 | 0,137 | 4,454 | 0,137 | 0,064 | 0,734 | -0,299 | 0,298 |
| 77 | 4,485 | 0,157 | 4,275 | 0,157 | 0,210 | 0,330 | -0,369 | 0,366 |
| 78 | 4,390 | 0,201 | 4,443 | 0,201 | -0,053 | 0,838 | -0,420 | 0,421 |
| 79 | 4,172 | 0,266 | 4,070 | 0,266 | 0,102 | 0,760 | -0,534 | 0,528 |

These statistics correspond to the results presented in Figure 2. Abbreviations: Mean-Women (S.E. – Women) and Mean-Men (S.E. – Men), average values for women and men and the corresponding error of the mean at the corresponding age; p-value, the 2-tailed p-value resulting from between-group comparison using permutation test with 10000 comparisons; CI-low and CI-high, the lower and higher bound of 95% confidence interval.

Table 2: Threshold p-values for multiple comparisons adjustment by applying false discovery rate (FDR) corrections at $q < 0.05$ using the Benjamini–Hochberg procedure.

| Functional connectivity measure | Threshold p-value |
|---|---|
| Average whole brain connectivity | 0.0316 |
| Average negative connectivity | 0.0014 |
| Average positive connectivity | 0.001 |
| Number of negative connections | 0.0128 |
| Clustering coefficient - positive network | 0.0003 |
| Clustering coefficient - negative network | 0.0012 |
| Global efficiency - positive network | 0.0352 |
| Global efficiency - negative network | 0.0237 |
| Multiplex clustering coefficient | |
| Multiplex participation coefficient | 0.0334 |
| Multilayer clustering coefficient | 0.0425 |
| Multilayer global efficiency | 0.0159 |

The 2 tailed p-values for individual comparisons were considered significant if they were smaller than the threshold p-value. The missing values in Table S2 denote that no comparisons for the corresponding measure remained significant after this correction.
