# Supplementary material for: Sex differences in multilayer functional network topology over the course of aging in 37543 UK Biobank participants
Source: Netw Neurosci. 2023 Jan 1;7(1):351–76. doi: 10.1162/netn_a_00286 (PMC10275214; doi:10.1162/netn_a_00286)
Supplement: Supplementary file 6 [file netn-7-1-351-s006.pdf]

Mijalkov, M., Veréb, D., Jamialahmadi, O., Canal-Garcia, A., Gómez-Ruiz, E., Vidal-Piñeiro, D., Romeo, S., Volpe, G. & Pereira, J. (2023). Supporting information for "Sex differences in multilayer functional network topology over the course of aging in 37543 UK Biobank participants. *Network Neuroscience*, *7*(1), 351–376. https://doi.org/10.1162/netn_a_00286

Sex differences in multilayer functional network topology over the course of aging in 37543 UK Biobank participants

Supplementary information

Test Retest

Mite Mijalkov,[1,*] Dániel Veréb,[1] Anna Canal Garcia,[1] Emiliano Gomez Ruiz,[2] Oveis Jamialahmadi,[3] Stefano Romeo,[3] Giovanni Volpe,[2] and Joana B. Pereira[1,4,*]

[1]*Department of Neurobiology, Care Sciences and Society, Karolinska Institutet, Stockholm, Sweden*

[2]*Department of Physics, Goteborg University, Goteborg, Sweden*

[3]*Department of Molecular and Clinical Medicine, Goteborg University, Goteborg, Sweden*

[4]*Memory Research Unit, Department of Clinical Sciences Malmö, Lund University, Lund, Sweden*

[*] Corresponding authors: Email: mite.mijalkov@ki.se // joana.pereira@ki.se. Address: KI, Dept. NVS, division of clinical geriatrics, Neo 7th floor, Blickagången 16, 141 83 Huddinge, Sweden.

We evaluated the test-retest reliability of the measures using the intraclass correlation coefficient (ICC) as a function of the baseline age, for the subsample of individuals who had longitudinal scans available (baseline age: 54-70 years, each age group had minimum of 50 individuals with available data, mean time between scans: 2.25 years). For the multilayer measures, when the ICCs were found to be significant for a hypothesis test under the null hypothesis that ICC is equal to zero at $p < 0.05$, they were in the range of 0.4 - 0.65, which indicates fair to good level of clinical significance (Cicchetti & Sparrow, 1981). Of note, the ICCs of the multilayer measures were higher than those of the single-layer measures and the ICCs were highest for younger individuals.

The ICC correlation coefficients were calculated under two-way random effects model, using average measurements to evaluate the consistency of agreement. The test-retest analyses used the time between baseline and longitudinal scan, as well as sex as covariates.

We would like to note that the longitudinal data we used for this analysis was available only for a smaller subset of individuals and not at all ages within the age range we considered. However, the existence of alternative datasets designed for establishing reliability and reproducibility in functional connectomics (Zuo et al., 2014) open the opportunity for future research to overcome this limitation.

Table S1: Number of individuals with longitudinal scans available at the different age groups.

Age	Sample size
54	55
55	53
56	52
58	57
59	53
60	56
61	69
62	63
63	63
64	70
65	77
66	61
67	60
69	73
70	73

Table S2: Results for the test-retest reliability of all functional measures.

Average whole brain connectivity					
Age	ICC	CI-low	CI-high	F-stat.	p-value
54	0,441	0,042	0,674	1,789	0,017
55	0,565	0,246	0,749	2,298	0,002

56	0,151	-0,479	0,513	1,178	0,281
58	0,529	0,2	0,723	2,123	0,003
59	0,088	-0,579	0,474	1,097	0,37
60	0,045	-0,629	0,44	1,047	0,432
61	0,367	-0,023	0,608	1,579	0,031
62	0,245	-0,248	0,543	1,324	0,136
63	0,504	0,18	0,7	2,017	0,003
64	0,354	-0,04	0,599	1,548	0,036
65	0,384	0,031	0,608	1,623	0,018
66	0,228	-0,286	0,537	1,296	0,159
67	0,157	-0,411	0,496	1,186	0,257
69	0,374	0,004	0,607	1,599	0,024
70	0,358	-0,022	0,597	1,559	0,031
Average negative connectivity					
Age	ICC	CI-low	CI-high	F-stat.	p-value
54	0,672	0,438	0,809	3,05	<0,001
55	0,502	0,138	0,713	2,009	0,007
56	0,381	-0,078	0,645	1,616	0,045
58	0,466	0,094	0,685	1,873	0,01
59	0,575	0,263	0,755	2,352	0,001
60	0,441	0,046	0,672	1,787	0,017
61	0,4	0,032	0,629	1,668	0,018
62	0,719	0,535	0,83	3,554	<0,001
63	0,522	0,211	0,711	2,094	0,002
64	0,353	-0,041	0,598	1,546	0,036
65	0,544	0,283	0,71	2,195	<0,001
66	0,279	-0,202	0,567	1,386	0,104
67	0,15	-0,422	0,492	1,177	0,267
69	0,155	-0,345	0,47	1,184	0,238
70	0,439	0,106	0,648	1,782	0,008
Average positivie connectivity					
Age	ICC	CI-low	CI-high	F-stat.	p-value
54	0,677	0,447	0,812	3,099	<0,001
55	0,542	0,207	0,736	2,184	0,003
56	0,433	0,013	0,675	1,764	0,023
58	0,359	-0,089	0,622	1,559	0,05
59	0,551	0,221	0,741	2,225	0,002
60	0,554	0,24	0,739	2,244	0,002
61	0,376	-0,008	0,614	1,602	0,027
62	0,556	0,266	0,731	2,252	0,001
63	0,416	0,034	0,647	1,712	0,018
64	0,366	-0,02	0,606	1,578	0,03

65	0,454	0,141	0,653	1,832	0,005
66	0,468	0,113	0,681	1,878	0,008
67	-0,046	-0,752	0,375	0,956	0,569
69	0,102	-0,431	0,436	1,114	0,325
70	0,389	0,027	0,616	1,637	0,019

Number of negative connections

Age	ICC	CI-low	CI-high	F-stat.	p-value
54	0,417	0,001	0,66	1,716	0,025
55	0,533	0,192	0,731	2,143	0,003
56	0,254	-0,299	0,572	1,341	0,149
58	0,444	0,056	0,672	1,798	0,015
59	0,079	-0,595	0,469	1,086	0,384
60	0,257	-0,268	0,564	1,346	0,137
61	0,381	0	0,617	1,615	0,025
62	0,363	-0,052	0,615	1,571	0,039
63	0,573	0,294	0,742	2,342	0,001
64	0,367	-0,018	0,607	1,581	0,03
65	0,361	-0,005	0,594	1,565	0,026
66	0,184	-0,359	0,511	1,226	0,216
67	0,162	-0,402	0,5	1,194	0,249
69	0,287	-0,135	0,553	1,403	0,077
70	0,174	-0,316	0,482	1,211	0,209

Clustering coefficient - positive network

Age	ICC	CI-low	CI-high	F-stat.	p-value
54	0,191	-0,388	0,528	1,235	0,22
55	0,149	-0,474	0,509	1,175	0,281
56	0,305	-0,211	0,601	1,438	0,099
58	0,338	-0,124	0,61	1,511	0,063
59	-0,295	-1,243	0,253	0,772	0,823
60	-0,122	-0,914	0,342	0,891	0,664
61	0,12	-0,422	0,455	1,136	0,3
62	-0,345	-1,223	0,186	0,744	0,877
63	0,2	-0,323	0,516	1,249	0,192
64	-0,046	-0,684	0,35	0,956	0,574
65	0,178	-0,292	0,478	1,217	0,197
66	0,471	0,118	0,683	1,891	0,007
67	-0,613	-1,701	0,036	0,62	0,966
69	0,217	-0,247	0,509	1,278	0,15
70	0,348	-0,038	0,591	1,534	0,036

Global efficiency - positive network

Age	ICC	CI-low	CI-high	F-stat.	p-value

54	0,257	-0,273	0,567	1,347	0,139
55	0,115	-0,533	0,489	1,13	0,33
56	0,047	-0,661	0,453	1,049	0,433
58	0,164	-0,419	0,508	1,197	0,252
59	0,417	-0,009	0,664	1,716	0,027
60	0,54	0,215	0,73	2,172	0,002
61	0,273	-0,174	0,55	1,375	0,096
62	0,659	0,436	0,793	2,929	<0,001
63	0,585	0,314	0,749	2,411	<0,001
64	0,224	-0,249	0,518	1,288	0,148
65	0,24	-0,196	0,517	1,315	0,117
66	0,321	-0,132	0,592	1,472	0,069
67	-0,418	-1,374	0,153	0,705	0,909
69	0,123	-0,396	0,45	1,141	0,289
70	0,363	-0,015	0,6	1,57	0,029

Global efficiency - negative network

Age	ICC	CI-low	CI-high	F-stat.	p-value
54	-0,127	-0,932	0,343	0,888	0,669
55	0,404	-0,032	0,656	1,679	0,032
56	0,003	-0,736	0,428	1,003	0,495
58	0,293	-0,2	0,583	1,414	0,099
59	0,312	-0,191	0,603	1,454	0,09
60	0,118	-0,504	0,483	1,134	0,321
61	0,373	-0,012	0,612	1,596	0,028
62	0,423	0,046	0,651	1,732	0,016
63	0,401	0,01	0,638	1,669	0,023
64	0,304	-0,121	0,567	1,436	0,068
65	0,105	-0,407	0,431	1,118	0,315
66	0,1	-0,499	0,46	1,112	0,342
67	0,148	-0,426	0,491	1,174	0,27
69	-0,298	-1,068	0,185	0,77	0,865
70	0,322	-0,08	0,575	1,476	0,051

Multiplex clustering coefficient

Age	ICC	CI-low	CI-high	F-stat.	p-value
54	0,451	0,058	0,68	1,821	0,015
55	0,441	0,032	0,678	1,79	0,019
56	0,494	0,118	0,709	1,975	0,008
58	0,073	-0,574	0,454	1,078	0,389
59	0,189	-0,404	0,532	1,234	0,226
60	0,641	0,387	0,789	2,784	<0,001
61	0,284	-0,157	0,556	1,396	0,086
62	0,371	-0,039	0,62	1,591	0,035

63	0,654	0,428	0,791	2,891	<0,001
64	0,107	-0,437	0,445	1,12	0,319
65	0,397	0,052	0,617	1,659	0,014
66	0,077	-0,538	0,446	1,083	0,379
67	-0,331	-1,228	0,205	0,751	0,862
69	-0,219	-0,942	0,235	0,82	0,798
70	0,281	-0,145	0,549	1,391	0,082
Multiplex participation coefficient					
Age	ICC	CI-low	CI-high	F-stat.	p-value
54	0,28	-0,234	0,58	1,389	0,115
55	0,617	0,336	0,779	2,608	<0,001
56	0,396	-0,053	0,653	1,655	0,037
58	0,476	0,11	0,691	1,907	0,009
59	0,349	-0,128	0,624	1,536	0,063
60	0,419	0,008	0,659	1,72	0,023
61	0,267	-0,183	0,546	1,365	0,101
62	0,658	0,434	0,793	2,922	<0,001
63	0,014	-0,629	0,403	1,014	0,478
64	0,366	-0,02	0,606	1,578	0,03
65	0,478	0,178	0,668	1,914	0,003
66	-0,058	-0,763	0,365	0,945	0,586
67	-0,079	-0,807	0,355	0,926	0,615
69	0,32	-0,084	0,573	1,47	0,052
70	0,396	0,038	0,621	1,656	0,017
Multilayer clustering coefficient					
Age	ICC	CI-low	CI-high	F-stat.	p-value
54	0,485	0,117	0,7	1,942	0,008
55	0,545	0,212	0,738	2,199	0,003
56	0,044	-0,665	0,451	1,046	0,436
58	0,641	0,391	0,789	2,787	<0,001
59	0,461	0,066	0,689	1,855	0,014
60	0,536	0,208	0,728	2,154	0,003
61	0,277	-0,168	0,552	1,382	0,092
62	0,455	0,098	0,67	1,833	0,009
63	0,421	0,043	0,65	1,728	0,017
64	0,49	0,18	0,683	1,962	0,003
65	0,548	0,289	0,713	2,212	<0,001
66	0,563	0,272	0,738	2,29	0,001
67	0,339	-0,106	0,605	1,514	0,057
69	0,331	-0,065	0,58	1,496	0,045
70	0,501	0,206	0,687	2,006	0,002
Multilayer global efficiency					

Age	ICC	CI-low	CI-high	F-stat.	p-value
54	0,451	0,058	0,68	1,82	0,015
55	0,635	0,367	0,789	2,738	<0,001
56	0,341	-0,147	0,622	1,518	0,07
58	0,629	0,37	0,781	2,693	<0,001
59	0,358	-0,113	0,629	1,557	0,057
60	0,602	0,322	0,767	2,515	<0,001
61	0,395	0,022	0,625	1,652	0,02
62	0,583	0,31	0,747	2,396	<0,001
63	0,572	0,293	0,741	2,336	0,001
64	0,328	-0,082	0,582	1,488	0,051
65	0,513	0,234	0,69	2,054	0,001
66	0,316	-0,14	0,589	1,462	0,072
67	0,15	-0,423	0,492	1,177	0,267
69	0,274	-0,157	0,544	1,377	0,089
70	0,285	-0,14	0,551	1,398	0,079

The analysis was performed on the subset of subjects that had available longitudinal fMRI data. It was performed within the age groups that had at least 50 subjects. The F-statistics and p-values are calculated for a test under the null hypothesis that the ICC coefficient is equal to 0.

References

Cicchetti D. V., & Sparrow S. A. (1981). Developing criteria for establishing interrater reliability of specific items: Applications to assessment of adaptive behavior. *American Journal of Mental Deficiency*, 86(2), 127–137.

Zuo X. N., et al. (2014). An open science resource for establishing reliability and reproducibility in functional connectomics. *Scientific data*, 1(1), 1-13.
